# Renal toxicities in immune checkpoint inhibitors with or without chemotherapy: An observational, retrospective, pharmacovigilance study leveraging US FARES database

**DOI:** 10.1002/cam4.4343

**Published:** 2021-11-29

**Authors:** Fangyuan Hu, Yinghong Zhai, Lei Yuan, Jizhou Liang, Jinfang Xu, Xiaojing Guo, Xiang Zhou, Zhen Lin, Jinhai Sun, Xiaofei Ye, Jia He

**Affiliations:** ^1^ Department of Health Statistics Second Military Medical University Shanghai China; ^2^ Department of Medical Service Naval Hospital of Eastern theater Zhoushan China; ^3^ Tongji University School of Medicine Shanghai China; ^4^ Department of health management Second Military Medical University Shanghai China

**Keywords:** chemotherapy, disproportionality analysis, FAERS database, immune checkpoint inhibitors, renal toxicity

## Abstract

**Background:**

Immune checkpoint inhibitors (ICIs) have elicited durable antitumor responses in multiple types of cancers. However, ICIs could also induce potential toxicities that involve all organs, including renal system. In this study, we aimed to conduct a comprehensive description of the ICIs‐induced renal toxicities and the potential effects of chemotherapy.

**Methods:**

We conducted a pharmacovigilance study based on US Food and Drug Administration (FDA) Adverse Event Reporting System (FAERS) database between 01 January 2014 and 30 June 2019. Disproportionality analysis was used to assess the association between ICIs and renal adverse events (AEs), including reporting odds ratio (ROR) and information component (IC). ROR_025_ and IC_025_ are, respectively, 95% confidence interval lower end of ROR and IC. If the value of ROR_025_ exceeding one or IC_025_ higher than zero, then a signal was considered statistically significant.

**Results:**

A total of 30,602,758 reports were extracted from the database, with 4578 reports for ICIs‐associated renal AEs. Renal AEs were more frequently reported in anti‐PD‐1/PD‐L1 versus anti‐CTLA‐4 monotherapy group (ROR: 1.75, 95% CI: 1.52–2.01). Similarly, renal AEs were more commonly reported in ICIs polytherapy other than monotherapy group (ROR: 1.18, 95% CI: 1.10–1.27). Notably, ICIs plus chemotherapy strategies reported more renal toxicities compared to sole ICIs regimens (ROR: 1.30, 95% CI: 1.17–1.45), whereas exhibited lower fatality outcome rates. Importantly, acute kidney injury (1139, 24.88%) and renal failure (464, 10.14%) were the top two most commonly reported ICIs‐associated renal AEs, and also observed with the top two highest level of fatality outcome rates.

**Conclusions:**

A spectrum of renal AEs was detected in ICIs regimens and could be reinforced by ICIs combination. Compared to sole ICIs regimens, ICIs plus chemotherapy strategy reported more renal toxicities but lower fatality outcome rates. With the increasing popularity of ICIs especially combination strategies, it is vital important for clinicians to guarantee balance between durable clinical effects and potential renal toxicities in latest immunotherapy strategies.

## Graphical abstract

Our study shows a high reporting frequency of renal AEs induced by ICI monotherapy (especially by anti‐PD‐1/PD‐L1 monotherapy) and reinforced by combination of ICI regimens. Notably, ICIs plus chemotherapy were more frequently associated with renal toxicities but lower fatality outcome rates compared to sole ICIs regimens. In conclusion, it is vital critical for clinicians to fully recognize ICIs‐associated renal toxicities, seek balance between durable clinical effects and potential fatal toxicities in latest immunotherapy strategies.

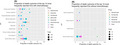

## 1 INTRODUCTION

Immune checkpoint inhibitors (ICIs) have shown remarkable clinical benefits in multiple cancer types and became a mainstay of cancer treatment.^1^, ^2^ Approved ICIs include cytotoxic T lymphocyte antigen‐4 (CTLA‐4), anti‐programmed cell death 1 (PD‐1), and anti‐programmed cell death ligand 1 (PD‐L1). Currently, ICIs have improved clinical outcomes in non‐small cell lung cancer,^1^ renal cell cancer,^3^ urothelial carcinoma,^4^ Hodgkin’s disease,^5^ hepatocellular carcinoma,^2^ Merkel carcinoma,^6^ esophageal cancer,^7^ etc. However, ICIs could also induce immune‐related adverse events (irAEs) that involve all organs, including renal system. Increasing case series have reported limited numbers of renal irAEs including acute kidney injury,^8^ glomerulonephritis,^9^ interstitial nephritis,^10^ acute renal failure,^11^ etc. Notably, some of the renal irAEs could cause severe or even fatal outcomes,^12^ whereas it has been less frequently reported than other system irAEs^13^ and have not been extensively characterized. Similarly, potential effect of chemotherapy on ICIs was also unclear and had never been explored. Given the increasing cancer patients expected to be treated with ICIs in the following years, more attention is warranted for these renal toxicity problems. What is more, in novel clinical trials,^1^, ^7^ increasing administration of ICIs was combined with chemotherapies, and potential risk for this change had never been accessed by any prior research.

In this study, we aimed to systematically characterize total and class‐specific ICIs‐associated renal toxicities, explore the potential effect of chemotherapies on ICIs‐associated renal toxicities, and provide evidence for clinical practice.

## 2 MATERIALS AND METHODS

### 2.1 Study design and participants

An observational, retrospective, pharmacovigilance study was conducted based on US Food and Drug Administration (FDA) Adverse Event Reporting System (FAERS) database from 01 January 2014 to 30 June 2019. FAERS database is a free available post‐marketing surveillance database managed by FDA, which contains millions of spontaneous adverse event reports submitted by individual patients, lawyers, physicians, drug companies, hospitals, etc.^14^ Data management was conducted to guarantee there were no duplicated drug‐to‐AEs records, records which miss necessary variables like drug name or AEs were also dropped. All the data can be available at https://fis.fda.gov/extensions/FPD‐QDE‐FAERS/FPD‐QDE‐FAERS.html.

For each records, variables like age, gender, outcomes, drug names, and preferred term (PT, variable for standard AEs) were extracted from the database. PT is a middle level of Medical Dictionary for Regulatory Activities (MedDRA) (Version 22.0 English),^15^ which was used by FDA to determinate the standard AEs. System Organ Class (SOC) is the top level in MedDRA system, and all the PTs below the SOC of renal and urinary system were searched in FAERS database. Included PTs (with at least one record in the database) can be seen in supplementary material (Table S1).

ICIs in our study include anti‐PD‐1 antibodies (nivolumab (opdivo), cemiplimab (libtayo), and pembrolizumab (keytruda)), anti‐PD‐L1 antibodies (atezolizumab (tecentriq), avelumab (bavencio), and durvalumab (imfinzi)), and anti‐CTLA‐4 antibodies (ipilimumab (yervoy) and tremelimumab). To our knowledge, combination with chemotherapy drugs could also affect ICIs clinical effect and toxicities. Thus combined chemotherapy drugs (40 drugs) were also considered in our studies. Both standard and original drug names were used to identify class‐specific ICIs and chemotherapy drugs in FAERS database. Details for these standard and original drug names could be seen in supplementary material (Tables S2 and S3).

### 2.2 Statistical analysis

Disproportionality analysis was used in our study to assess whether suspected target renal AEs were differentially reported in ICIs compared to other drugs among the full database.^16^ Disproportionality analysis is a method that compare the proportion of target AEs in target drugs to the proportion of the same AEs in control group drugs. If the proportion of target AEs in suspected drugs is higher, then a potential drug safety signal is detected. Both information component (IC) and reporting odds ratio (ROR) were calculated in disproportionality analysis in our study, which were frequently used in pharmacovigilance studies among the database with millions of records.^17^, ^18^ ROR_025_ and IC_025_ are, respectively, 95% confidence interval lower end of ROR and IC. The value of ROR_025_ exceeding one or IC_025_ exceeding zero was deemed statistically significant to detect a signal. Drug‐ADR combinations with at least three reports were considered in our study. Disproportionality analysis was also used to compare renal toxicities on different regimens: male versus female, younger group (age <60) versus older group (age >=60), anti‐PD‐1/anti‐PD‐L1 versus anti‐CTLA‐4 monotherapy, ICIs monotherapy (anti‐PD‐1/anti‐PD‐L1 and anti‐CTLA‐4) versus ICIs combination (anti‐PD‐1/anti‐PD‐L1 plus anti‐CTLA‐4), and ICIs alone versus ICIs plus chemotherapy. Only ROR was calculated to compare renal toxicities among different regimens. Venn diagram was used to explore cancer types and complications in more frequently reported AEs and provide valuable indications. The overlapping of some areas can show the common characters of different subgroups.

In order to get more stable signals, shrinkage transformation model was conducted based on disproportionality analysis. Related statistical formula is as follows^19^:

$$\mathrm{ROR}=\left(N_{\mathrm{Observed}}+0.5\right)/\left(\left(\left(N_{\mathrm{Drug}}*N_{\mathrm{Event}}\right)/N_{\mathrm{Total}}\right)+0.5\right)$$

$$\mathrm{IC}=\left(N_{\mathrm{Observed}}+0.5\right)/\left(\left(\left(N_{\mathrm{Drug}}*N_{\mathrm{Event}}\right)/N_{\mathrm{Total}}\right)+0.5\right)$$

$N_{\mathrm{Observed}}$ is the number of observed target drug AEs records, $N_{\mathrm{Drug}}$ is the number of any target drugs‐associated AEs records, $N_{\mathrm{Event}}$ is the number of target AEs records, and $N_{\mathrm{Total}}$ is the total number of any AEs records in any drugs.

All analyses were conducted using the software SAS 9.4 (SAS Institute) and R (version 3.4.3), and the results were calculated and checked by two different group members (Fangyuan Hu & Yinghong Zhai). All the data in the analysis can be available at https://fis.fda.gov/extensions/FPD‐QDE‐FAERS/FPD‐QDE‐FAERS.html.

## 3 RESULTS

### 3.1 Baseline of ICIs‐associated renal AEs

A total of 30,602,758 reports were extracted from the database (from 01 January 2014 to 30 June 2019), with 4578 reports for ICIs‐associated renal AEs. Baseline of renal AEs for ICIs and control group is presented in Table 1. Male (55.26%) and the older group (42.73%) account more in all types of ICIs‐associated renal AEs. Notably, most ICIs‐associated renal AEs were reported in the year of 2016–2019 (84.03%), reflecting the significant increased popularity of ICIs in recent years. The death outcome of proportion ICIs‐associated renal AEs was 18.50%, significant higher than those renal AEs related to other drugs (8.73%).

**TABLE 1:** Baseline of renal AEs for ICIs and control group in FAERS database

Character	Renal AEs in any other drugs (1104288)	Renal AEs in ICIs (4578)
Gender		
Male	350457(31.74)	2530(55.26)
Female	462724(41.90)	1434(31.32)
Missing	291107(26.36)	614(13.41)
Age		
<65	309844 (28.06)	1493 (32.61)
>=65	352760 (31.94)	1956 (42.73)
Missing	441684 (40.00)	1129 (24.66)
Year		
2014	51463 (4.66)	44 (0.96)
2015	105145 (9.52)	18 (0.39)
2016	162576 (14.72)	669 (14.61)
2017	161853 (14.66)	901 (19.68)
2018	385852 (34.94)	1773 (38.73)
2019 Q1‐Q2	237399 (21.50)	1173 (25.62)
Outcome		
Death	96432(8.73)	847(18.50)
Life‐threatening	34753(3.15)	216(4.72)
Disability	23340(2.11)	51(1.11)
Hospitalization	332388(30.10)	1748(38.18)
Congenital anomaly	1095(0.10)	2(0.04)
Other serious (important medical events)	447951(40.56)	1119(24.44)
Required intervention to prevent permanent impairment/damage	335(0.03)	1(0.02)
Missing	167994(15.21)	594(12.98)
Report countries		
United States	702909(63.65)	1587(34.67)
Japan	49446(4.48)	835(18.24)
France	45733(4.14)	428(9.35)
Germany	30079(2.72)	280(6.12)
Italy	16263(1.47)	135(2.95)
Great Britain	47726(4.32)	133(2.91)
Canada	35314(3.20)	106(2.32)
Spain	11241(1.02)	105(2.29)
Australia	6876(0.62)	88(1.92)
Netherlands	6176(0.56)	57(1.25)
Others	118406(10.72)	591(12.91)
Missing	34119(3.09)	233(5.09)

### 3.2 Association between total ICIs monotherapy/polytherapy (with and without chemotherapy) and renal AEs

Association between total ICIs monotherapy/polytherapy (with and without chemotherapy) and renal AEs is shown in Table 2. Interestingly, in ICIs without chemotherapy strategies, signals were only significant in atezolizumab monotherapy (ROR_025_: 1.37) and pembrolizumab+ipilimumab (ROR_025_: 1.05) group. In ICIs plus chemotherapy strategies, signals were detected in atezolizumab (ROR_025_: 1.00), ipilimumab+nivolumab (ROR_025_: 1.29), and durvalumab+tremelimumab (ROR_025_: 1.71). Renal toxicities were more frequently reported in patients treated with anti‐PD‐1/PD‐L1 versus those treated with anti‐CTLA‐4 (ROR_025_: 1.52), for those treated with ICIs polytherapy versus ICIs monotherapy (ROR_025_: 1.10), and for those treated with ICIs plus chemotherapy versus sole ICIs (ROR_025_: 1.17) strategies (Table 2). We consider the negative results in class‐specific ICIs regimens to be reasonable since too much bias was not excluded. Thus, association between class‐specific ICIs monotherapy/polytherapy and renal AEs was assessed in further analysis. Only ROR_025_ values of the top 10 most frequently reported renal AEs in the database are shown in the manuscript (Tables S3 and S4), other detailed signals can be seen in supplement materials (Tables S4–S7).

**TABLE 2:** ROR_025_ values between total ICIs monotherapy/polytherapy (with and without chemotherapy) and renal AEs in FAERS database

Drug	a	b	c	d	ROR	ROR_025_	ROR_975_	IC	IC_025_	IC_975_
Total	4578	138168	1104288	29367235	−0.18	−0.22	−0.13	0.88	0.86	0.91
ICIs										
Nivolumab	1838	56234	1107028	29437715	0.87	0.83	0.91	−0.20	−0.27	−0.12
Pembrolizumab	851	28811	1108015	29465138	0.79	0.73	0.84	−0.34	−0.45	−0.22
Cemiplimab	6	188	1108860	29493761	0.86	0.38	1.94	−0.21	−1.70	1.27
Atezolizumab	337	5861	1108529	29488088	1.53	**1.37**	1.71	0.58	**0.40**	0.76
Avelumab	28	833	1108838	29493116	0.90	0.61	1.31	−0.15	−0.79	0.48
Durvalumab	58	2327	1108808	29491622	0.66	0.51	0.86	−0.57	−1.01	−0.13
Ipilimumab	203	10940	1108663	29483009	0.49	0.43	0.57	−0.99	−1.22	−0.76
Poly1	18	316	1108848	29493633	1.49	0.93	2.40	0.55	−0.25	1.36
Poly2	798	22428	1108068	29471521	0.95	0.88	1.02	−0.08	−0.19	0.04
Poly3	37	672	1108829	29493277	1.46	**1.05**	2.03	0.52	−0.03	1.07
Anti‐PD−1/PD‐L1 vs. anti‐CTLA−4	3118	94254	203	10940	1.78	**1.54**	2.06			
Polytherapy vs. Monotherapy	853	23484	3322	105228	1.15	**1.07**	1.24			
ICIs+chemotherapy										
Nivolumab	103	2481	1108763	29491468	1.10	0.91	1.34	0.14	−0.19	0.46
Pembrolizumab	49	1723	1108817	29492226	0.76	0.57	1.01	−0.39	−0.86	0.09
Atezolizumab	93	2005	1108773	29491944	1.23	**1.00**	1.52	0.29	−0.06	0.63
Avelumab	15	364	1108851	29493585	1.09	0.65	1.83	0.12	−0.76	1.01
Durvalumab	11	547	1108855	29493402	0.55	0.30	0.99	−0.85	−1.90	0.20
Ipilimumab	12	299	1108854	29493650	1.06	0.60	1.90	0.09	−0.91	1.09
Poly2	76	1234	1108790	29492715	1.63	**1.29**	2.06	0.67	**0.29**	1.05
Poly4	18	164	1108848	29493785	2.78	**1.71**	4.52	1.38	**0.58**	2.19
Anti‐PD−1/PD‐L1 vs. anti‐CTLA−4	271	7120	12	299	0.95	0.53	1.71			
Polytherapy vs. Monotherapy	94	1509	284	7439	1.63	**1.28**	2.07			
Total										
Anti‐PD−1/PD‐L1 vs. anti‐CTLA−4	3389	101374	215	11239	1.75	**1.52**	2.01			
Polytherapy vs. Monotherapy	947	24993	3606	112667	1.18	**1.10**	1.27			
ICIs+chemotherapy vs. ICIs	378	8948	4175	128712	1.30	**1.17**	1.45			

Note

In the table, a is the number of records reported with any ICIs and renal AEs, b is the number of records reported with any ICIs and without renal AEs, c is the number of records reported with any other drugs and renal AEs, and d is the number of records reported with any other drugs and without renal AEs. Poly1, poly2, poly3, and poly4 represent pembrolizumab+ipilimumab+ nivolumab, ipilimumab+nivolumab, pembrolizumab+ipilimumab, and durvalumab+tremelimumab, respectively. Bold values indicate the signals with statistical significance.

### 3.3 Association between class‐specific ICIs monotherapy/polytherapy (with and without chemotherapy) and renal AEs

Class‐specific signals between ICIs monotherapy/polytherapy and renal AEs are shown in Tables 3 and 4. In ICIs without chemotherapy group, signals were detected in acute kidney injury AE for most of the ICIs strategies except cemiplimab and avelumab. No signals were detected in renal failure, urinary tract infection, chronic kidney disease, and urinary retention for any of the ICIs strategies. Notably, nephritis had the most significant signals in all ICIs strategies compared to any other renal AEs (Table 3). Interestingly, renal toxicities were over reported in ICIs polytherapy, which was also observed in most class‐specific renal AEs. For instance, acute kidney injury was more frequently reported in nivolumab+ipilimumab group (ROR_025_: 3.19) than nivolumab (ROR_025_: 1.66) and ipilimumab (ROR_025_: 1.04) monotherapy group (Table 3). In ICIs plus chemotherapy group, signals for acute kidney injury were also significant in most of the ICIs strategies except avelumab and durvalumab, and these signals were considered to be the most significant in all ICI regimens compared to other renal AEs except atezolizumab and avelumab monotherapy (Table 4). Similarly, renal impairment and chronic kidney disease had negative results in all ICIs regimens.

**TABLE 3:** ROR_025_ values between class‐specific ICIs monotherapy/polytherapy (without chemotherapy) and the top 10 most frequently reported renal AEs in FAERS database

PT	Nivo	Pemb	Cemi	Atez	Avel	Durv	Ipil	Poly2	Poly3
Acute kidney injury	**1.66**	**1.28**	0.97	**3.47**	0.66	**2.19**	**1.04**	**3.19**	**1.70**
Renal failure	0.68	0.48		0.76	0.80		0.11	0.57	
Renal impairment	**1.06**	**1.18**		0.69			0.49	0.33	
Urinary tract infection	0.34	0.21		0.63			0.10	0.48	
Tubulointerstitial nephritis	0.80	**1.31**		0.92			0.59	**1.38**	
Chronic kidney disease	0.27	0.15		0.40		0.33	0.26	0.10	
Nephritis	**8.94**	**15.15**		**12.68**			**1.47**	**11.20**	
Hematuria	0.81	0.70		**3.09**			0.43	0.56	
Urinary retention	0.65	0.51		0.90			0.18	0.19	
Chromaturia	0.67	0.65					**1.24**	**2.44**	

Note

In the table, nivo, pemb, cemi, atez, avel, durv, ipil, poly1, poly2, and poly3, represent nivolumab, pembrolizumab, cemiplimab, atezolizumab, avelumab, durvalumab, ipilimumab, nivolumab+ipilimumab+ pembrolizumab, nivolumab+ipilimumab, and ipilimumab+pembrolizumab, respectively. Bold values indicate the signals with statistical significance.

**TABLE 4:** ROR_025_ values between class‐specific ICIs monotherapy/polytherapy (with chemotherapy) and the top 10 most frequently reported renal AEs in FAERS database

PT	Nivo	Pemb	Atez	Avel	Durv	Ipil	Poly2
Acute kidney injury	**3.68**	**1.31**	**2.69**	0.94	0.95	**1.08**	**6.62**
Renal failure	0.14	0.41	0.49	0.50			**1.44**
Renal impairment	0.20	0.91	0.78				
Urinary tract infection			0.50	**1.63**			0.36
Tubulointerstitial nephritis			**2.38**				
Chronic kidney disease	0.10						
Nephritis			**2.71**				

Note

In the table, nivo, pemb, cemi, atez, avel, durv, ipil, poly1, poly2, and poly3, represent nivolumab, pembrolizumab, cemiplimab, atezolizumab, avelumab, durvalumab, ipilimumab, nivolumab+ipilimumab+ pembrolizumab, nivolumab+ipilimumab, and ipilimumab+pembrolizumab, respectively. Bold values indicate the signals with statistical significance.

### 3.4 Proportion of death outcome in class‐specific renal AEs

**FIGURE 1:**
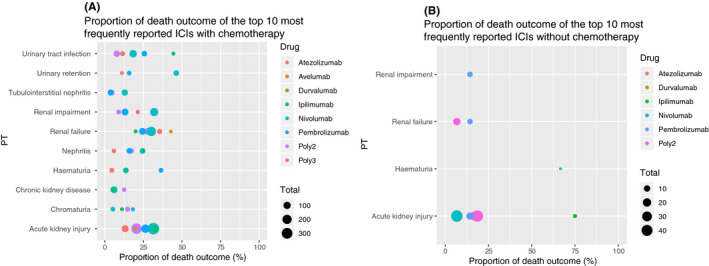
(A) Proportion of death outcome of the top 10 most frequently reported ICIs with chemotherapy. (B) Proportion of death outcome of the top 10 most frequently reported ICIs without chemotherapy

Totally, acute kidney injury and renal failure were considered to have the top two highest level proportion of death outcome in all ICIs regimens (Figure 1A,B). In ICIs without chemotherapy group, nivolumab monotherapy was associated with the highest level proportion of death outcome among the top 10 most frequently reported renal AEs. Interestingly, higher fatality outcome rates were observed in patients treated with ICIs regimens without chemotherapy compared to those with chemotherapy. For instance, proportion of death outcome for sole nivolumab‐associated acute kidney injury was 6.67%, while combined with chemotherapy this proportion increased to 31.51%.

### 3.5 Further analysis for the top two most frequently reported renal AEs

Since acute kidney injury (1139, 24.88%) and renal failure (464, 10.14%) account 35.02% of all ICIs‐associated renal AEs records, we further explored difference in potential cancer types and complications in death cases for this two AEs.

Different cancers were rarely overlapping in both ICIs with or without chemotherapy groups (Figures S1 and S2). For ICIs with chemotherapy group, in acute kidney injury death cases, cancer types for most patients were malignant melanoma (51/252) and non‐small cell lung cancer (NSCLC) (42/252) (Figure S1A). Similarly, in renal failure death cases, most overlapping were also happened in NSCLC (26/122) and malignant melanoma (19/122) patients (Figure S1C). For ICIs with chemotherapy group, in acute kidney injury death cases, overlapping mostly occurred in NSCLC (10/20) (Figure S1B), while renal failure death cases were often reported with Hodgkin’s disease (Figure S1D).

## 4 DISCUSSION

In addition to durable antitumor responses and remarkable clinical effects in multiple types of cancers, ICIs could induce system toxicities like renal toxicities, and their outcomes had not been systematically characterized. To our knowledge, our study was the first and the biggest study that gives a comprehensive description of the ICIs‐induced renal toxicities. We also took chemotherapy into consideration. Given the increasing administration of ICIs in the recent years, it is vital important to extensively characterize renal toxicities for clinical oncologists. There were mainly five findings observed in our research.

First, renal AEs reporting frequency differed between anti‐PD‐1/PD‐L1 and anti‐CTLA‐4 immunotherapies. It seems like renal toxicities were more frequently reported in patients treated with anti‐PD‐1/PD‐L1 versus those treated with anti‐CTLA‐4. In addition, a total of 129 class‐specific signals were significant in anti‐PD‐1/PD‐L1 classes compared to 18 signals in anti‐CTLA‐4. Notably, similar trend was also observed in neurologic toxicities^18^ and cardiovascular toxicities.^17^ Importantly, true precise mechanisms for these toxicities difference were still unclear. With the increasing use of anti‐PD‐1/PD‐L1 agents, more awareness needs to be raised for these AEs.

Second, combination of ICIs regimens would reinforce renal toxicities. In our analysis, renal toxicities were more commonly reported in patients treated with ICIs polytherapy than those treated with ICIs monotherapy. Interestingly, similar results were also detected in further class‐specific AEs. For instance, compared to nivolumab and ipilimumab monotherapy, acute kidney injury was more frequently reported in nivolumab+ipilimumab group. Importantly, trend of ICIs combination was observed in some novel clinical trials, and directly observed treatment‐related grade 3 or higher adverse events were more common in combination group than those in monotherapy group.^20^ Thus, it is highly important for oncologists to maintain the balance between fascinating clinical effects and potential fatal toxicities.

Third, ICIs plus chemotherapy were more frequently associated with renal toxicities compared to sole ICIs regimens. Similar results were also observed in some class‐specific renal AEs like acute kidney injury. Notably, some latest studies indicated that more toxicities were observed in ICIs plus chemotherapy group instead of sole ICIs group, which caters to our findings.^21^ Although frequently mentioned in latest clinical trials, toxicities in ICIs plus chemotherapy group were always given a brief short description other than extensive research.^22^, ^23^ What is more, in prior ICIs‐associated pharmacovigilance studies,^17^, ^18^ the potential effect of chemotherapy on ICIs was not even mentioned. Thus, what we had detected was cater to the latest needs and could provide benefit evidence for clinical practice.

Fourth, a spectrum of renal AEs was detected in different ICIs, with the outcomes differed. A total of 201 and 38 signals were retrospectively detected in ICIs with chemotherapy and without chemotherapy groups, with most to be found for the first time. Interestingly, in most renal AEs, a lower fatality outcome rates were observed in patients treated with ICIs regimens without chemotherapy compared to those with chemotherapy, which suggested that compared to ICIs, ICIs plus chemotherapy may bring more remarkable clinical advantages. Similarly, novel trials has proved that ICIs plus chemotherapy has significantly longer overall survival and progression‐free survival versus chemotherapy^7^, ^22^, ^23^ or immunotherapy alone.^21^ However, balances also need to be considered between durable clinical improvement and potential complications. Notably, renal toxicities may not be so significant compared to cancers that call for ICIs plus chemotherapy, which means doctors need to take positive treatments despite renal toxicities. However, it is also vital important for clinicians to recognize ICIs‐associated renal toxicities in latest immunotherapy strategies and take enough preparations in ICIs plus chemotherapy treatment.

Fifth, acute kidney injury and renal failure were observed to be the top two most frequently reported renal AEs. Importantly, these two AEs were also considered to report the highest level of fatality outcome rate. In further analysis, most overlapping death cases for this two AEs were occurred in NSCLC and malignant melanoma. That is reasonable since increasing latest ICIs strategies were applied in this two cancers.^22^, ^24^ Of equal importance, some more commonly happened fatal complications like sepsis, anemia, hepatic failure, and respiratory failure also need to be aware of by the clinicians.

Our study had some limitations. First, this is an observational, retrospective real‐world study and the incidence of these renal AEs cannot be determined. Second, too much missing data were occurred in some important variables like age and gender, thus further analysis was not conducted on these variables. Third, detail demographic variables and clinical information were not included in FAERS database which could help to conduct further clinical evaluations. Fourth, cancer types were not reported to the database, thus class‐specific drug to cancer conclusions were not available. Last but not least, some PTs have similar clinical meanings, which may cause unnecessary confusions, while PT is also considered as a standard level that truly represent the most significant differences.

## 5 CONCLUSIONS

A spectrum of renal AEs was detected in different ICIs, especially in anti‐PD‐1/PD‐L1 agents and could be reinforced by combination of ICIs regimens. Notably, compared to sole ICIs regimens, ICIs plus chemotherapy were more frequently associated with renal toxicities but lower fatality outcome rates. With the increasing administration of ICIs especially combination strategies, it is vital important for clinicians to recognize ICIs‐associated renal toxicities in latest immunotherapy strategies.

## CONFLICT OF INTEREST

The authors declare no potential conflict of interest.

## AUTHOR CONTRIBUTIONS

Concept and design: Jia He, Fangyuan Hu, and Lei Yuan designed the study. Fangyuan Hu, Xiaofei Ye, Yinghong Zhai, and Lei Yuan controlled the quality of data and performed the statistical analysis. Fangyuan Hu, Yinghong Zhai, Jinhai Sun, Jinfang Xu, and Xiaojing Guo managed and checked all the data. Jia He, Fangyuan Hu, Zhen Lin, and Yinghong Zhai contributed to the manuscript preparation, editing, and review. Fangyuan Hu, Yinghong Zhai, Xiaofei Ye, Lei Yuan, and Jizhou Liang took part in the manuscript revision. All authors read, checked, and approved the final manuscript.

## Supporting information

Fig S1

Fig S2

Supplementary Material

## Data Availability

All the data in our analysis can be available in https://fis.fda.gov/extensions/FPD‐QDE‐FAERS/FPD‐QDE‐FAERS.html.
