# Supplementary material for: Renal toxicities in immune checkpoint inhibitors with or without chemotherapy: An observational, retrospective, pharmacovigilance study leveraging US FARES database
Source: Cancer Med. 2021 Nov 29;10(24):8754–62. doi: 10.1002/cam4.4343 (PMC8683558; doi:10.1002/cam4.4343)
Supplement: Supplementary file 3 — Supplementary Material [file CAM4-10-8754-s002.docx]

[Table S1 Included PT below the SOC of renal and urinary disorders 2](#_Toc23436511)

[Table S2 Standard drug name and original ICIs drug name in FAERS database 6](#_Toc23436512)

[Table S3 Standard drug name and original ICIs chemotherapy drug name in FAERS database 7](#_Toc23436513)

[Table S4 ROR025 of the association between class-specific ICIs monotherapy/polytherapy (without chemotherapy) and renal AEs in FAERS database 30](#_Toc23436514)

[Table S5 ROR025 of the association between class-specific ICIs monotherapy/polytherapy (with chemotherapy) and renal AEs in FAERS database 35](#_Toc23436515)

[Table S6 IC025 of the association between class-specific ICIs monotherapy/polytherapy (without chemotherapy) and renal AEs in FAERS database 36](#_Toc23436516)

[Table S7 IC025 of the association between class-specific ICIs monotherapy/polytherapy (with chemotherapy) and renal AEs in FAERS database 41](#_Toc23436517)

[Figure S1 A. Venn diagram between acute kidney injury and the death outcome cases in the top 4 most frequently related cancers (without chemotherapy). B. Venn diagram between acute kidney injury and the death outcome cases in the top 2 most frequently related cancers (with chemotherapy). C. Venn diagram between renal failure and the death outcome cases in the top 4 most frequently related cancers (without chemotherapy). D. Venn diagram between renal failure and the death outcome cases in the top 1 most frequently related cancers (with chemotherapy).. 43](#_Toc23436518)

[Figure S2 A. Venn diagram between acute kidney injury and the death outcome cases in the top 4 most frequently related AEs (without chemotherapy). B. Venn diagram between acute kidney injury and the death outcome cases in the top 3 most frequently related AEs (with chemotherapy). C. Venn diagram between renal failure and the death outcome cases in the top 4 most frequently related AEs (without chemotherapy). D. Venn diagram between renal failure and the death outcome cases in the top 2 most frequently related AEs (with chemotherapy). 44](#_Toc23436519)

# Table S1 Included PT below the SOC of renal and urinary disorders

| **HLGT** | **HLT** | **PT** | **Frequency** |
| --- | --- | --- | --- |
| Bladder and bladder neck disorders (excl calculi) | Bladder disorders NEC | BLADDER DILATATION | 1 |
|  |  | BLADDER DISORDER | 18 |
|  |  | BLADDER HYPERTROPHY | 2 |
|  |  | BLADDER MASS | 16 |
|  |  | BLADDER NECROSIS | 1 |
|  |  | BLADDER OBSTRUCTION | 2 |
|  |  | BLADDER PERFORATION | 4 |
|  |  | ENTEROVESICAL FISTULA | 3 |
|  |  | URINARY BLADDER HAEMORRHAGE | 6 |
|  |  | VESICAL FISTULA | 1 |
|  | Bladder infections and inflammations | CYSTITIS | 68 |
|  |  | CYSTITIS ESCHERICHIA | 1 |
|  |  | CYSTITIS HAEMORRHAGIC | 13 |
|  |  | CYSTITIS INTERSTITIAL | 5 |
|  |  | CYSTITIS NONINFECTIVE | 10 |
|  | Bladder neoplasms | BLADDER ADENOCARCINOMA RECURRENT | 1 |
|  |  | BLADDER CANCER | 34 |
|  |  | BLADDER CANCER RECURRENT | 3 |
|  |  | BLADDER CANCER STAGE IV | 1 |
|  |  | BLADDER NEOPLASM | 4 |
|  |  | BLADDER TRANSITIONAL CELL CARCINOMA | 1 |
|  |  | METASTASES TO BLADDER | 1 |
|  |  | METASTATIC CARCINOMA OF THE BLADDER | 3 |
|  | Myoneurogenic bladder disorders | ATONIC URINARY BLADDER | 1 |
|  |  | BLADDER DYSFUNCTION | 1 |
|  |  | HYPERTONIC BLADDER | 1 |
|  |  | NEUROGENIC BLADDER | 13 |
| Genitourinary tract disorders NEC | Genital and urinary tract disorders NEC | FEMALE GENITAL TRACT FISTULA | 7 |
|  |  | TRANSITIONAL CELL CANCER OF THE RENAL PELVIS AND URETER | 1 |
|  |  | TRANSITIONAL CELL CARCINOMA | 8 |
|  |  | TRANSITIONAL CELL CARCINOMA METASTATIC | 1 |
|  |  | TRANSITIONAL CELL CARCINOMA RECURRENT | 1 |
|  |  | URINARY TRACT DISORDER | 6 |
|  |  | URINARY TRACT NEOPLASM | 1 |
|  |  | URINARY TRACT OBSTRUCTION | 20 |
|  |  | UROGENITAL DISORDER | 1 |
|  |  | UROGENITAL FISTULA | 2 |
|  | Genitourinary tract infections and inflammations NEC | ESCHERICHIA URINARY TRACT INFECTION | 5 |
|  |  | GENITOURINARY TRACT INFECTION | 1 |
|  |  | URINARY TRACT INFECTION | 281 |
|  |  | URINARY TRACT INFECTION ENTEROCOCCAL | 2 |
|  |  | URINARY TRACT INFLAMMATION | 1 |
|  |  | UROSEPSIS | 37 |
| Nephropathies | Glomerulonephritis and nephrotic syndrome | C3 GLOMERULOPATHY | 1 |
|  |  | FOCAL SEGMENTAL GLOMERULOSCLEROSIS | 3 |
|  |  | GLOMERULONEPHRITIS | 14 |
|  |  | GLOMERULONEPHRITIS ACUTE | 1 |
|  |  | GLOMERULONEPHRITIS MEMBRANOUS | 8 |
|  |  | GLOMERULONEPHRITIS MINIMAL LESION | 2 |
|  |  | GOODPASTURE'S SYNDROME | 2 |
|  |  | GRANULOMATOSIS WITH POLYANGIITIS | 5 |
|  |  | HENOCH-SCHONLEIN PURPURA NEPHRITIS | 3 |
|  |  | IGA NEPHROPATHY | 12 |
|  |  | NEPHRITIC SYNDROME | 1 |
|  |  | NEPHRITIS ALLERGIC | 3 |
|  |  | NEPHROTIC SYNDROME | 43 |
|  | Nephritis NEC | AUTOIMMUNE NEPHRITIS | 39 |
|  |  | LUPUS NEPHRITIS | 2 |
|  |  | NEPHRITIS | 154 |
|  |  | TUBULOINTERSTITIAL NEPHRITIS | 205 |
|  | Nephropathies and tubular disorders NEC | DIABETIC NEPHROPATHY | 7 |
|  |  | GLOMERULONEPHROPATHY | 2 |
|  |  | GLOMERULOSCLEROSIS | 3 |
|  |  | NEPHROGENIC DIABETES INSIPIDUS | 1 |
|  |  | NEPHROPATHY | 20 |
|  |  | NEPHROPATHY TOXIC | 25 |
|  |  | NEPHROSCLEROSIS | 5 |
|  |  | RENAL AMYLOIDOSIS | 1 |
|  |  | RENAL TUBULAR ACIDOSIS | 7 |
|  |  | RENAL TUBULAR ATROPHY | 5 |
|  |  | RENAL TUBULAR DISORDER | 13 |
|  |  | RENAL TUBULAR DYSFUNCTION | 2 |
| Renal disorders (excl nephropathies) | Renal disorders NEC | COMPLICATIONS OF TRANSPLANTED KIDNEY | 2 |
|  |  | FLUID RETENTION | 32 |
|  |  | KIDNEY FIBROSIS | 15 |
|  |  | KIDNEY TRANSPLANT REJECTION | 47 |
|  |  | RENAL DISORDER | 149 |
|  |  | RENAL HAEMATOMA | 1 |
|  |  | RENAL HAEMORRHAGE | 14 |
|  |  | RENAL MASS | 2 |
|  |  | RENAL TRANSPLANT FAILURE | 8 |
|  | Renal failure and impairment | ACUTE KIDNEY INJURY | 1139 |
|  |  | ANURIA | 15 |
|  |  | CHRONIC KIDNEY DISEASE | 182 |
|  |  | END STAGE RENAL DISEASE | 53 |
|  |  | HAEMOLYTIC URAEMIC SYNDROME | 10 |
|  |  | HEPATORENAL FAILURE | 4 |
|  |  | HEPATORENAL SYNDROME | 4 |
|  |  | OLIGURIA | 1 |
|  |  | POSTRENAL FAILURE | 5 |
|  |  | PRERENAL FAILURE | 15 |
|  |  | RENAL FAILURE | 464 |
|  |  | RENAL IMPAIRMENT | 303 |
|  |  | RENAL INJURY | 68 |
|  | Renal failure complications | AZOTAEMIA | 9 |
|  |  | HYPERPARATHYROIDISM SECONDARY | 6 |
|  |  | LOW TURNOVER OSTEOPATHY | 1 |
|  |  | NEPHROGENIC ANAEMIA | 11 |
|  |  | OSTEOMALACIA | 2 |
|  | Renal hypertension and related conditions | HYPERTENSIVE NEPHROPATHY | 1 |
|  |  | ESCHERICHIA PYELONEPHRITIS | 1 |
|  |  | KIDNEY INFECTION | 17 |
|  |  | POLYOMAVIRUS-ASSOCIATED NEPHROPATHY | 1 |
|  |  | PYELITIS | 1 |
|  |  | PYELONEPHRITIS | 33 |
|  |  | PYELONEPHRITIS ACUTE | 3 |
|  | Renal neoplasms | CLEAR CELL RENAL CELL CARCINOMA | 1 |
|  |  | METASTASES TO KIDNEY | 10 |
|  |  | METASTATIC RENAL CELL CARCINOMA | 3 |
|  |  | NEPHROBLASTOMA | 2 |
|  |  | NON-RENAL CELL CARCINOMA OF KIDNEY | 1 |
|  |  | RENAL CANCER | 8 |
|  |  | RENAL CANCER RECURRENT | 3 |
|  |  | RENAL CANCER STAGE III | 1 |
|  |  | RENAL CANCER STAGE IV | 1 |
|  |  | RENAL CELL CARCINOMA | 6 |
|  |  | RENAL CELL CARCINOMA STAGE III | 1 |
|  |  | RENAL CELL CARCINOMA STAGE IV | 2 |
|  |  | RENAL CYST | 12 |
|  |  | RENAL HAEMANGIOMA | 1 |
|  |  | RENAL NEOPLASM | 3 |
|  | Renal obstructive disorders | HYDRONEPHROSIS | 42 |
|  |  | PELVI-URETERIC OBSTRUCTION | 1 |
|  | Renal structural abnormalities and trauma | KIDNEY MALFORMATION | 1 |
|  |  | RENAL ATROPHY | 1 |
|  |  | RENAL HYPERTROPHY | 1 |
|  | Renal vascular and ischaemic conditions | GLOMERULAR VASCULAR DISORDER | 1 |
|  |  | NEPHROANGIOSCLEROSIS | 2 |
|  |  | RENAL ANEURYSM | 1 |
|  |  | RENAL ARTERIOSCLEROSIS | 1 |
|  |  | RENAL ARTERY STENOSIS | 3 |
|  |  | RENAL INFARCT | 6 |
|  |  | RENAL ISCHAEMIA | 1 |
|  |  | RENAL NECROSIS | 1 |
|  |  | RENAL TUBULAR INJURY | 1 |
|  |  | RENAL TUBULAR NECROSIS | 17 |
|  |  | RENAL VASCULAR THROMBOSIS | 1 |
|  |  | RENAL VEIN THROMBOSIS | 2 |
|  |  | RENAL VESSEL DISORDER | 1 |
|  |  | THROMBOTIC MICROANGIOPATHY | 11 |
| Ureteric disorders | Ureteric disorders NEC | RETROPERITONEAL FIBROSIS | 5 |
|  |  | URETERAL DISORDER | 1 |
|  |  | URETERAL NEOPLASM | 1 |
|  |  | URETERIC CANCER | 1 |
|  |  | URETERIC COMPRESSION | 1 |
|  |  | URETERIC OBSTRUCTION | 2 |
|  |  | URETERIC STENOSIS | 3 |
| Urethral disorders (excl calculi) | Urethral infections and inflammations | URETHRITIS | 2 |
|  |  | URETHRITIS NONINFECTIVE | 3 |
|  | Urethral neoplasms | TRANSITIONAL CELL CARCINOMA URETHRA | 1 |
| Urinary tract signs and symptoms | Bladder and urethral symptoms | BLADDER DISCOMFORT | 1 |
|  |  | BLADDER IRRITATION | 1 |
|  |  | BLADDER PAIN | 3 |
|  |  | BLADDER SPASM | 1 |
|  |  | DYSURIA | 41 |
|  |  | ENURESIS | 1 |
|  |  | INCONTINENCE | 20 |
|  |  | MICTURITION DISORDER | 1 |
|  |  | MICTURITION URGENCY | 10 |
|  |  | POLLAKIURIA | 42 |
|  |  | URETHRAL PAIN | 5 |
|  |  | URINARY INCONTINENCE | 29 |
|  |  | URINARY RETENTION | 90 |
|  | Urinary abnormalities | BACTERIURIA | 2 |
|  |  | CHOLURIA | 1 |
|  |  | CHROMATURIA | 69 |
|  |  | GLYCOSURIA | 1 |
|  |  | HAEMATURIA | 115 |
|  |  | LEUKOCYTURIA | 4 |
|  |  | PROTEINURIA | 53 |
|  |  | PYURIA | 5 |
|  |  | STERILE PYURIA | 2 |
|  |  | URINE ABNORMALITY | 6 |
|  |  | URINE ODOUR ABNORMAL | 6 |
|  | Urinary tract signs and symptoms NEC | FLANK PAIN | 34 |
|  |  | HAEMORRHAGE URINARY TRACT | 3 |
|  |  | NOCTURIA | 8 |
|  |  | PELVIC PAIN | 6 |
|  |  | POLYURIA | 14 |
|  |  | RENAL COLIC | 3 |
|  |  | RENAL PAIN | 6 |
|  |  | URINARY TRACT PAIN | 1 |
| Urolithiases | Renal lithiasis | NEPHROCALCINOSIS | 1 |
|  |  | NEPHROLITHIASIS | 21 |
|  | Urinary tract lithiasis (excl renal) | CALCULUS URINARY | 2 |
|  |  | URETEROLITHIASIS | 2 |

# Table S2 Standard drug name and original ICIs drug name in FAERS database

| **Standard drug name** | **Drug name in FARES database** |
| --- | --- |
| Nivolumab | "NIVOLUMAB"、 "OPDIVO"、 "Nivolumab"、 "BLINDED NIVOLUMAB"、 "NIVOLUMAB BMS"、 "nivolumab"、 "OPDYTA"、 "BMS-936558"、 "NIVOLUMAB 3MG/KG"、 "NIVOLUMAB (VMS-936558)"、 "NIVOLUMAB 100 MG BMS"、 "NIVOLUMAB 3 MG/KG"、 "NIVOLUMAB 3MG/KG (CAPPED A"、 "NIVOLUMAB, 240MG"、 "BMS?936558"、 "NIVOLUMAB, 240 MG"、 "NIVOLUMAB 100MG"、 "NIVOLUMAB 10MG/ML, 40 AND 100ML VIALS USE D"、 "NIVOLUMAB 240MG"、 "NIVOLUMAB 40MG"、 "NIVOLUMAB, 3 MG/KG"、 "Nivolumab BMS"、 "BLINDED Nivolumab"、 "NIVOLUMAB BMS"、 "NIVOLUMAB 100MG VIALS"、 "NIVOLUMAB 100MG/10ML BMS"、 "NIVOLUMAB 10MG/ML, 40 AND"、 "NIVOLUMAB 240 MG"、 "NIVOLUMAB 2MG/KG"、 "NIVOLUMAB 3 MG/KG ? CONTRO"、 "NIVOLUMAB 3 mg/kg"、 "NIVOLUMAB 40MG VIALS"、 "NIVOLUMAB BRISTOL-MYERS SQUIBB"、 "NIVOLUMAB UNK"、 "NIVOLUMAB, 100MG"、 "Nivolumab 240MG"、 "Nivolumab bms"、 "Nivolumab unk"; |
| Pembrolizumab | "KEYTRUDA"、 "PEMBROLIZUMAB."、 "MK-3475"、 "Pembrolizumab (MK-3475)"、 "PEMBROLIZUMAB 200MG"、 "INV?PEMBROLIZUMAB"、 "PEMBROLIXUMAB"、 "MK?3475"、 "BLINDED PEMBROLIZUMAB"、 "MK 3475"、 "Pembrolizumab (MK?3475)"、 "PEMBROLIZUMAB 200 MG"、 "PEMBROLIZUMAB 200MG MERCK RESEARCH LAB"、 "PEMBROLIZUMAB, 100MG/4ML MERCK"、 "COMPARATOR MK-3475"、 "PEMBROLIZUMAB, 2 MG/KG"、 "PEMBROLIZUMAB, 25 MG/ML"、 "Pembrolizumab 200mg"、 "COMPARATOR MK?3475"、 "INVESTIGATIONAL PEMBROLIZU"、 "PEMBROLIZUMAB 100MG/4ML MERCK"、 "COMPARATOR PEMBROLIZUMAB"、 "MK-3475 (PEMBROLIZUMAB) 200MG IV"、 "MK-3475 PEMBROLIZUMAB 100M"、 "MK-3475 Pembrolizumab 100m"、 "MK-3475(PEMBROLIZUMAB)"、 "PEMBRO"、 "PEMBROLIZUMAB 200 MG"、 "PEMBROLIZUMAB 100MG"、 "PEMBROLIZUMAB 100MG/4ML VI"、 "PEMBROLIZUMAB 120 MG"、 "PEMBROLIZUMAB 200MG MERCK RESEA"、 "PEMBROLIZUMAB ? 100MG LIQU"、 "PEMBROLIZUMAB LAST DOSE 6/9/17"、 "PEMBROLIZUMAB MERCK"、 "PEMBROLIZUMAB, 100 MG/ 4ML"、 "PEMBROLIZUMAB, 100 MG/ 4ML MERC"、 "PEMBROLIZUMAB, 100MG/4ML"、 "PEMBROLIZUMAB, 200 MG Q3W"、 "PEMBROLIZUMAB, 200 MG Q3W MERCK"、 "PEMBROLUZIMAB"、 "PEMBROLZUMAB 100 MG"、 "PEMROLIZUMAB"、 "Pembrolizumab (Mk-3475)"、 "Pembrolizumab 200 mg"、 "Pembroluzimab"; |
| Cemiplimab | "CEMIPLIMAB"、 "LIBTAYO"、 "REGN2810"、 "SAR439684"; |
| Atezolizumab | "ATEZOLIZUMAB."、 "Atezolizumab"、 "BLINDED Atezolizumab"、 "ATEZOLIZUMAB"、 "atezolizumab"、 "TECENTRIQ"、 "MPDL3280A (Atezolizumab)"、 "atezolizumab"、 "MPDL 3280A"、 "ALEZOLIZUMAB"、 "ATEZOLIZUMAB 1200MG"、 "ATEZOLIZUMAB ATEZOLIZUMAB"、 "BLINDED ATEZOLIZUMAB"、 "Blinded Atezolizumab"; |
| Durvalumab | "IMFINZI"、 "MEDI4736"、 "DURVALUMAB."、 "MEDI4736 (durvalumab)"、 "MEDI4736 (DURVALUMAB)"、 "MEDI4736 (Durvalumab)"、 "DURVALUMAB"、 "MEDI 4736"、 "INV-D URVALUMAB (MEDI4736)"、 "Medi4736"、 "MEDI4736(DURVALUMAB)"、 "Durvalumab"、 "INV?DURVALUMAB (MEDI4736)"、 "MEDI-4736"、 "DURVALUMAB 1,120MG"、 "MEDI4736 (MEDI4736)"、 "MEDI4736 10MG/KG"、 "PD?L1 inhibitor durvalumab"; |
| Avelumab | "AVELUMAB"、 "BAVENCIO"、 "MSB0010718C"、 "Avelumab"、 "AVELUMAB 200ML"、 "BLINDED AVELUMAB"、 "BLINDED MSB0010718C"、 "MSB0010718C;PLACEBO"、 "MSB0010718C; PLACEBO"、 "AVELUMAB (PFIZER)"、 "AVELUMAB/ PLACEBO"、 "AVELUMAB/PLACEBO"、 "AVELUMAB; PLACEBO"、 "MSB0010718C/PLACEBO"、 "MSB0010718C;PLACEBO (CODE"、 "MSB0010718C;PLACEBO (CODE NOT BROKEN) (AVELUMAB"、 "MSB0010718C;PLACEBO (Code"、 "MSB0010718C;PLACEBO/AVELUMAB"、 "MSB0010718C?AVELUMAB?250 M"; |
| Ipilimumab | "IPILIMUMAB"、 "YERVOY"、 "BLINDED IPILIMUMAB"、 "Ipilimumab"、 "IPILIMUMAB (BMS-734016; MDX-010 TRANSFECTOMA-DE"、 "ipilimumab"、 "IPILIMUMAB 1MG/KG"、 "IPILIMUMAB (BMS-734016; MDX-010"、 "IPILIMUMAB (BMS-7340106; MDX-010 TRANSFECTOMA-D"、 "IPILIMUMAB 50 MG VIALS"、 "IPILMUMAB"、 "IPILIMUMAB 50MG"、 "IPILIMUMAB 1 MG/KG"、 "IPILUMUMAB"、 "IPILUMUMAB 1MG/KG"、 "ipilimumab (BMS-734016; MDX-010 TRANSFECTOMA-DE"、 "IPILIMUMAB (BMS-734016; MDX-010 TRANSFERTOMADER"、 "IPILIMUMAB (BMS-734016;MDX-010"、 "IPILMUMAB 1MG/KG"、 "IPILIMUMAB (BMS-734016; MD"、 "BMS-734016"、 "BMS?734016"、 "IPILIBUMAB"、 "IPILIMUMAB (BMS-734016, MDX-010"、 "IPILIMUMAB 50 MG BMS"、 "IPILIMUMAB ADMINISTERED BY MSK"、 "IPILIMUMAB BRISTOL-MEYERS SQUIB"、 "IPILIMUMAB BRISTOL-MYERS SQUIBB"、 "IPILIMUMAB N3 I1"、 "IPLILMUNAB 1MG/KG"、 "IPLIMUMAB"、 "Ipilimumab (BMS-734016; MDX-010"、 "Iplimumab"、 "ipilumumab"; |
| Tremelimumab | "TREMELIMUMAB."、 "TREMELIMUMAB 10 MG/KG"、 "TREMELIMUMAB"、 "TICILIMUMAB"、 "TREMELIBUMAB"、 "TREMELIMUMAB, 10 MG/KG"、 "TREMELIMUMAB CODE NOT"; |

# Table S3 Standard drug name and original ICIs chemotherapy drug name in FAERS database

| **Standard drug name** | **Original drug name in FARES database** |
| --- | --- |
| Cisplatin | "CISPLATIN."、 "CISPLATINE"、 "CISPLATIN (Manufacturer unknown)"、 "CISPLATIN (Manufacturer unknown"、 "CISPLATINUM"、 "PLATINOL"、 "CISPLAN"、 "CISPLATIN ACCORD"、 "CISPLATINE ACCORD"、 "CISPLATIN (Manufacturer un"、 "BRIPLATIN"、 "Cisplatin Accord"、 "Cisplatin (Unknown)"、 "CISPLATINE MYLAN"、 "CISPLATIN INJECTION"、 "CDDP"、 "RANDA"、 "CISPLATIN MYLAN"、 "Cisplatin Mylan"、 "Cisplatine"、 "Cisplatine Teva"、 "PLATOSIN"、 "CIS-PLATINUM"、 "CISPLATINE TEVA"、 "FAULDCISPLA"、 "CISPLATIN TEVA"、 "CISPLATIN (119875)"、 "CISPLATIN KABI"、 "Cisplatina"、 "CISPLATINA"、 "Cisplatin Teva"、 "cisplatin Accord"、 "CISPLATINO"、 "PLATIDAM 0.5 MG/MG CONCENT"、 "NUO XIN"、 "CISPATIN"、 "CISPLATINO TEVA - TEVA PHARMA B"、 "CISPLATIN PLIVA"、 "CISPLATINO ACCORD"、 "CISPLATINO ACCORD HEALTHCA"、 "Cisplatin accord"、 "IA-CALL"、 "NEOPLATIN"、 "CISPLATINE ACCORD 1 mg/ml,"、 "CISPLATINO (644A)"、 "CISPLATINO TEVA ITALIA - 0.5 MG"、 "Neoplatin"、 "cisplatine"、 "CISPLATIN EBEWE"、 "CISPLATIN NC"、 "CISPLATINO SANDOZ - 1 MG/ML CO"、 "cisplatin accord healthcare"、 "CISPLATIN FOR INJECTION (FREEZE-DRIED)"、 "CISPLATIN SANDOZ"、 "CISPLATIN-TEVA 1 MG/ML KONZENTRAT ZUR HERSTELLU"、 "CISPLATINA ACCORD"、 "CISPLATINO ACCORD HEALTHCARE IT"、 "CISPLATINO TEVA ITALIA"、 "COMPARATOR CISPLATIN"、 "Cisplastin"、 "IACALL"、 "CISPLATIN MARUKO"、 "CISPLATIN TEVA 1 MG/ML KONZENTRAT ZUR HERSTELLU"、 "CISPLATINE ACCORD 1 MG/ML,"、 "CISPLATINE TEVA 1 mg/1 ml"、 "CISPLATINO PFIZER"、 "CISPLATINO TEVA"、 "CISPLATINO TEVA ITALIA - 1MG/ML"、 "CISPLATINO TEVA ITALIA 1 M"、 "CISplatin INJECTION"、 "Cis-platinum"、 "Cisplatina Teva"、 "IA CALL"、 "KEMOPLAT"、 "Nuoxin"、 "cisplatin accord"、 "Accord Cisplatin"、 "CISPLATIN ACCORD HEALTHCAR"、 "CISPLATIN SOLUTION"、 "CISPLATIN Teva"、 "CISPLATINE ACCORD 1 mg/ml"、 "CISPLATINE MERCK"、 "CISPLATINE MYLAN 10 MG/10 ML, SOLUTION ? DILUER POUR PERFUSION"、 "CISPLATINE TEVA 10 MG/10 ML"、 "CISPLATYL"、 "Cisplatin Pfizer"、 "Cisplatine Accord"、 "Cisplatine Mylan"、 "Cisplatino"、 "Cisplatyl"、 "DDP"、 "EBEWE ARZNEIM CISPLATIN"、 "Fauldcispla"、 "IA COR"、 "IA?CALL"、 "Platosin"、 "Randa"、 "ABIPLATIN"、 "Accord cisplatin"、 "CIPLATINE"、 "CIPLATINO (CISPLATIN)"、 "CIS-PLATINA"、 "CIS?PLATINUM"、 "CISPALTIN ACCORD"、 "CISPLASTIN"、 "CISPLATIN ACCORD"、 "CISPLATIN (CDDP)"、 "CISPLATIN (DBL)"、 "CISPLATIN 100MG/100ML MDV"、 "CISPLATIN 153MG CYCLE 4"、 "CISPLATIN 1MG"、 "CISPLATIN 200MG FRESENIUS"、 "CISPLATIN ACCORD HEALTHCARE"、 "CISPLATIN Accord"、 "CISPLATIN CISPLATIN"、 "CISPLATIN DBL"、 "CISPLATIN EBEWE ARZNEIM"、 "CISPLATIN INJ 50MG/50ML BE"、 "CISPLATIN INJECTION MYLAN"、 "CISPLATIN INJECTION, BP SINGLE DOSE VIALS. 10MG"、 "CISPLATIN PHARMACHEMIE"、 "CISPLATIN TEVA 1MG/ML"、 "CISPLATIN ^HOSPIRA^"、 "CISPLATIN ^PFIZER^"、 "CISPLATIN-AQ"、 "CISPLATIN/HOSPIRA"、 "CISPLATINA TEVA"、 "CISPLATINE ACCORD 1 MG/ML"、 "CISPLATINE MYLAN 1 MG/ML,"、 "CISPLATINE TEVA 1 mg/ml"、 "CISPLATINE TEVA 100 MG/100 ML"、 "CISPLATINE TEVA 50 MG/ 50 ML"、 "CISPLATINO ACCORD 1 MG /ML CONCENTRATE FOR SOLU"、 "CISPLATINO ACCORD HEALTHCARE ITALIA 1 MG/ML CONCENTRATO PER SOLUZIONE"、 "CISPLATINO ACCORD1 MG/MLCO"、 "CISPLATINO EBEWE"、 "CISPLATINO PHARMACIA"、 "CISPLATINO PHARMACIA 1 MG/"、 "CISPLATINO TEVA ITALIA -1MG/ML CONCENTRATO PER"、 "CISPLATINO TEVA ITALIA 0,5 MG/ML CONCENTRATO PER SOLUZIONE PER INFUSIO"、 "CISPLATINUM ACCORD"、 "CYSPLATIN"、 "CYTOPLATIN-50 AQUEOUS"、 "Cisplatin (Hospira)"、 "Cisplatin Injection"、 "Cisplatin Injection Mylan"、 "Cisplatin Teva 1 mg/ml Konzentrat"、 "Cisplatin accord healthcare"、 "Cisplatin mylan"、 "Cisplatin teva"、 "Cisplatin-Teva"、 "Cisplatine Pfizer"、 "Cisplatinum"、 "Cisplatinum Accord"、 "DIFPLATIN"、 "ESHAP (CISPLATIN)"、 "IP CISPLATIN"、 "NUOXIN"、 "Nuo Xin"、 "PLATIDIAM"、 "PLATIN"、 "PLATINE"、 "PLATINEX"、 "PLATOSIN 1MG/ML"、 "SINPLATIN"、 "Sinplatin"、 "[PSS GPN] CISPLATIN DBL"、 "cis-platinum"、 "cisplastin"、 "cisplatin ACCORD"、 "cisplatin Mylan"、 "cisplatin Teva"、 "cisplatin injection"、 "cisplatine Mylan"、 "cisplatine accord"、 |
| Carboplatin | "CARBOPLATIN."、 "PARAPLATIN"、 "CARBOPLATINE"、 "Carboplatine Hospira"、 "CARBOPLATIN (Manufacturer"、 "CARBOPLATIN (Manufacturer Unknown)"、 "CARBOPLATIN HOSPIRA"、 "CARBOPLATIN ACCORD"、 "CARBOPLATIN (Manufacturer Unkno"、 "Carboplatin (Unknown)"、 "Carboplatin MYLAN"、 "CARBOPLATIN INJECTION 10mg/ml"、 "CARBOPLATINE ACCORD"、 "Carboplatin Accord"、 "CARBOPLATINUM"、 "CARBOPLATINO TEVA 10 MG/ML"、 "CARBOPLATINO"、 "Carboplatin Inj. 50mg ^NK^"、 "CARBOPLATINE HOSPIRA"、 "CARBOPLATIN TEVA"、 "CARBOPLATINO TEVA - 10 MG/ML -"、 "Carboplatin (Watson Laboratorie"、 "Carboplatin Hospira"、 "Carboplatine"、 "CARBOPLATIN INJECTION 10mg"、 "CARBOPLATINO TEVA - TEVA PHARMA"、 "Carboplatin Pfizer"、 "CARBOPLATIN ACTAVIS"、 "Carboplatino Pfizer Italia"、 "CARBOPLATINO TEVA"、 "CARBOPLATIN (241240)"、 "CARBOPLATINE TEVA"、 "FAULDCARBO"、 "Novoplatinum"、 "CARBOPLATIN INTRAVENOUS INFUSION"、 "CARBOPLATINO (2323A)"、 "CARBOPLATIN INTRAVENOUS INFUSIO"、 "CARBOPLATINO AHCL"、 "CARBOPLATIN INTRAVENOUS IN"、 "CARBOPLATINA HIKMA"、 "CARBOPLATINA ACCORD"、 "Carboplatin Concentrate for solution for infusion"、 "CARBOPLATINE TEVA 10 mg/ml"、 "CARBOPLATINO TEVA - 600 MG/60 M"、 "CARBOPLATINE KABI"、 "CARBOMEDAC"、 "Carboplatin Concentrate fo"、 "carboplatine"、 "Carboplasin"、 "CARBOPLAT"、 "CARBOPLATIN 10MG/ML"、 "RIBOCARBO-L"、 "CARBOMERCK"、 "CARBOPLATINA"、 "Carbomedac"、 "Carboplatin accord"、 "CARBOPLAN"、 "CARBOPLATIN PFIZER"、 "CARBOPLATINO TEVA - 10 MG/ML - TEVA PHARMA B.V."、 "Carboplatin Actavis"、 "Carboplatin teva"、 "KARBOTEEN"、 "Bopacatin 10 MG/ML"、 "CARBOPLATIN/HOSPIRA"、 "CBDCA"、 "Carboplatin Teva"、 "BLINDED CARBOPLATIN"、 "BOPACATIN 10 MG/ML"、 "BOPACATIN 10 MG/ML CONCENTRATE FOR SOLUTION FOR"、 "CARBOPLATINO TEVA - 600 MG/60 ML - TEVA PHARMA"、 "CARBOSIN 450 MG"、 "CARMEN"、 "Carbosin"、 "carboplatin ahcl"、 "CARBOPLATIN 10 MG/ML 60 ML"、 "CARBOPLATIN AUC 2"、 "CARBOPLATIN HEXAL"、 "CARBOPLATIN KABI"、 "CARBOPLATIN MYLAN"、 "CARBOPLATIN Pfizer"、 "CARBOSIN 150MG, 10MG/ML"、 "Carboplatin Ahcl"、 "Carboplatino Teva"、 "Carboplatinum"、 "CARBOPLATIN CONCENTRATE FO"、 "CARBOPLATIN PLIVA"、 "CARBOPLATIN, 600MG HOSPIRA"、 "CARBOPLATINE INFUUS"、 "CARBOPLATINE TEVA 10 mg/ml, solution pour perfusion"、 "CARBOPLATINO TEVA - TEVA PHARMA B.V"、 "CARBOTINOL"、 "Carboplatin Inj. 150mg ^NK"、 "Carboplatin Inj. 150mg ^NK^"、 "CARBOPLATIN 10 MG/ML 45 ML"、 "CARBOPLATIN 10MG/ML 60 ML"、 "CARBOPLATIN 10MG/ML SOLUTI"、 "CARBOPLATIN AHCL"、 "CARBOPLATIN FOR INJECTION"、 "CARBOPLATIN INJ 600MG/60ML"、 "CARBOPLATIN OMNICARE"、 "CARBOPLATIN PLIVA 50"、 "CARBOPLATINE ARROW"、 "CARBOPLATINO AHCL - ACCORD HEALTHCARE LIMITED"、 "CARBOPLATINO TEVA - 10 MG/"、 "CARBOPLATINO TEVA - 15 ML 10 MG"、 "CARBOPLATINO TEVA - TEVA PHARMA B.V."、 "CARBOPLATINO TEVA 10 MG/ML CONC"、 "CARBOPLATINO TEVA 10 mg/ml"、 "CARBOPLATINO TEVA 600 MG/60 ML"、 "CARBOPLATINO TEVA ? 10 MG/"、 "CARBOSIN"、 "CYCLOPLATIN"、 "Carboplatin Injection"、 "Carboplatin pfizer"、 "Carboplatin-Actavis 10 mg/ml concentrate for so"、 "Carboplatine Pfizer"、 "Carboplatino Hospira"、 "DBL CARBOPLATIN"、 "Tevacarbo"、 "carboplatin Accord"、 "BOPACATIN"、 "CARBO /00740901/"、 "CARBOPA"、 "CARBOPLATIN AUC 5 IV"、 "CARBOPLATIN CONCENTRATE FOR SOLUTION FOR INFUSI"、 "CARBOPLATIN HIKMA"、 "CARBOPLATIN INJECTION BP"、 "CARBOPLATIN SANDOZ"、 "CARBOPLATIN ^FRESENIUS KAB"、 "CARBOPLATIN, 600MG/60ML"、 "CARBOPLATINE ACCORD 10 mg/ml, solution pour perfusion"、 "CARBOPLATINE HOSPIRA 10 mg"、 "CARBOPLATINE SUN"、 "CARBOPLATINE TEVA 10 MG/ML"、 "CARBOPLATINO AHCL 10 MG/ML"、 "CARBOPLATINO PFIZER - 50MG/5ML"、 "CARBOPLATINO TEVA - 1 FLACONE 600 MG/60 ML - TE"、 "CARBOPLATINO TEVA - 45 ML 10 MG/ML - TEVA PHARM"、 "CARBOPLATINO TEVA - 5 ML 10 MG/"、 "COMPARATOR CARBOPLATIN"、 "Carboplatin (Watson Laboratories)"、 "Carboplatin Mylan"、 "Carboplatin solution for i"、 "Carboplatin-TEVA"、 "Carboplatino"、 "ACCORD CARBOPLATIN"、 "Actoplatin"、 "BO BEI"、 "BOBEI"、 "BOPACATIN 10 MG / ML"、 "Bopacatin"、 "CABOPLATINE SUNPHARMA 10 MG/ML"、 "CABOPLATINE SUNPHARMA 10 mg/ml"、 "CARBO"、 "CARBO (CARBOPLATIN)"、 "CARBO /00740901/"、 "CARBOLPLATIN"、 "CARBOPALTIN"、 "CARBOPALTIN AHCL"、 "CARBOPLAIN AUC 2"、 "CARBOPLASIN"、 "CARBOPLATEN"、 "CARBOPLATIN (241240) 3600"、 "CARBOPLATIN (AUC5)"、 "CARBOPLATIN (BLINDED)"、 "CARBOPLATIN (Blinded)"、 "CARBOPLATIN (CBDCA)"、 "CARBOPLATIN (DBL)"、 "CARBOPLATIN (GENERIC)"、 "CARBOPLATIN - CONCENTRATE"、 "CARBOPLATIN - POWDER FOR S"、 "CARBOPLATIN 10MG/ML 60ML VIAL TEVA"、 "CARBOPLATIN 450MG/45ML SDV"、 "CARBOPLATIN 450MG/45ML VIAL HOSPIRA"、 "CARBOPLATIN 50MG"、 "CARBOPLATIN 600MG"、 "CARBOPLATIN 600MG TEVA"、 "CARBOPLATIN ACCORD HEALTHCARE"、 "CARBOPLATIN ACOORD"、 "CARBOPLATIN ACTAVIS 10 MG/"、 "CARBOPLATIN ARROW"、 "CARBOPLATIN ARROW 10MG/ML"、 "CARBOPLATIN ARROW 10MG/ML SOLUTION FOR INFUSION"、 "CARBOPLATIN AUC2"、 "CARBOPLATIN AUC5"、 "CARBOPLATIN AUROBINDO CONC"、 "CARBOPLATIN AUROBINDO CONCENTRATE"、 "CARBOPLATIN Accord"、 "CARBOPLATIN CARBOPLATIN"、 "CARBOPLATIN CONCENTRATE 10"、 "CARBOPLATIN EBEWE"、 "CARBOPLATIN FOR INJECTION,"、 "CARBOPLATIN INFUUS"、 "CARBOPLATIN INJ 150 / 15ML"、 "CARBOPLATIN INJ 450MG/45ML"、 "CARBOPLATIN INJ 50MG/5ML"、 "CARBOPLATIN INJ. 150MG ^NK^"、 "CARBOPLATIN INJ. 50MG ^NK^"、 "CARBOPLATIN KOCAK"、 "CARBOPLATIN MEDAC"、 "CARBOPLATIN OMNICARE 10 MG"、 "CARBOPLATIN PLIVA 10MG/ML"、 "CARBOPLATIN PLIVA KONCENTRAT ZA"、 "CARBOPLATIN SAGENT"、 "CARBOPLATIN TEVA PHARMA B.V."、 "CARBOPLATIN ^ACTAVIS^"、 "CARBOPLATIN ^NK^"、 "CARBOPLATIN, 450MG"、 "CARBOPLATIN-GRY 600 MG/60 ML"、 "CARBOPLATIN-TEVA"、 "CARBOPLATIN-TEVA 450 MG"、 "CARBOPLATINE ACCORD 10 MG/"、 "CARBOPLATINE ACCORD 10 mg/"、 "CARBOPLATINE ARROW 10 MG/M"、 "CARBOPLATINE ARROW 10 MG/ML"、 "CARBOPLATINE HOSPIRA 10 MG/ML SOLUTION INJECTAB"、 "CARBOPLATINE INFOPL CONC 10MG/M"、 "CARBOPLATINE INFOPL CONC 10MG/ML"、 "CARBOPLATINE KABI 10 mg/ml"、 "CARBOPLATINE PFIZER"、 "CARBOPLATINE WINTHROP"、 "CARBOPLATINE?ACCORD"、 "CARBOPLATINO 460 MG"、 "CARBOPLATINO ACCORD"、 "CARBOPLATINO AHCL 10 MG/ML CONCENTRATO PER SOLUZIONE PER INFUSIONE"、 "CARBOPLATINO FERRER FARMA"、 "CARBOPLATINO PHARMACIA"、 "CARBOPLATINO TEVA - 15 ML 10 MG/ML - TEVA PHARM"、 "CARBOPLATINO TEVA - IV 45 ML 10 MG/ML - TEVA PH"、 "CARBOPLATINO TEVA - IV 5 ML 10"、 "CARBOPLATINO TEVA 10 MG/ML CONCENTRADO PARA SOLUCION PARA PERFUSION ,"、 "CARBOPLATINO TEVA ? 15 ML"、 "CARMEN /00740901/"、 "CARMEN (CARBOPLATIN)"、 "CARMEN /00740901/"、 "CRABOPLATIN HOSPIRA"、 "Carboplatin 10mg/ml"、 "Carboplatin Arrow 10mg/ml solution for infusion"、 "Carboplatin Pliva"、 "Carboplatin Pliva 50"、 "Carboplatin SUN 10 mg/ml concen"、 "Carboplatin ahcl"、 "Carboplatin hospira"、 "Carboplatin injection"、 "Carboplatine Accord"、 "Carboplatine kabi"、 "Cytocarb"、 "DBL Carboplatin"、 "Fauldcarbo"、 "KEMOCARB"、 "PREVIOUS CHEMOTHERAPY WITH CARBOPLATIN"、 "carboplatin Ahcl"、 "carboplatin Hospira"、 "carboplatin Teva"、 "carboplatin accord"、 "carboplatino"、 "carboplatinum"、 |
| Nedaplatin | "NEDAPLATIN"、 "Nedaplatin"、 "AQUPLA"、 "LUBEI (NEDAPLATIN)"、 "JIE BAI SHU"、 "nedaplatin"、 |
| Dicycloplatin | "BLINDED PLACEBO"、 "BLINDED NO TREATMENT RECEIVED"、 "BLINDED Placebo"、 "BLINDED THERAPY"、 "INVESTIGATIONAL DRUG"、 "BLINDED NO TREATMENT RECEI"、 "BLINDED *Placebo"、 "STUDY PROCEDURE"、 "PDR001"、 "BLINDED NO STUDY DRUG GIVE"、 "MK-0000"、 "INCB039110"、 "RO 4929097 (GAMMA SECRETASE INH"、 "BMS-986016-01"、 "BLINDED PF-06425090"、 "TG4010"、 "QVA149"、 "NKTR-214"、 "MK-8908"、 "UTD1 (EPOTHILONE ANALOG)"、 "AZD1775"、 "VX-661/VX-770"、 "MK?0000"、 "BMS-986205-04"、 "PQR309"、 "AZD9150"、 "CC-122"、 "BLINDED PF-06439535"、 "CRLX101"、 "INVESTIGATIONAL ANTINEOPLASTIC DRUGS"、 "AZD2014"、 "IPI-549"、 "VX-770/VX-661"、 "NKTR?214"、 "XL888 (HSP90 INHIBITOR)"、 "BLINDED ASP0113"、 "BLINDED PF-06290510"、 "BMS?986016?01"、 "IG.AD.MLPI.TK"、 "*JNJ-56022473"、 "ABT-751"、 "ACY-241"、 "CD4+/EGFRT+"、 "CD8+/EGFRT+"、 "HU5F9-G4"、 "ASP0113"、 "CRS-207"、 "POL6326"、 "APX005M"、 "RO 6895882 (CEA-IL2V)"、 "RO 6958688 (T-CELL BISPECI"、 "AZD1775 ASTRAZENECA"、 "GVAX"、 "INC280"、 "ABT-414"、 "ABT-494"、 "Investigational drug"、 "JNJ-56022473"、 "AZD5069"、 "BIIB023"、 "CT-P13 (REMICADE BIOSIMILAR)"、 "MOR03087"、 "PF-04518600"、 "TRU-016 (ANTI-CD37 MAB)"、 "X4P-001"、 "BLINDED NO STUDY DRUG GIVEN"、 "CC-292"、 "CMB305"、 "INVESTIGATIONAL ANTINEOPLASTIC"、 "LSZ102"、 "MK-0000 (111)"、 "PF-05082566"、 "ADXS11-001"、 "AZD1775 ASTRA ZENECA"、 "B-701 25MG/KG"、 "BLINDED CSJ148"、 "FT-2102"、 "MBG453"、 "MM-302"、 "RECOMBINANT FC FUSION PROTEIN ANGIOGENESIS INHI"、 "64CU-MM-302"、 "AZD6738"、 "HDM201"、 "HM95573 (RAF KINASE INHIBITOR)"、 "LGH447"、 "MK-2206"、 "MOXR0916"、 "MPDL3280A"、 "QGE031"、 "RO 7009789 (CD40 AGONIST)"、 "SD-101"、 "SRA737"、 "TAS-118"、 "VX-659"、 "ABL001"、 "AZD5363"、 "BMS-986156-01"、 "CPI-613"、 "ETBX-011"、 "GS-4059"、 "INCB052793"、 "LY3022855"、 "MEDI3617"、 "PF-04691502"、 "PF-06439535"、 "PF?04518600"、 "hESC-RPE"、 "ASP015K"、 "BLINDED placebo"、 "KRP203"、 "PF-06747775"、 "BGB-3111"、 "BI 836845"、 "BLINDED PDR001"、 "CC?122"、 "CPI-444 (ADENOSINE-A2A RECEPTOR ANTAGONIST)"、 "CSJ148"、 "EGF816"、 "MTIG7192A"、 "PF-04136309"、 "PF-06425090"、 "RO 6958688 (T-CELL BISPECIFIC MONOCLONAL ANTIBO"、 "RO 7082859 (CD20/C3 T-CELL"、 "TAS-114"、 "TRC105"、 "ACE INHIBITOR (RO 31-2201)"、 "BLINDED BLINDED THERAPY"、 "BLINDED PF-06928316"、 "BMS-986178-01"、 "CTL119"、 "GDC-0919 (IDO1 INHIBITOR)"、 "GDC-0994 (ERK INHIBITOR)"、 "GS-5745"、 "GSK3684934"、 "HESC-RPE"、 "INCB050465"、 "INO-5401"、 "INO-9012"、 "PD-325,901"、 "ABT-165"、 "ALT-803"、 "AMG 232"、 "BLINDED PF?06425090"、 "BMS-734019"、 "BMS-986192-01"、 "CT-P13 (REMICADE BIOSIMILA"、 "HSC835"、 "IMG?7289"、 "ISA101 (HPV)"、 "MOR202"、 "NY-ESO-1"、 "PF?04691502"、 "PF?05082566"、 "RO6870810"、 "ASP0113 BLINDED"、 "AUTOLOGOUS DENDRITIC CELL VACCINE (NDV INFECTED TUMOR CELLS)"、 "AZD4547"、 "BLINDED LLG783"、 "BLINDED Placebo (SHAM TREATMENT"、 "CC-223"、 "CEP-41750"、 "CYTOKINE INDUCED KILLER CE"、 "DENDRITIC CELLS CYTOKINE INDUCED KILLER CELLS"、 "GAMMA-SECRETASE INHIBITOR (UNSP"、 "GSK2636771B"、 "HM95573 (RAF KINASE INHIBI"、 "IMO-2055"、 "INCB057643 (INC057643) TABLET"、 "JCAR015"、 "KHK2455"、 "LAM?002A"、 "LITERATURE - PHARMA"、 "MEDICAMENT EXPERIMENTAL"、 "MOR00208"、 "MOR208"、 "PF-00547659 (INVESTIGATIVE)"、 "PF-06410293;ADALIMUMAB"、 "REGN1979"、 "RO 6895882 (CEA?IL2V)"、 "RO 7082859 (CD20/C3 T-CELL BISPECIFIC MAB)"、 "STUDY"、 "STUDY DRUG"、 "TGR-1202"、 "U-47700"、 "VX-445"、 "X4P?001"、 "6-CHLORO-N-BUTYL-PHTHALIDE"、 "ABP 215"、 "ABT 165"、 "ALGENPANTUCEL?L"、 "AZD0156"、 "AZD2811"、 "AZD7762"、 "BB2121"、 "BG00011"、 "BLINDED GP2015"、 "BLINDED LHW090"、 "BLINDED No drug given"、 "BLINDED PLACEBO RUN IN"、 "BLINDED Placebo_Diskus"、 "BLINDED STUDY MEDICATION"、 "BLINDED Unknown"、 "BMS-986148-01"、 "BMS-986179-01"、 "BMS-986207-01"、 "BMS?986205?04"、 "Blinded Placebo"、 "CC-220"、 "CFZ533"、 "CHIMERIC ANTIGEN RECEPTOR (CAR)"、 "CPI-444 (ADENOSINE-A2A RECEPTOR"、 "Dicycloplatin"、 "F-06425090;PLACEBO"、 "FT?2102"、 "GSK 3359609"、 "HEAT SHOCK PROTEIN 90 INHIBITOR (UNSPECIFIED)"、 "HUMANIZED ANTI-GD2"、 "IMA901"、 "INCB040093"、 "INCB054329"、 "INCB054828"、 "INVESTIGATIONAL DRUG (UNSPECIFIED)"、 "LCL161"、 "Lifein"、 "MEDI7247"、 "MENINGOCOCCAL GROUP B RLP2086"、 "NY-ESO-1 TCR"、 "PARENTALLY-DERIVED NK CELLS"、 "PF-06290510"、 "PF-06290510-PLACEBO"、 "PF-06290510; PLACEBO"、 "PF-06425090/PLACEBO"、 "PF-06425090; PLACEBO"、 "PF-06647020"、 "PF?06425090"、 "PLX3397"、 "POL6326-07"、 "PROSTVAC-F/TRICOM"、 "PRRT (PEPTIDE RECEPTOR RADIONUCLIDE THERAPY)"、 "PT-112"、 "Placebo_Diskus"、 "RO 6958688 (T-CELL BISPECIFIC M"、 "RO 6958688 (T?CELL BISPECI"、 "STUDY MED NOT GIVEN (15786)"、 "T CELL RECEPTOR GENE THERA"、 "Vaccine peptide cocktail"、 "X-82 (MULTIKINASE VEGFR/PD"、 "blinded THERAPY"、 "3F8"、 "?HTX-011 VS PLACEBO VS CON"、 "AA 24 (INVESTIGATIONAL PRODUCT)"、 "ACP-319"、 "ACP?319"、 "ADP53-DC"、 "AH-7921"、 "AL-335"、 "APO-DC"、 "ARRY-520"、 "ASP8273"、 "AT-406"、 "Ad.p53-DC"、 "BGB?3111"、 "BGJ398"、 "BIIB059"、 "BLINDED BLINDED NO TREATME"、 "BLINDED CSJ137"、 "BLINDED GSK2330811"、 "BLINDED INTRAVITREAL SHAM"、 "BLINDED INVESTIGATIONAL MEDICIN"、 "BLINDED INVESTIGATIONAL MEDICINAL PRODUCT (IMP)"、 "BLINDED MHAA4549A (ANTI-INFLUEN"、 "BLINDED No Study Drug Administered"、 "BLINDED PF-04518600"、 "BLINDED PF-06886992"、 "BLINDED PF?06290510"、 "BLINDED PF?06439535"、 "BLINDED PLACEBO PH 3.7"、 "BLINDED PLACEBO PH 7.0"、 "BLINDED Placebo Solution f"、 "BLINDED STUDY DRUG"、 "BLINDED TMX-049"、 "BLINDED VACCINES (VACCINES"、 "BLINDED VEHICLE FOAM"、 "BMS-791325 (NS5B INHIBITOR)"、 "BMS-813160"、 "BMS?791325"、 "BMS?986178?01"、 "BNC105"、 "BNC105P"、 "BTCT4465A"、 "Blinded No Treatment Recei"、 "Blinded no treatment recei"、 "Blinded therapy"、 "Blinded trial medication"、 "CC-122 (PLEIOTROPIC PATHWAY MOD"、 "CC-90009"、 "CC?223"、 "CCT245737"、 "CD22-DIRECTED CAR THERAPY%"、 "CD4+/EGFRT"、 "CD4/EGFRT+"、 "CD8+/EDFRT+"、 "CD8/EGFRT+"、 "CIPEMASTAT"、 "CORD BLOOD-DERIVED NATURAL KILLER CELLS (CB-NK)"、 "CPP-115"、 "CRLX-101"、 "CRS?207"、 "CSJ137"、 "CT-P13"、 "CYL-02"、 "DCC-2618"、 "DICYCLOPLATIN"、 "DSTP3086S"、 "EPSTEIN BARR VIRUS SPECIFIC T-CELLS"、 "F50067"、 "G1T28 (G1T28)"、 "G305 (NY-ESO-1 RECOMBINANT PROTEIN PLUS GLA-SE)"、 "GAMMA-SECRETASE INHIBITOR (UNSPECIFIED)"、 "GP2013 (RITUXIMAB BIOSIMILAR)"、 "GS-9883IFTC!TAF"、 "GS?5745"、 "GSK2256098"、 "GSK2636771"、 "GSK3326595"、 "HF10 (INVESTIGATIONAL PROD"、 "HIV VACCINE"、 "HSP-130"、 "HUMANIZED ANTI-GD2 MAB"、 "HZ/SU + AS01B"、 "IMOXINE"、 "INC8050465"、 "INCAGN01876"、 "INCB 53914"、 "INCSHR01210 (INCSHR01210) Injec"、 "INVESTIGATIONAL DRUG (UNSPECIFI"、 "INVESTIGATIONAL VACCINE"、 "IPI?549"、 "JTX 2011"、 "KENGSH?EMYCINL"、 "LAG525"、 "LAM-002A"、 "LGH 447"、 "LV305"、 "LXS196"、 "LY2510924"、 "M9241 (NHS-IL12)"、 "MEDI0457"、 "MEDI0680"、 "MK 2206"、 "MK-0000 (325)"、 "MK-0000 (348)"、 "MK-1308"、 "MK-1775"、 "MK-4280"、 "MSC2490484A (DNA-PK INHIBITOR)"、 "MVA-BN-CV301"、 "MX2 (3'-DEAMINO-3'-MORPHOLINO-13-DEOXO-10-HYDRO"、 "MYL-1401O (TRASTUZUMAB BIOSIMIL"、 "OPB-111077"、 "P53MVA VACCINE"、 "PEGPH 20"、 "PEPTIDE PULSED DC VACCINE"、 "PF 04518600"、 "PF-00547659"、 "PF-04236921"、 "PF-04523655"、 "PF-05212377"、 "PF-06290510 OR PLACEBO"、 "PF-06290510 or Placebo"、 "PF-06290510;PLACEBO"、 "PF-06410293"、 "PF-06425090 (PLACEBO)"、 "PF-06425090;PLACEBO"、 "PF-06664178"、 "PF-06671008"、 "PF-06865571"、 "PF?06290510 or Placebo"、 "PF?06425090/PLACEBO"、 "PF?06425090/PLACEBO (BLIND"、 "PF?06747775"、 "PLACEBO (BLINDED)"、 "PLACEBO: BLINDED"、 "PSMA 617"、 "PSMA ADC"、 "PSMA-ADC"、 "RECOMBINANT VARICELLA ZOST"、 "RHIGM22 (MONOCLONAL ANTIBODIES)"、 "RHLGM22"、 "RO 7082859 (CD20/C3 T?CELL"、 "SEPHB4-HSA LAST DOSE 6/9/17"、 "STUDY MED. NOT GIVEN"、 "TAS 118"、 "TAS 120"、 "TAS-119"、 "TAS-120"、 "TBI-1301 (NY-ESO-1-SPECIFI"、 "TBI-1501"、 "TPI 287"、 "TPIV 200"、 "TRC105 (ANTI-ENDOGLIN MAB)"、 "TRU-016"、 "TUMOR NECROSIS FACTOR RECE"、 "UNKNOWN TRIAL MEDICATION"、 "UTIDELONE"、 "VACCINE PLACEBO"、 "VEGLIN"、 "WILMS TUMOR GENE 1 (WT1) P"、 "WT1 VACCINE"、 "blinded study medication"、 "blinded therapy"、 |
| Oxaliplatin | "OXALIPLATIN."、 "ELOXATIN"、 "ELPLAT"、 "OXALIPLATINE"、 "OXALIPLATIN (Manufacturer"、 "OXALIPLATIN (Manufacturer unknown)"、 "OXALIPLATIN ACCORD"、 "OXALIPLATINE ACCORD"、 "OXALIPLATIN (Manufacturer unkno"、 "OXALIPLATIN INJECTION 50MG"、 "Oxaliplatin Accord"、 "OXALIPLATIN 5mg/ml CONCENT"、 "OXALIPLATIN 5mg/ml CONCENTRATE FOR SOLUTION FOR"、 "Oxaliplatin (Unknown)"、 "Oxaliplatin Hospira"、 "OXALIPLATINE TEVA"、 "OXALIPLATIN HOSPIRA"、 "OXALIPLATINE HOSPIRA"、 "Oxaliplatino SUN 5 mg/ml,"、 "OXALIPLATIN 5mg/ml CONCENTRATE"、 "Oxaliplatin accord"、 "Oxaliplatino SUN 5 mg/ml, conce"、 "OXALIPLATINO"、 "OXALIPLATIN SANDOZ"、 "OXALIPLATIN TEVA"、 "OXALIPLATINO ACCORD"、 "OXALIPLATINO TEVA"、 "Tevaoxali"、 "OXALIPLATIN WINTHROP"、 "OXALIPLATINO TEVA - TEVA ITALIA S.R.L."、 "OXALIPLATINO TEVA 5 MG / M"、 "OXALIPLATINO 5 MG/ML CONCE"、 "OXALIPLATIN MYLAN"、 "Oxaliplatin Concentrate fo"、 "Oxaliplatin SUN 5 mg/ml co"、 "OXALIPLATIN HEXAL"、 "OXALIPLATIN INJECTION 50MG/10 MLand100MG/20ML"、 "oxaliplatin accord"、 "OXALIPLATIN KABI"、 "OXALIPLATIN SUN"、 "Oxaliplatin Pliva"、 "OXALIPLATIN Accord"、 "Oxaliplatin Teva"、 "Oxaliplatino SUN"、 "TEVAOXALI"、 "OXALIPLATIN 5MG/ML 20ML VIAL TEVA"、 "OXALIPLATIN INJECTION USP, 5 MG/ML, PACKAGED IN 50 MG/10 ML AND 100 MG"、 "OXALIPLATINA"、 "OXALIPLATINE WINTHROP"、 "OXALIPLATIN 100MG/20ML"、 "OXALIPLATIN INJECTION 50MG/10 M"、 "OXALIPLATINE TEVA 5 mg/ml"、 "Oksaliplatin Teva 5 MG/ML"、 "OXALIPLATIN INJECTION 50MG/10 ML+100MG/20ML"、 "OXALIPLATIN, 5MG/ML, UNAVAILABL"、 "Oxaliplatin Injection, USP"、 "oxaliplatin Accord"、 "Curatinox"、 "OXALIPLATINO (7351A)"、 "OXALIPLATINO ACCORD HEALTH"、 "OXALIPLATINO TEVA - 5 MG/ML CONCENTRATO PER SOL"、 "Oxaliplatin Mylan"、 "L-OHP"、 "OXALIPLATINUM"、 "Oxaliplatino SUN 5 mg/ml, concentrato per soluz"、 "COMPARATOR OXALIPLATIN"、 "OXALIPLATIN 5mg/ml CONCENTRATE FOR SOLUTION FOR INFUSION"、 "OXALIPLATIN, 20ML SAGENT"、 "OXALIPLATINE KABI"、 "OXALIPLATINO SUN - 5MG/ML CONCENTRATO PER SOLUZ"、 "Oxaliplatine"、 "RUIZHIBO (OXALIPLATIN)"、 "OXALIPLATIN INJ 100MG"、 "OXALIPLATIN Pliva"、 "OXALIPLATINE INFUUS"、 "OXALIPLATINO TEVA 5 MG / ML"、 "Oxaliplatin Solution for infusion"、 "Oxaliplatin, 55 MG/SQ M"、 "Elplat"、 "OXALIPLATIN (FOLFOX4-PROTOKOLL)"、 "OXALIPLATIN 100MG"、 "OXALIPLATIN AUROBINDO"、 "OXALIPLATIN FRESENIUS KABI"、 "OXALIPLATIN INJECTION"、 "OXALIPLATINE MYLAN 5 MG/ML POUD"、 "OXALIPLATINE TEVA 5 mg/ml,"、 "OXALIPLATINO KABI"、 "Oxaliplatin 100mg/20mL Hos"、 "Oxaliplatin 100mg/20mL Hospira"、 "Oxaliplatin Aurobindo"、 "Oxaliplatin Fresenius Kabi"、 "Oxaliplatin Injection"、 "Oxaliplatin SUN"、 "Oxaliplatin Solution for i"、 "Oxaliplatina"、 "ACCORD (OXALIPLATIN)"、 "AI HENG"、 "MANNITOL/OXALIPLATIN"、 "OXALIPLATIN 100 MG TEVA"、 "OXALIPLATIN 100MG/20ML VIAL TEV"、 "OXALIPLATIN EBEWE"、 "OXALIPLATIN MAYNE"、 "OXALIPLATIN OMNICARE"、 "OXALIPLATIN Teva"、 "OXALIPLATIN, 50 MG HOSPIRA"、 "OXALIPLATIN-TEVA 5 MG/ML, CONCE"、 "OXALIPLATINE TEVA 5 MG/ML,"、 "OXALIPLATINO SANDOZ"、 "OXALIPLATINO SUN"、 "OXALIPLATINO SUN - 5MG/ML CONCE"、 "OXITAN"、 "Oxaliplatino"、 "Oxaliplatino Hospira"、 "Sinoxal"、 "AXIPLATIN"、 "ELOXATINE 100 MG, LYOPHILI"、 "MEDOXA"、 "OXALIPLATIN Infusion"、 "OXALIPLATIN 50 MG/10 ML HOSPIRA"、 "OXALIPLATIN 5MG/ML 100MG VIAL."、 "OXALIPLATIN 5MG/ML 20 ML V"、 "OXALIPLATIN 5mg/ml POWDER"、 "OXALIPLATIN INJECTION, 100"、 "OXALIPLATIN MEDAC"、 "OXALIPLATIN OMNICARE 5 MG/"、 "OXALIPLATIN ^FRESENIUS KAB"、 "OXALIPLATIN injection"、 "OXALIPLATIN, 5MG/ML HOSPIRA"、 "OXALIPLATIN,SOLUTION FOR I"、 "OXALIPLATINA KABI 5MG/ML P"、 "OXALIPLATINE TEVA 5 MG/ML"、 "OXALIPLATINO ACCORD HEALTHCARE"、 "OXALIPLATINO AUROBINDO 5 M"、 "OXALIPLATINO KABI 5 MG/ML"、 "Oxaliplatin Aurobindo Concentrate for solution for infusion"、 "Oxaliplatin Concentrate for solution for infusion"、 "Oxaliplatin Injection USP, 5 mg/ml, packaged in 50 mg/10 mL and 100 mg"、 "Oxaliplatin Injection, USP (0517-1910-01)"、 "Oxaliplatine Accord"、 "Oxaliplatino ACCORD - ACCORD HEALTHCARE LIMITED"、 "SANDOZ LTD OXALIPLATIN"、 "X-PLAT"、 "[PSS GPN] Oxaliplatin DBL"、 "Axiplatin"、 "EVOXALI"、 "EXTERNAL-OXALIPLATIN"、 "OXALIBBS"、 "OXALIPLATIN 5 MG/ML 20 ML"、 "OXALIPLATIN 50MG/10ML HOSPIRA"、 "OXALIPLATIN 5mg/ml POWDER FOR SOLUTION FOR INFUSION"、 "OXALIPLATIN 68/M2"、 "OXALIPLATIN 85mg/m2"、 "OXALIPLATIN INFUSION"、 "OXALIPLATIN INJECTION 50MG/10 ML and 100MG/20ML"、 "OXALIPLATIN INTRAVENOUS IN"、 "OXALIPLATIN PLIVA"、 "OXALIPLATINA ACCORD"、 "OXALIPLATINE DAKOTA PHARM"、 "OXALIPLATINE INFOPL CONC 5MG/ML"、 "OXALIPLATINO ACCORD - 5 MG/ML C"、 "OXALIPLATINO ACCORD - ACCORD HE"、 "OXALIPLATINO AHCL"、 "OXALIPLATINO SUN 5 MG/ML C"、 "OXALIPLATINO TEVA - 5 MG/ML CON"、 "Oxaliccord"、 "Oxaliplatin Accord"、 "Oxaliplatin 50mg/10mL Hosp"、 "Oxaliplatin Aurobindo 5 mg/ml c"、 "Oxaliplatin Kabi"、 "Oxaliplatin Sandoz"、 "Oxaliplatin hospira"、 "Oxaliplatina Kabi"、 "Oxaliplatine Hospira"、 "Oxaliplatine Pfizer"、 "TN UNSPECIFIED (OXALIPLATI"、 "[PSS GPN] OXALIPLATIN DBL"、 "oxaliplatin Hospira"、 "oxaliplatine"、 "oxaliplatino"、 "ACCORD'S OXALIPLATIN"、 "AI KE BO KANG"、 "AIHENG"、 "AXIPLATIN 5 MG/ML CONCENTR"、 "AXIPLATIN 5 MG/ML KONZENTR"、 "Accord's Oxaliplatin"、 "BLINDED Oxaliplatin"、 "CURATINOX"、 "DBL OXALIPLATIN"、 "ELOXATIN (OXALIPLATIN)"、 "OKSALIPLATIN TEVA"、 "OKSALIPLATIN TEVA 5 MG/ML"、 "OKSALIPLATIN TEVA 5 MG/ML KONCE"、 "OXA (OXALIPLATIN)"、 "OXALI (OXALIPLATIN)"、 "OXALIPATIN"、 "OXALIPLANTIN INJECTION, SOLUTION FOR INJECTION,"、 "OXALIPLATIN ACCORD"、 "OXALIPLATIN WINTHROP"、 "OXALIPLATIN (7351A)"、 "OXALIPLATIN (DBL)"、 "OXALIPLATIN (FOLFOX)"、 "OXALIPLATIN (L-OHP)"、 "OXALIPLATIN (MANUFACTURER"、 "OXALIPLATIN 100 MG/20 ML HOSPIR"、 "OXALIPLATIN 100 MG/20ML HOPSPIR"、 "OXALIPLATIN 100MG/20ML 5MG/ML"、 "OXALIPLATIN 100MG/20ML TEVA PHARMACEUTICALS"、 "OXALIPLATIN 145MG"、 "OXALIPLATIN 5 MG/ML"、 "OXALIPLATIN 5mg/ml POWDER FOR SOLUTION FOR INFU"、 "OXALIPLATIN 85MG/M2"、 "OXALIPLATIN ACCORD HEALTHCARE"、 "OXALIPLATIN ACTAVIS"、 "OXALIPLATIN AHCL"、 "OXALIPLATIN ARROW"、 "OXALIPLATIN AUROBINDO CONC"、 "OXALIPLATIN Aurobindo 5 mg/ml Concentrate for s"、 "OXALIPLATIN CONCENTRATE FO"、 "OXALIPLATIN DAKOTA PHARM"、 "OXALIPLATIN FOR INJECTION"、 "OXALIPLATIN HOSPIRA 100MG/20ML"、 "OXALIPLATIN INJECTION 5OXA"、 "OXALIPLATIN INJECTION, USP (0517-1910-01)"、 "OXALIPLATIN INTAS"、 "OXALIPLATIN INTRAVENOUS INFUSION 200MG TEVA"、 "OXALIPLATIN OXALIPLATIN"、 "OXALIPLATIN PILVA"、 "OXALIPLATIN SUN PHARMA"、 "OXALIPLATIN ^ACCORD^"、 "OXALIPLATIN ^FRESENIUS KABI^"、 "OXALIPLATIN pliva"、 "OXALIPLATIN, 100 MG HOSPIRA"、 "OXALIPLATIN, 55 MG/SQ M"、 "OXALIPLATIN, 5MG/ML"、 "OXALIPLATIN,SOLUTION FOR INFUSION,5MG/ML"、 "OXALIPLATIN- PFIZER"、 "OXALIPLATIN-PLIVA"、 "OXALIPLATINA KABI"、 "OXALIPLATINA KABI 5mg/ml P"、 "OXALIPLATINE ACCORD 5 MG/M"、 "OXALIPLATINE ARROW"、 "OXALIPLATINE FRESENIUS"、 "OXALIPLATINE INFUUS INFUUS"、 "OXALIPLATINE INFUUS, 5 MG/"、 "OXALIPLATINE PFIZER"、 "OXALIPLATINE SUN"、 "OXALIPLATINE TEVA 5 mg/mL"、 "OXALIPLATINO ACCORD - 5 MG/ML CONCENTRATO PER S"、 "OXALIPLATINO ACCORD - ACCORD HEALTHCARE LIMITED"、 "OXALIPLATINO ACCORD 5 MG/ML CONCENTRATE FOR SOL"、 "OXALIPLATINO ACCORD HEALTHCARE - ACCORD HEALTHC"、 "OXALIPLATINO ACTAVIS 5 MG/ML"、 "OXALIPLATINO EBEWE"、 "OXALIPLATINO KABI - 5 MG/M"、 "OXALIPLATINO KABI - 5 MG/ML CONCENTRATO PER SOL"、 "OXALIPLATINO TEVA - 5 MG/M"、 "OXALIPLATINO TEVA - 5MG/ML CONCENTRATO PER SOLU"、 "OXALIPLATINO TEVA - TEVA I"、 "OXALIPLATINO TEVA 5 MG/ML"、 "OXALIPLATINO TEVA 5 mg/ml CONCENTRADO PARA SOLUCION PARA PERFUSION EFG"、 "OXALIPLATINO WINTHROP"、 "OXALIPLATINO(7351A)"、 "OXALPIN"、 "OXAPLATIN"、 "OXAPLATINO ACCORD"、 "OXIZALIPLATIN"、 "OZALIPLATIN"、 "Oksaliplatin Teva"、 "Oxaliplatin (Oxaliplatin)"、 "Oxaliplatin 5 mg/ml concentrate"、 "Oxaliplatin 50mg/10mL Hospira"、 "Oxaliplatin ACCORD"、 "Oxaliplatin Accord Healthcare"、 "Oxaliplatin Accord healthc"、 "Oxaliplatin Actavis"、 "Oxaliplatin Arrow"、 "Oxaliplatin Intas"、 "Oxaliplatin Intravenous In"、 "Oxaliplatin Omnicare"、 "Oxaliplatin PLIVA"、 "Oxaliplatin SUN 5 mg/ml Konzentrat zur Herstellung"、 "Oxaliplatin SUN 5 mg/ml concent"、 "Oxaliplatin Solution for infusi"、 "Oxaliplatin Sun"、 "Oxaliplatin accord healthcare"、 "Oxaliplatin sun"、 "Oxaliplatin teva"、 "Oxaliplatin winthrop"、 "Oxaliplatina Accord"、 "Oxaliplatine accord"、 "Oxaliplatino Aurobindo 5 mg/ml Concentrate for"、 "Oxaliplatino SUN 5 mg/ml, concentrato per soluzione per infusione"、 "Oxaliplatino Sun"、 "Oxaliplatino Teva 5mg/ml concentrado para soluc"、 "RIBOXATIN"、 "SANDOZ'S OXALIPLATIN"、 "oxaliplatin Teva"、 "oxaplatin"、 |
| Lobaplatin | "LOBAPLATIN"、 "Lobaplatin"、 "LOBAPLANTIN"、 |
| Capecitabine | "XELODA"、 "CAPECITABINE."、 "CAPECITABINE TAB 500MG"、 "CAPECITABINE TAB 500 MG"、 "CAPECITABINE 500MG"、 "CAPECITABINE 500MG TEVA"、 "CAPECITABINE TAB"、 "CAPECITABINE 500 MG"、 "CAPECITABINE 500MG ACCORD"、 "CAPECITABINE TAB 150MG"、 "CAPECITABINE ACCORD"、 "CAPECITABINE TAB 500mg"、 "CAPECITABINE TAB 500 MG BE"、 "CAPECITABINE 500MG ROXANE"、 "CAPECITABINE 500MG TAB"、 "Capecitabine, Unknown"、 "CAPECITABINE TAB 500 MG BER"、 "CAPECITABINE 500MG MYLAN"、 "CAPECITABINE 500 MG ACCORD"、 "CAPECITABINE 500MG WEST-WA"、 "CAPECITABINE 500 MG TEVA"、 "Capecitabine Accord"、 "CAPECITABINA"、 "CAPECITABINE (Manufacturer unkn"、 "CAPECITABINE TAB 150 MG"、 "Capecitabine (Unknown)"、 "CAPECITABINE 500MG GENENTECH"、 "CAPECITABINE 500MG WESTWAR"、 "CAPECITABINE 150MG"、 "CAPECITABINA ACCORD"、 "CAPECITABINE 500MG TEVA PHARMAC"、 "CAPECITABINE MYLAN"、 "Capecitabine 500 Mg"、 "CAPECITABINE 500MG WEST WA"、 "CAPECITABINE 500mg"、 "CAPECITABINE (Manufacturer"、 "CAPECITABINE 500MG ACCORD HEALTHCARE, INC"、 "CAPECITABINE TEVA"、 "CAPECITABINA (1224A)"、 "CAPECITABINE (Manufacturer unknown)"、 "CAPECITABINE 500 MG TABLET"、 "CAPECITABINE 500MG WEST-WARD PHARMACEUTICAL"、 "Capecitabine 500mg"、 "Capecitabine Film Coated Tablet"、 "CAPECITABINE 500MG TAB TEVA"、 "CAPECITABINE 500MG WEST WARD PHARMACEUTICALS"、 "CAPECITABINA MEDAC"、 "CAPECITABINE 500 MG RISING"、 "CAPECITABINE 500 MG WEST-"、 "CAPECITABINE 500 MG MYLAN"、 "CAPECITABINE 500MG WEST?WA"、 "CAPECITABINE 500 MG TAB"、 "CAPECITABINE 500 Mg"、 "CAPECITABINE 500MG TABLETS"、 "COMPARATOR CAPECITABINE"、 "Capecitabine Film Coated T"、 "CAPECITABINE 500 MG WEST-WARD"、 "CAPECTABINE TAB 500MG"、 "CAPECITABINE 500mg west-wa"、 "CAPECITABINE, 500 MG"、 "Capecitabine Tablets, USP"、 "capecitabine 500mg west-wa"、 "CAPECITABINE 500 mg"、 "CAPECITABINE 500MG UNKNOWN"、 "CAPECITABINE SANDOZ"、 "CAPECITABINE tab 500mg"、 "CAPECITABINE 150MG ACCORD"、 "CAPECITABINE 150MG TEVA"、 "CAPECITABINE 500 MG WEST-W"、 "CAPECITABINE 500 MG WESTWA"、 "CAPECITABINE 500MG ACCORD HEALT"、 "CAPECITABINE MEDAC"、 "CAPECITABINE TAB 500MG"、 "CAPECITABINE tab 500 MG"、 "CAPECITABIN ACCORD"、 "CAPECITABINE 150 MG TEVA"、 "CAPECITABINE 500 MG WEST W"、 "CAPECITABINE 500 MG tab"、 "CAPECITABINE 500 mg tab"、 "CAPECITABINE 500MG GENENTE"、 "CAPECITABINE 500MG TAB MYLAN"、 "CAPECITABINE 500MG TABLETS TEVA"、 "CAPECITABINE TAB 500 mg"、 "CAPECITABINE TABLETS"、 "CAPECITABINE++ 500MG TEVA"、 "Capecitabine 500 mg"、 "Capecitabine 500 mg West?W"、 "Capecitabine Tablets USP"、 "capecitabine tab 500mg"、 "CAPECITABINE 150 MG MYLAN PHARMACEUTICALS INC."、 "CAPECITABINE 150MG ROXANE"、 "CAPECITABINE 2000 MG MYLAN"、 "CAPECITABINE 500 MG ACCORD HEALTHCARE"、 "CAPECITABINE 500 MG GENENTECH"、 "CAPECITABINE 500 MG TABLETS TEV"、 "CAPECITABINE 500 MG WEST WARD"、 "CAPECITABINE 500MG HIKMA"、 "CAPECITABINE 500MG TAB TEVA PHA"、 "CAPECITABINE TAB 150mg"、 "CAPECITABINE TABLET FO 500MG"、 "CAPECITABINE, 500MG"、 "Capecitabin"、 "Capecitabina"、 "Capecitabine TAB 500 Mg"、 "PECASET ACCORD"、 "APO-CAPECITABINE"、 "CAPECITABIN HEXAL"、 "CAPECITABINE 1000 MG MYLAN"、 "CAPECITABINE 150MG TABLET"、 "CAPECITABINE 150MG/500MG W"、 "CAPECITABINE 3,000 MG MYLAN"、 "CAPECITABINE 500MG TABLET"、 "CAPECITABINE 500MG TEVA PH"、 "CAPECITABINE 500mg Genente"、 "CAPECITABINE GENENTECH"、 "CAPECITABINE TAB150MG"、 "CAPECITABINE TABLET FO 150MG"、 "CAPECITABINE, UNKNOWN"、 "Capecitabina Accord"、 "Capecitabine 150mg"、 "SYMLODA"、 "capecitabine 500mg west?wa"、 "CAPCITABINE"、 "CAPECITABIN"、 "CAPECITABIN ACTAVIS"、 "CAPECITABINA MEDAC 150 MG"、 "CAPECITABINA MYLAN"、 "CAPECITABINE 500 MG WESTW"、 "CAPECITABINE 150 MG"、 "CAPECITABINE 150MG TABLET TEVA"、 "CAPECITABINE 150MG TABLETS"、 "CAPECITABINE 3300MG WEST-WARD"、 "CAPECITABINE 500"、 "CAPECITABINE 500 ACCORD"、 "CAPECITABINE 500 MG ACCORD HEALTHCARE INC"、 "CAPECITABINE 500 MG TABS 1"、 "CAPECITABINE 500 MG WESTWARD"、 "CAPECITABINE 500 mg TAB"、 "CAPECITABINE 500 mg Teva"、 "CAPECITABINE 500MG TAB"、 "CAPECITABINE 500MG ACCORDA"、 "CAPECITABINE 500MG GENENET"、 "CAPECITABINE 500MG HIKMA P"、 "CAPECITABINE 500MG TAB RISING"、 "CAPECITABINE 500MG TABS MY"、 "CAPECITABINE 500MG WESTWARD"、 "CAPECITABINE 500MG Westwar"、 "CAPECITABINE 500MG west-wa"、 "CAPECITABINE 500mg WEST?WA"、 "CAPECITABINE TABLETS 500 MG TEV"、 "CAPECITABINE, 500MG ACCORD HEAL"、 "CAPECITABINE500MG ROXANE"、 "CAPECTIABINE 500MG UNKNOWN"、 "Capecitabine 500mg Tab"、 "TEVA?CAPECITABINE"、 "capecitabine 500mg"、 "CAPACETABINE"、 "CAPCITABINE TAB 500 MG"、 "CAPECITABINE 500MG WEST-W"、 "CAPECITABINE 500MG WESTWA"、 "CAPECITABINE (1224A)"、 "CAPECITABINE / 150MG TEVA"、 "CAPECITABINE 150MG TEVA PHARMACEUTICALS"、 "CAPECITABINE 500 GENENTECH"、 "CAPECITABINE 500 MG ACCORD HEAL"、 "CAPECITABINE 500 MG GENENT"、 "CAPECITABINE 500 MG GENETECH"、 "CAPECITABINE 500 MG ROXANE"、 "CAPECITABINE 500 MG TAB ACCO"、 "CAPECITABINE 500 MG TAB TEVA PH"、 "CAPECITABINE 500 MG TEVA PHARMA"、 "CAPECITABINE 500 MG WEST?W"、 "CAPECITABINE 500 MG, TEVA"、 "CAPECITABINE 500 MYLAN"、 "CAPECITABINE 500 TEVA PHARMACEU"、 "CAPECITABINE 500 mg TAB ACCO"、 "CAPECITABINE 500 mg West?W"、 "CAPECITABINE 500MG + 150MG ACCO"、 "CAPECITABINE 500MG AMNEAL"、 "CAPECITABINE 500MG AND 150 ACCORD"、 "CAPECITABINE 500MG AND 150MG TE"、 "CAPECITABINE 500MG Genente"、 "CAPECITABINE 500MG TAB TEV"、 "CAPECITABINE 500MG TEVA PHARMACEUTICALS USA INC"、 "CAPECITABINE 500MG UNK"、 "CAPECITABINE 500MG+150MG ACCORD"、 "CAPECITABINE 500MGS"、 "CAPECITABINE 500mg Westwar"、 "CAPECITABINE BIOGARAN"、 "CAPECITABINE NORMON"、 "CAPECITABINE NORTHSTAR"、 "CAPECITABINE TAB 150 mg"、 "CAPECITABINE+BLINDED STUDY DRUG"、 "CAPECITABINE, 150MG"、 "CAPECITANINE 150MG AND 500MG"、 "Capcitabine"、 "Capecitabine Biogaran"、 "Capecitabine Tab 500Mg"、 "Capecitabine accord"、 "Capecitabine tab 500MG"、 "Capecitabine tablets"、 "Capecitabine, 500 MG"、 "Capecitabine, 500mg"、 "capecitabine 500mg West?wa"、 "capecitabine 500mg tab"、 "ACCORD'S CAPECITABINE"、 "Accord's Capecitabine"、 "BLINDED CAPECITABINE"、 "BLINDED Capecitabine"、 "BLINDED STUDY MEDICATION+CAPECI"、 "Blinded Capecitabine"、 "CAPACITABINA 500MG TAB"、 "CAPACITABINE"、 "CAPACITABINE 500MG TEVA PHARMAC"、 "CAPACITABINE ACCORD"、 "CAPE750MG/M^2 GENENTECH"、 "CAPEACITABINE 150 MG TABLET PFI"、 "CAPEBINE"、 "CAPECETABINE- SUB FOR XELODA. 500 MG"、 "CAPECIGABINE 500MG"、 "CAPECITAB INE 500MG TEVA PHARMA"、 "CAPECITABIN STADA"、 "CAPECITABIN ^ACTAVIS^"、 "CAPECITABIN ^STADA^"、 "CAPECITABINA ACCORD - ACCORD HE"、 "CAPECITABINA ACCORD - ACCORD HEALTHCARE LIMITED"、 "CAPECITABINA ACCORD 500 MG"、 "CAPECITABINA ACCORD 500 MG COMPRIMIDOS RECUBIERTOS CON PELICULA EFG"、 "CAPECITABINA ACTAVIS"、 "CAPECITABINA MEDAC 500 MG FILM-"、 "CAPECITABINA MYLAN 500 MG COMPR"、 "CAPECITABINA ZENTIVA"、 "CAPECITABINA(1224A)"、 "CAPECITABINE 500 MG"、 "CAPECITABINE 500 MG"、 "CAPECITABINE 500 MG TAB"、 "CAPECITABINE 500 Mg"、 "CAPECITABINE 500 mg tab"、 "CAPECITABINE 500MG"、 "CAPECITABINE 500MG TAB"、 "CAPECITABINE 500MG TEVA"、 "CAPECITABINE TAB 150MG"、 "CAPECITABINE TAB 500MG"、 "CAPECITABINE , 500MG"、 "CAPECITABINE /500 MG TEVA"、 "CAPECITABINE 1000MG MYLAN"、 "CAPECITABINE 150 ACCORD"、 "CAPECITABINE 150 MG TEVA"、 "CAPECITABINE 150 Mg"、 "CAPECITABINE 1500 MG BID"、 "CAPECITABINE 1500 MG MYLAN"、 "CAPECITABINE 1500MG MYLAN"、 "CAPECITABINE 150MG ACCORD HEALTHCARE"、 "CAPECITABINE 150MG AMNEAL"、 "CAPECITABINE 150MG TAB WES"、 "CAPECITABINE 150MG TEVA PHARMAC"、 "CAPECITABINE 150MG WEST-WA"、 "CAPECITABINE 150MG WESTWAR"、 "CAPECITABINE 150mg"、 "CAPECITABINE 200MG MYLAN"、 "CAPECITABINE 3,000MG MYLAN"、 "CAPECITABINE 500 MG BDI PHARMA"、 "CAPECITABINE 500 MG HIKMA"、 "CAPECITABINE 500 MG MYLAN / TAB"、 "CAPECITABINE 500 MG MYLAN PHARM"、 "CAPECITABINE 500 MG MYLAN PHARMACEUTICALS INC."、 "CAPECITABINE 500 MG TAB MYLAN"、 "CAPECITABINE 500 MG TAB TE"、 "CAPECITABINE 500 MG TABLET MYLAN"、 "CAPECITABINE 500 MG TABLET PFIZ"、 "CAPECITABINE 500 MG TABLET TEVA"、 "CAPECITABINE 500 MG TABLET UNKNOWN"、 "CAPECITABINE 500 MG TABLETS ACCORD"、 "CAPECITABINE 500 MG TABS M"、 "CAPECITABINE 500 MG TEVA PHARMACEUTICALS USA IN"、 "CAPECITABINE 500 MG tablet"、 "CAPECITABINE 500 MGTAB"、 "CAPECITABINE 500 WEST-WARD"、 "CAPECITABINE 500 mg WEST-W"、 "CAPECITABINE 500 mg genentech"、 "CAPECITABINE 500MG TEVA"、 "CAPECITABINE 500MG ACCORD HEALTHCARE INC."、 "CAPECITABINE 500MG GENERIC"、 "CAPECITABINE 500MG GENETECH"、 "CAPECITABINE 500MG MYLAN PHARMACEUTICALS"、 "CAPECITABINE 500MG SPS"、 "CAPECITABINE 500MG TAB TEVA"、 "CAPECITABINE 500MG TAB ACCO"、 "CAPECITABINE 500MG TAB GEN"、 "CAPECITABINE 500MG TAB GENENTECH"、 "CAPECITABINE 500MG TABS TEVA PH"、 "CAPECITABINE 500MG TEVA PHARMACUETICALS"、 "CAPECITABINE 500MG TEVA TEVA"、 "CAPECITABINE 500MG, CAPECI"、 "CAPECITABINE 500MG/150MG TEVA"、 "CAPECITABINE 500mg TAB"、 "CAPECITABINE 500mg TABLETS"、 "CAPECITABINE 50MG TEVA"、 "CAPECITABINE ACCORD 300 MG"、 "CAPECITABINE ACTAVIS (CAPECITAB"、 "CAPECITABINE ACTAVIS FILM COATED TABLET 500 MG"、 "CAPECITABINE AMNEAL PHARMACEUTICALS"、 "CAPECITABINE FILM COATED T"、 "CAPECITABINE FILM-COATED T"、 "CAPECITABINE FILMOMHULDE T"、 "CAPECITABINE Filmomhulde tablet, 500 mg (milligram)"、 "CAPECITABINE GENETEC"、 "CAPECITABINE TAB 500 MG"、 "CAPECITABINE TAB 500 Mg"、 "CAPECITABINE TAB 500MG ACCORD"、 "CAPECITABINE TAB 500MT"、 "CAPECITABINE TAB 500Mg"、 "CAPECITABINE TABLET"、 "CAPECITABINE TABLET AMNEAL"、 "CAPECITABINE TABLETS, USP"、 "CAPECITABINE TEVA 500 MG"、 "CAPECITABINE TEVA 500MG"、 "CAPECITABINE tab 500 mg"、 "CAPECITABINE++ 150MG TEVA"、 "CAPECITABINE++ 500MG OTHER"、 "CAPECITABINE, 150 MG"、 "CAPECITABINE, 500 MG GENEN"、 "CAPECITABINE, 500 MG RISIN"、 "CAPECITABINE, 500MG MYLAN"、 "CAPECITBINE 500MG ACCORDA"、 "CAPECITIABINE"、 "CAPECTABINE TAB 500 MG"、 "Capecitabin accord"、 "Capecitabina Actavis"、 "Capecitabina Mylan"、 "Capecitabina Mylan 500 mg"、 "Capecitabine 500"、 "Capecitabine 500 Mg Tab ACCO"、 "Capecitabine 500 mg west-w"、 "Capecitabine 500mg tablets"、 "Capecitabine ACCORD"、 "Capecitabine Tab 500mg"、 "Capecitabine ^Accord"、 "Capecitabine tablet"、 "KAPECITABIN"、 "KAPECITABINE"、 "KAPECITABINE TEVA"、 "PECASET"、 "Pecaset"、 "Pecaset Accord"、 "TUTABIN"、 "capacitabine"、 "capecitabin"、 "capecitabine 500 mg"、 "capecitabine accord"、 |
| Paclitaxel | "PACLITAXEL."、 "ABRAXANE"、 "TAXOL"、 "PACLITAXEL/PACLITAXEL LIPOSOME"、 "PACLITAXEL (Manufacturer u"、 "NAB-PACLITAXEL"、 "NAB-Paclitaxel"、 "PACLITAXEL (Manufacturer unknown)"、 "PACLITAXEL/PACLITAXEL LIPO"、 "PACLITAXEL 6mg/ml CONCENTR"、 "PACLITAXEL ALBUMIN"、 "PACLITAXEL KABI"、 "PACLITAXEL (Manufacturer unknow"、 "PACLITAXEL 6mg/ml CONCENTRATE FOR SOLUTION FOR"、 "Paclitaxel Hospira"、 "PACLITAXEL 6mg/ml CONCENTRATE F"、 "COMPARATOR PACLITAXEL"、 "Paclitaxel (Unknown)"、 "PACLITAXEL SANDOZ"、 "Nab-paclitaxel"、 "PACLITAXEL ACCORD"、 "ANZATAX (PACLITAXEL)"、 "PACLITAX NAB"、 "PACLITAXEL HOSPIRA"、 "Paclitaxel (ATLLC)"、 "Nab-Paclitaxel"、 "Paclitaxel Accord"、 "NAB?PACLITAXEL"、 "HUMAN ALBUMIN/PACLITAXEL"、 "PACLITAXEL EBEWE"、 "PACLITAXEL PROTEIN-BOUND PARTIC"、 "PAXEL"、 "PACLITAXEL INJECTION"、 "PACLITAXEL TEVA ITALIA"、 "NAB?Paclitaxel"、 "Anzatax"、 "PACLIINJ-A"、 "PACLITAXEL TEVA"、 "EBETAXEL"、 "BLINDED PACLITAXEL"、 "PACLITAXEL TEVA 6 MG/ML, C"、 "Sindaxel"、 "ALBUMIN-BOUND PACLITAXEL"、 "PACLITAXEL INJECTION, USP"、 "Genexol-PM"、 "PACLITAXEL 6mg/ml CONCENTRATE FOR SOLUTION FOR INFUSION"、 "Paclitaxel Kabi"、 "PACLITAXEL (2698A)"、 "PACLITAXEL TEVA 6 MG/ML"、 "Evotaxel"、 "PACLITAXEL MYLAN"、 "PACLITAXEL TEVA - 6 MG/ML CONCE"、 "PACLITAXEL TEVA - 6 MG/ML CONCENTRATO PER SOLUZ"、 "Paxitas"、 "NEOTAXAN"、 "RIBOTAX"、 "PACLITAXEL KABI 6 mg/ml, s"、 "Paclitaxel Actavis"、 "nab-paclitaxel"、 "PACLITAXEL TEVA 6 mg/ml CONCENTRADO PARA SOLUCION PARA PERFUSION EFG,"、 "PACLITAXEL 6 MG/ML 50 ML V"、 "PACLITAXEL, ABI-007 PROTEIN BOU"、 "Paclitaxel Pliva"、 "TARVEXOL"、 "paclitaxel kabi"、 "INTAXEL (PACLITAXEL)"、 "NAB PACLITAXEL"、 "Nab?paclitaxel"、 "PACLITAXEL 6 mg/ml 50 ml v"、 "PACLITAXEL ACCORD HEALTHCA"、 "PACLITAXEL AUROVITAS"、 "PACLITAXEL INTRAVENOUS INFUSION"、 "PACLITAXEL LIPOSOME"、 "PACLITAXEL NK"、 "PACLITAXEL TEVA 6MG/ML CONCENTRADO PARA SOLUCIO"、 "PAXENE (PACLITAXEL)"、 "Paclitaxel 30mg/5mL Hospira"、 "Paclitaxel Aurobindo 6 mg/ml Concentrate for so"、 "Paclitaxel protein?bound p"、 "paclitaxel, 6 MG/ML"、 "LIPOSOMAL PACLITAXEL"、 "Nab?Paclitaxel"、 "PACLITAXEL 300 MG TEVA"、 "PACLITAXEL AUROVITAS CONCENTRAT"、 "PACLITAXEL-TEVA LIQUID"、 "Paclitaxel 6 mg/ml"、 "Paclitaxel 6 mg/ml 50 ml v"、 "Paclitaxel Aurovitas 6 mg/ml Concentrate for so"、 "UNITAXEL"、 "PACLITAXEL 300MG/50ML"、 "PACLITAXEL 6MG/ML"、 "Paclitaxel 30mg/5mL Hospir"、 "Paclitaxel Arrow"、 "Paclitaxel Aurovitas"、 "Paclitaxel Mylan Generics 6 mg/"、 "SINDAXEL"、 "Accord Paclitaxel"、 "KABI PACLITAXEL"、 "LIPUSU"、 "ONTAX"、 "PACITAXEL"、 "PACITAXEL 6MG/ML, 50ML VIAL"、 "PACLITAXEL 5MG/ML 50ML VIAL HOSPIRA"、 "PACLITAXEL AHCL"、 "PACLITAXEL ALBUMIN BOUND"、 "PACLITAXEL Accord"、 "PACLITAXEL COMP-PAC+"、 "PACLITAXEL INTRAVENOUS INF"、 "PACLITAXEL TEVA 6 MG/ML CO"、 "PACLITAXEL TEVA 6 mg/ml CONCENTRADO PARA SOLUCI"、 "Paclitaxel 100mg/16.7mL Ho"、 "Paclitaxel ?Mylan?"、 "paclitaxel Accord"、 "Lipusu"、 "NANOPARTICLE ALBUMIN-BOUND-PACLITAXEL"、 "Nanoparticle albumin-bound"、 "PACLITAXEL 6 mg/ml 50 ml"、 "PACLITAXEL 6/MG/ML 50ML VIAL"、 "PACLITAXEL 6MG/ML 50 ML MULTIDO"、 "PACLITAXEL ACCORD HEALTHCARE ITALIA"、 "PACLITAXEL FRESENIUS KABI"、 "PACLITAXEL OMNICARE"、 "PACLITAXEL PLIVA"、 "PACLITAXEL PROTEIN-BOUND PARTICLES (ALBUMIN-BOU"、 "PACLITAXEL TEVA - TEVA ITALIA S"、 "PACLITAXEL, 300MG FRESENIUS KAB"、 "PRAXEL"、 "Paclitax Nab"、 "Paclitaxel Aurovitas conce"、 "Paclitaxel accord"、 "Paclitaxel concentrate for"、 "CELLTAXEL"、 "LIPOSOME ENTRAPPED PACLITAXEL"、 "MEDAC PACLITAXEL"、 "PACITAXEL 6MG/ML TEVA"、 "PACLITAXEL 300MG APP"、 "PACLITAXEL 6MG/ML 50 ML VI"、 "PACLITAXEL 6MG/ML 50ML HOSPIRA"、 "PACLITAXEL ACCORD HEALTHCARE"、 "PACLITAXEL ACTAVIS"、 "PACLITAXEL INJECTION 100/16.7MG/ML AND 300/50MG/ML"、 "PACLITAXEL ONKOVIS"、 "PACLITAXEL PROTEIN-BOUND/ALBUMIN"、 "PACLITAXEL STRAGEN"、 "PACLITAXEL TEVA - TEVA ITALIA S.R.L."、 "PACLITAXEL TEVA 6MG/ML CONCENTR"、 "PACLITAXEL TEVA ? 6 MG/ML"、 "PACLITAXEL, 353MG"、 "PAKLITAKSEL KABI 6 MG/ML"、 "Paclitaxel Mylan 6 mg/ml"、 "Paclitaxel Mylan Generics 6 mg/ml"、 "nab-Paclitaxel"、 "ANZATAX (paclitaxel)"、 "ANZATAX /01116001/"、 "APO-PACLITAXEL"、 "ATAXIL"、 "Accord's Paclitaxel"、 "LIPOSOME"、 "NANO PARTICLE ALBUMIN BOUN"、 "Neotaxan"、 "PACLITAXEL - CONCENTRATE F"、 "PACLITAXEL 155 MG TEVA"、 "PACLITAXEL 6MG/ML 50 ML MULTIDOSE VIAL PHARMACH"、 "PACLITAXEL 80 MG/M2"、 "PACLITAXEL 80MG/M2"、 "PACLITAXEL 80mg/m2"、 "PACLITAXEL HOSPIRA 6 MG/ML"、 "PACLITAXEL INFOPL CONC 6MG/ML"、 "PACLITAXEL INFUUS"、 "PACLITAXEL INJECTION USP 6 mg/m"、 "PACLITAXEL KABI - 6 MG/ML CONCE"、 "PACLITAXEL KABI 6 MG/ML CO"、 "PACLITAXEL MEDAC"、 "PACLITAXEL TEVA - TEVA ITA"、 "PACLITAXEL TEVA CONCENTRAT"、 "PACLITAXEL USP"、 "PACLITAXEL, 100 MG ACTAVIS PHAR"、 "PACLITAXEL, 100 MG HOSPIRA"、 "PACLITAXEL, 50 MG/ML"、 "PACLITAXEL, 6 MG/ML"、 "PACLITAXEL, 6 MG/ML WG CRITICAL"、 "PACLITAXEL, 6MG/ML"、 "PACLITAXEL, ABI-007 PROTEIN BOUND"、 "PACLITAXEL, ALBUMIN BOUND"、 "Paclitaxel 100mg/16.7mL Hospira"、 "Paclitaxel 6 mg/ml, concen"、 "Paclitaxel Fresenius Kabi"、 "Paclitaxel MYLAN"、 "Paclitaxel Teva"、 "Paclitaxel accord healthcare"、 "[PSS GPN] PACLITAXEL"、 "paclitaxel 6mg/ml"、 "ALBUMIN?BOUND PACLITAXEL"、 "ANZATAX PACLITAXEL"、 "ANZATAX(PACLITAXEL)"、 "APO-PACLITAXEL INJECTABLE"、 "AXITAXEL"、 "Accord paclitaxel"、 "Albumin?bound paclitaxel"、 "BLINDED Paclitaxel"、 "Celltaxel"、 "EBETAXEL 30MG/5M"、 "Ebetaxel"、 "FRESENIUS KABI PACLITAXEL"、 "INN-PACLITAXEL"、 "KUURSCHEMA MET PACLITAXEL"、 "MEDAXOL"、 "NAB-PACLITAXEL 100MG VIAL"、 "NAB-PACLITAXEL ALBUMIN"、 "NAB?paclitaxel"、 "NABPACLITAXEL"、 "NANO PARTICLE ALBUMIN-BOUN"、 "NANOPARTICLE ALBUMIN-BOUND PACL"、 "NAP - PACLITAXEL"、 "Nab Paclitaxel"、 "Nab-PACLITAXEL"、 "Nano particle albumin-bound paclitaxel"、 "PACITAXOL"、 "PACLITACEL"、 "PACLITAXEL 300MG/50ML"、 "PACLITAXEL (IN.PACT ADMIRA"、 "PACLITAXEL (MANUFACTURER U"、 "PACLITAXEL (NOT SPECIFIED)"、 "PACLITAXEL (OPEN LABEL)"、 "PACLITAXEL +PHARMA"、 "PACLITAXEL - CONCENTRATE FOR SOLUTION FOR INFUSION"、 "PACLITAXEL 150/25ML MDV"、 "PACLITAXEL 288 MG (CICLO 1"、 "PACLITAXEL 300MG HOSPIRA"、 "PACLITAXEL 80 MG/KG"、 "PACLITAXEL 91 MG"、 "PACLITAXEL ACCORD 6 MG/ML"、 "PACLITAXEL ACCORD HEALTHCARE ITALIA 6 MG/ML, CONCENTRATO PER SOLUZIONE"、 "PACLITAXEL ACCORD HEALTHCARE ITALY"、 "PACLITAXEL ACCRORD"、 "PACLITAXEL AHCL 6 mg/ml, s"、 "PACLITAXEL ALBUMIN BOUND 100MG/M2"、 "PACLITAXEL ARROW"、 "PACLITAXEL AUROBINDO 6 MG/"、 "PACLITAXEL CONCENTRATE FOR"、 "PACLITAXEL HEXAL"、 "PACLITAXEL HOSPIRA 6 mg/ml"、 "PACLITAXEL INFUSION"、 "PACLITAXEL INJ 100MG"、 "PACLITAXEL INJECTION CONCE"、 "PACLITAXEL INJECTION USP 6 MG/M"、 "PACLITAXEL INJECTION USP 6 mg/ml"、 "PACLITAXEL KABI - 6 MG/ML CONCENTRATO PER SOLUZ"、 "PACLITAXEL KABI 6 MG/ML KONCENT"、 "PACLITAXEL KABI 6 MG/ML, SOLUTION ? DILUER POU"、 "PACLITAXEL MDV 6MG/ML NOVAPLUS/HOSPIRA"、 "PACLITAXEL MYLAN 6 MG/ML K"、 "PACLITAXEL MYLAN GENERICS"、 "PACLITAXEL MYLAN GENERICS - MYL"、 "PACLITAXEL NEOTAXAN 300 MG/50 ML"、 "PACLITAXEL OMNICARE 6 MG/M"、 "PACLITAXEL ONCOTRADE"、 "PACLITAXEL OPLOSSING VOOR"、 "PACLITAXEL Oplossing voor infuus, 6 mg/ml (milligram per milliliter)"、 "PACLITAXEL PACLITAXEL"、 "PACLITAXEL PILVA"、 "PACLITAXEL PROFUSIO"、 "PACLITAXEL PROTEIN-BOUND P"、 "PACLITAXEL PROTEIN-BOUND/ALBUMI"、 "PACLITAXEL PROTEIN?BOUND/A"、 "PACLITAXEL SANDOZ 6 MG/ML"、 "PACLITAXEL TEVA - 6 MG/ ML CONCENTRATO PER SOLU"、 "PACLITAXEL TEVA - TEVA ITALIA S.R.L"、 "PACLITAXEL TEVA 6 MG/ML, S"、 "PACLITAXEL TEVA 6 mg/ml"、 "PACLITAXEL TEVA 6MG/ML"、 "PACLITAXEL TEVA 6MG/ML CONCETRADO PARA SOLUCION"、 "PACLITAXEL TEVA ITALIA - TEVA I"、 "PACLITAXEL TEVA ITALIA S.R.L."、 "PACLITAXEL ^PHARMACHEMIE^"、 "PACLITAXEL, 100 MG/16.7 ML"、 "PACLITAXEL, 300MG"、 "PACLITAXEL, 300MG/5ML"、 "PACLITAXEL, 6 MG/ML TEVA"、 "PACLITAXEL,PACLITAXEL LIPO"、 "PACLITAXEL- TEVA"、 "PACLITAXELUM"、 "PACLITAXIL"、 "PACLITAXIN 6 MG/ML CONCENTRAAT VOOR OPLOSSING V"、 "PACLITAXOL INJECTION (ZGP) 30MG/5ML, 150MG/25ML"、 "PCLITAXEL"、 "PLACLITAXEL"、 "Paclitax"、 "Paclitaxel 6mg/ml"、 "Paclitaxel Comp-PAC+"、 "Paclitaxel Comp?PAC+"、 "Paclitaxel Injection"、 "Paclitaxel Injection USP"、 "Paclitaxel Mylan 6 mg/ml concentrado para solu??o para perfus?o"、 "Paclitaxel Mylan Generics"、 "Paclitaxel Sandoz"、 "Paclitaxel ^NK^"、 "Paclitaxel albumin"、 "Paclitaxel concentrate for solution for infusion"、 "Paclitaxel hospira"、 "Paclitaxel kabi"、 "Paclitaxel nab"、 "Paclitaxel profusio"、 "Pactal"、 "Sindaxel 6 mg/ml concentrat pen"、 "Stritoxol"、 "TAXAN (UNK INGREDIENTS)"、 "TAXONAB"、 "TEVA UK PACLITAXEL"、 "Taxomedac"、 "nab?paclitaxel"、 "neotaxan"、 "paclitaxel EBEWE"、 "paclitaxel Medac"、 "paclitaxel accord healthcare italy"、 "paclitaxel ahcl"、 "paxel"、 |
| Docetaxel | "TAXOTERE"、 "DOCETAXEL."、 "Docetaxel Accord"、 "DOCETAXEL ACCORD"、 "DOCETAXEL SANDOZ"、 "Docetaxel Hospira"、 "DOCETAXEL HOSPIRA"、 "Docetaxel (Unknown)"、 "DOCETAXEL ACTAVIS"、 "ONETAXOTERE"、 "DOCETAXEL HYDRATE"、 "DOCETAXEL INJECTION"、 "Docetaxel Actavis"、 "DOCETAXEL (Manufacturer unknown)"、 "DOCETAXEL (Manufacturer un"、 "Docetaxel Aurovitas"、 "DOCETAXEL (Manufacturer unknown"、 "DOCETAXEL ^Actavis^"、 "Doxetacel"、 "DOCETAXEL INJECTION CONCENTRATE"、 "DOCETAXEL TEVA"、 "DOCETAXEL SANOFI"、 "DOCETAXEL SANOFI-AVENTIS"、 "DOCETAXOL"、 "CAMITOTIC"、 "DOCETAXEL INTRAVENOUS INFU"、 "DOXITAXEL"、 "Docetaxel accord"、 "Camitotic"、 "DOCETAXEL Accord"、 "DOCETAXEL Injection"、 "DOCETAXEL SUN"、 "DOCETAXEL CONCENTRATE"、 "CAMITOTIC 20 MG/ML"、 "Docetaxel (Actavis)"、 "Docetaxel Teva"、 "DOCETAXEL (SANOFI-AVENTIS)"、 "DOCETAXEL ACCORD HEALTHCAR"、 "EBEDOCE"、 "DOCETAXEL WINTHROP"、 "DOCETAXEL HOSPIRA 10 mg/ml"、 "DOCETAXEL SAGENT"、 "Docetaxel Injection"、 "Docetaxel SUN"、 "docetaxel Accord"、 "COMPARATOR DOCETAXEL"、 "DOCETAXEL MCKESSON"、 "DOCETAXEL TRIHYDRATE"、 "doc?taxel"、 "docetaxel injection"、 "DOCETAXEL 10 MG/ML SANDOZ"、 "DOCETAXEL OMNICARE"、 "DOCETAXEL PFIZER"、 "Docetaxel 80mg/8mL Hospira"、 "Docetaxel Injection non-alcohol formula"、 "DOCETAXEL (Teva product cannot be excluded)"、 "DOCETAXEL ACCORD HEALTHCARE"、 "DOCETAXEL EBEWE"、 "DOCETAXEL INJECTION USP, 20 MG/0.5 ML, SINGLE-DOSE VIAL, TWO-VIAL FORM"、 "DOCETAXEL RATIOPHARM ITALI"、 "DOCETAXEL SANOFI AVENTIS"、 "DOCETAXEL SANOFI?AVENTIS"、 "DOCETAXEL ^ACTAVIS^"、 "DOPAFEI"、 "DOXEL [DOCETAXEL]"、 "Docetaxel Injection USP, 20 mg/0.5 mL, Single-dose Vial, Two-Vial Form"、 "Docetaxel hydrate"、 "TAXOTEL"、 "[PSS GPN] DOCETAXEL"、 "DOCETAXEL (7394A)"、 "DOCETAXEL 10 MG/ML 8 ML VIAL SANDOZ"、 "DOCETAXEL 160 MG-16 ML MDV"、 "DOCETAXEL 160MG/16ML"、 "DOCETAXEL 20MGX3 NOVA PLUS"、 "DOCETAXEL AUROVITAS 20 MG/ML CONCENTRADO PARA SOLUCION PARA PERFUSION"、 "DOCETAXEL CONCENTRAAT VOOR OPLO"、 "DOCETAXEL- SANOFI-AVENTIS"、 "DOXETAXEL"、 "Docetaxel Sandoz"、 "Docetaxel Sanofi?Aventis"、 "AISU [DOCETAXEL]"、 "AOMINGRUN"、 "DOCETAXEL (SANOFI)"、 "DOCETAXEL 10MG/ML 16ML VIA"、 "DOCETAXEL 120MG 250ML"、 "DOCETAXEL 160 MG/16 ML. HO"、 "DOCETAXEL ACCORD 80 MG/ 4 ML CONCENTRATE FOR SOLUTION FOR INFUSION"、 "DOCETAXEL ACCORD HEALTH CA"、 "DOCETAXEL Aurovitas"、 "DOCETAXEL MYLAN"、 "DOCETAXEL NC"、 "DOCETAXEL SUN PHARMA"、 "DOCETAXEL WINTHROP"、 "DOCETAXEL ^HOSPIRA^"、 "DOCETAXEL?ACCORD"、 "Docetaxel 120mg/12mL Hospira"、 "Docetaxel Kabi"、 "Docetaxol"、 "Duopafei"、 "EXTERNAL-DOCETAXEL"、 "TOLNEXA"、 "AO MING RUN"、 "Accord's Docetaxel"、 "BLINDED DOCETAXEL"、 "CHEMOTHERAPY DRUG W/ TAXOTERE"、 "DAXOTEL (DOCETAXEL)"、 "DAXOTEL [DOCETAXEL]"、 "DOC?TAXEL"、 "DOC?TAXEL TRIHYDRAT?"、 "DOCATAXEL"、 "DOCE NC"、 "DOCELIBBS"、 "DOCETAXEL (DOC)"、 "DOCETAXEL (DOCETAXEL)"、 "DOCETAXEL (MCKESSON CORPOR"、 "DOCETAXEL (SAGENT PHARMACE"、 "DOCETAXEL (SANDOZ INC.)"、 "DOCETAXEL (Sanofi-Aventis)"、 "DOCETAXEL 10 MG/ML 16 ML V"、 "DOCETAXEL 130MG IN 250ML DSW BA"、 "DOCETAXEL 151MG"、 "DOCETAXEL 160 MG/16 ML"、 "DOCETAXEL 170MG"、 "DOCETAXEL 50MG"、 "DOCETAXEL 80MG"、 "DOCETAXEL 80MGX1 NOVA PLUS"、 "DOCETAXEL ACCORD 160 MG/8"、 "DOCETAXEL ACCORD 160 MG/8 ML, SOLUTION ? DILUER POUR PERFUSION"、 "DOCETAXEL ACCORD 160 mg/8 ml, solution a diluer pour perfusion"、 "DOCETAXEL ACCORD 80 MG/4 M"、 "DOCETAXEL ACOORD"、 "DOCETAXEL AUROVITAS"、 "DOCETAXEL AUROVITAS 20 MG/ML"、 "DOCETAXEL CONCENTRATE FOR"、 "DOCETAXEL CONCENTRATE FOR SOLUTION FOR INFUSION"、 "DOCETAXEL EAGLE"、 "DOCETAXEL HIKMA"、 "DOCETAXEL HOSPIRA 10 MG/ML"、 "DOCETAXEL INFUUS"、 "DOCETAXEL INJ 20MG/ML"、 "DOCETAXEL INJ 80MG/4ML"、 "DOCETAXEL INJECTION CONCEN"、 "DOCETAXEL INTRAVENOUS INFUSION 20MG/2ML"、 "DOCETAXEL KABI"、 "DOCETAXEL OMNICARE 20 MG/M"、 "DOCETAXEL OPLOSSING VOOR I"、 "DOCETAXEL Sanofi-Aventis"、 "DOCETAXEL, 195MG"、 "DOCETAXEL- Dr. Reddy's"、 "DOCETAXEL- SANOFI"、 "DOCETAXEL- Sanofi-Aventis"、 "DOCETAXEL-MCKESSON"、 "DOCETAXEL-SAGENT"、 "DOCETAXEL-SANOFI"、 "DOCETAXEL-SANOFI AVENTIS"、 "DOCETERE 120 MG INJECTION"、 "DOCTAXEL"、 "Docetaxel 100mg/m2"、 "Docetaxel Concentrate for solution for infusion"、 "Docetaxel MYLAN"、 "Docetaxel Omnicare"、 "Docetaxel Sanofi-Aventis"、 "HOSPIRA [DOCETAXEL]"、 "ONETAXOTERE 20MG"、 "STRIDOTERE"、 "TADOCEL"、 "Tadocel"、 "XI CUN"、 "docetaxel Actavis"、 "docetaxel Hospira"、 "docetaxel accord"、 "docetaxel hospira"、 "docetaxel omnicare"、 |
| Liposome | "TAXOTERE"、 "DOCETAXEL."、 "DOCETAXEL SANDOZ"、 "DOCETAXEL ACCORD"、 "DOCETAXEL HYDRATE"、 "DOCETAXEL INJECTION"、 "DOCETAXEL INJECTION CONCENTRATE"、 "DOCETAXEL SANOFI"、 "Docetaxel Hospira"、 "Docetaxel accord"、 "Docetaxel Teva"、 "DOCETAXEL ACTAVIS"、 "DOCETAXEL (SANOFI-AVENTIS)"、 "Docetaxel Accord"、 "DOCETAXEL ACCORD HEALTHCARE"、 "DOCETAXEL HOSPIRA"、 "DOCETAXEL INJECTION USP, 20 MG/0.5 ML, SINGLE-DOSE VIAL, TWO-VIAL FORM"、 "DOCETAXEL SANOFI-AVENTIS"、 "DOCETAXEL SUN"、 "Docetaxel Injection USP, 20 mg/0.5 mL, Single-dose Vial, Two-Vial Form"、 "DOCETAXEL AUROVITAS 20 MG/ML CONCENTRADO PARA SOLUCION PARA PERFUSION"、 "DOCETAXEL SAGENT"、 "Docetaxel Injection"、 "AISU [DOCETAXEL]"、 "DOCETAXEL (Manufacturer unknown)"、 "DOCETAXEL (SANOFI)"、 "DOCETAXEL ACCORD 80 MG/ 4 ML CONCENTRATE FOR SOLUTION FOR INFUSION"、 "DOCETAXEL Injection"、 "DOCETAXEL MCKESSON"、 "DOCETAXEL OMNICARE"、 "DOCETAXEL SANOFI AVENTIS"、 "DOCETAXEL?ACCORD"、 "DOXETAXEL"、 "Docetaxel Aurovitas"、 "Duopafei"、 "COMPARATOR DOCETAXEL"、 "DOCELIBBS"、 "DOCETAXEL (7394A)"、 "DOCETAXEL (Sanofi-Aventis)"、 "DOCETAXEL ACCORD 160 MG/8 ML, SOLUTION ? DILUER POUR PERFUSION"、 "DOCETAXEL ACCORD 160 mg/8 ml, solution a diluer pour perfusion"、 "DOCETAXEL EAGLE"、 "DOCETAXEL HIKMA"、 "DOCETAXEL PFIZER"、 "DOCETAXEL SUN PHARMA"、 "DOCETAXEL Sanofi-Aventis"、 "DOCETAXEL- Dr. Reddy's"、 "DOCETAXEL- Sanofi-Aventis"、 "DOCETAXEL-MCKESSON"、 "DOCETAXEL-SAGENT"、 "Docetaxel 100mg/m2"、 "Docetaxel Concentrate for solution for infusion"、 "HOSPIRA [DOCETAXEL]"、 "doc?taxel"、 "docetaxel accord"、 |
| Uracil | "URACIL"、 "uracil"、 "Uracil"、 |
| Tegafur | "TEGAFUR"、 "Tegafur"、 "TEGAFUR/TEGAFUR SODIUM"、 "TEGAFUR URACIL"、 "FTORAFUR"、 "tegafur"、 "N1-(2 TETRAHYDROFURYL)-5-FLUOROURACIL (FT-207)"、 "TEGACIN"、 |
| Etoposide | "ETOPOSIDE."、 "ETOPOSIDE (VP-16)"、 "VP-16"、 "VEPESID"、 "ETOPOSIDE (Manufacturer Unknown)"、 "Etoposide (VP-16)"、 "ETOPOSIDE (Manufacturer Unknown"、 "ETOPOSIDE (Manufacturer Un"、 "ETOPOSIDE TEVA"、 "ETOPOSIDE MYLAN"、 "Etoposide (Unknown)"、 "LASTET"、 "VEPESIDE"、 "CELLTOP"、 "ETOPOSID"、 "Etoposide (VP?16)"、 "Etoposide Mylan"、 "ETOPOSIDE INJECTION, USP"、 "CELLTOP 100 MG/5 ML, SOLUT"、 "ETOPOSIDE CAP 50MG"、 "VP?16"、 "CELLTOP 100 MG/5 ML, SOLUTION I"、 "CELLTOP 100 MG/5 ML, SOLUTION INJECTABLE POUR P"、 "ETOPOSIDO (518A)"、 "Etoposide (Watson Laboratories)"、 "ETOPOSIDE INTRAVENOUS INFUSION"、 "Etoposid"、 "ETOPOSIDE (VP?16)"、 "Etoposide Teva"、 "CELLTOP 50 MG, CAPSULE"、 "ETOPOSIDE TEVA - TEVA PHARMA B.V."、 "ETOPOSIDO"、 "TOPOSAR"、 "Vepeside"、 "CELLTOP 25 MG, CAPSULE"、 "Celltop"、 "ETOPOSIDE INJECTION"、 "ETOPOSIDE INTRAVENOUS INFU"、 "Etoposide Capsules, USP"、 "LASTET S"、 "ETOPOSIDE ACCORD"、 "Etoposide Mylan 20 mg/ml, solution ? diluer pou"、 "VP16"、 "etoposide (vp-16)"、 "Etoposide Mylan 20 mg/ml,"、 "Tevaetopo"、 "ETOPOSIDE 50MG MYLAN"、 "ETOPOSIDE-TEVA"、 "VP 16-213"、 "CELLTOP 100 MG/5 ML, SOLUTION INJECTABLE POUR PERFUSION"、 "ETOPOSID EBEWE"、 "ETOPOSIDE"、 "ETOPOSIDE (TEVA)"、 "ETOPOSIDE 100MG/5ML ACCORD, INT"、 "ETOPOSIDE TEVA 20 mg/ml"、 "Etoposide-Teva"、 "VEPSID"、 "Vp-16"、 "ETOPOSIDE /00511902/"、 "ETOPOSIDE SANDOZ"、 "ETOPOSIDE- TEVA"、 "Etoposid Ebewe"、 "ETOPOSIDE 50 MG"、 "ETOPOSIDE INJECTION,USP"、 "ETOPOSIDE-TEVA 20 MG/ML CONCENT"、 "TOPOSIN, CONCENTRAAT VOOR OPLOS"、 "VP 16"、 "etoposid"、 "etoposide Teva"、 "ETOPOSIDE CAPSULES, USP"、 "ETOPOSIDE MERCK"、 "ETOPOSIDE SANDOZ - 20 MG/ML CONCENTRATO PER SOL"、 "ETOPOSIDE SANDOZ ? 20 MG/M"、 "ETOPOSIDE TEVA - 20 MG/ML CONCENTRATO PER SOLUZ"、 "ETOPOSIDO TEVA 20 MG/ML CONCENT"、 "Etoposide Mylan 20 mg/ml, solut"、 "Etoposide mylan"、 "Teva-Etoposide"、 "etoposide (VP-16)"、 "ACCORD ETOPOSIDE"、 "Celltop - capsules molles"、 "ETOPOSIDE (G)"、 "ETOPOSIDE (MYLAN)"、 "ETOPOSIDE 50 MG CAPSULE"、 "ETOPOSIDE INJECTION USP"、 "ETOPOSIDE LIQ IV 20MG/ML"、 "ETOPOSIDE MYLAN 20 MG/ML,"、 "ETOPOSIDE MYLAN 20 mg/ml"、 "ETOPOSIDE MYLAN 20 mg/ml,"、 "ETOPOSIDE MYLAN 20 mg/ml, solution ? diluer pour perfusion"、 "ETOPOSIDE TEVA - TEVA PHARMA B."、 "ETOPOSIDE TEVA 20 MG/ML"、 "ETOPOSIDE TEVA 20 mg/mL"、 "ETOPOSIDE-Teva"、 "ETOPOSIDO TEVAGEN 20 MG/ML CONCENTRATE FOR SOLU"、 "ETOPSIDE MYLAN"、 "EXITOP"、 "Eposin 20 MG/ML"、 "Etoposide Accord"、 "Etoposide Mylan 20 mg/ml, solution ? diluer pour perfusion"、 "Etoposide Sandoz"、 "Lastet"、 "TEVA-ETOPOSIDE"、 "VEPEZIDE"、 "Vepezide"、 "vepeside"、 "BLINDED ETOPOSIDE"、 "CELLTOP 50 MG - CAPSULES MOLLES"、 "CELLTOP 50 mg, capsule"、 "EPOSIDO"、 "EPOSIN 20MG/ML"、 "ESHAP (ETOPOSIDE)"、 "ETHOPOSI [ETOPOSIDE]"、 "ETHOPOSIDE"、 "ETO-GRY (ETOPOSIDE)"、 "ETOMEDAC"、 "ETOPOSIDE (VP -16)"、 "ETOPOSIDE (VP 16)"、 "ETOPOSIDE (VP-16) 2700 MG"、 "ETOPOSIDE (VP-6)"、 "ETOPOSIDE (VP16)"、 "ETOPOSIDE / 50 MG"、 "ETOPOSIDE / 50 MG MYLAN"、 "ETOPOSIDE 20 MG/ML"、 "ETOPOSIDE 20 MG/ML MDV"、 "ETOPOSIDE 223 mg"、 "ETOPOSIDE 50MG"、 "ETOPOSIDE 50MG CAPSULES"、 "ETOPOSIDE BASE"、 "ETOPOSIDE BMS"、 "ETOPOSIDE CAP"、 "ETOPOSIDE CAP 50 MG"、 "ETOPOSIDE CAPSULES 50 MG"、 "ETOPOSIDE EBEWE"、 "ETOPOSIDE ETOPOSIDE"、 "ETOPOSIDE HENGRUI PHARMACE"、 "ETOPOSIDE INJ 20MG/ML"、 "ETOPOSIDE INTRAVENOUS INFUSION 100MG TAIYO"、 "ETOPOSIDE MYLAN 20 mg/ml, solution ? diluer pou"、 "ETOPOSIDE TEVA - 10 ML 20MG/ML"、 "ETOPOSIDE TEVA - 20 MG/ML CONCE"、 "ETOPOSIDE TEVA - 20 MG/ML CONCENTRATO PER SOLU"、 "ETOPOSIDE TEVA - 50 ML 20 MG/ML"、 "ETOPOSIDE TEVA - FLACONE 5 ML 2"、 "ETOPOSIDE TEVA 20 MG/ML, C"、 "ETOPOSIDE TEVA 20 MG/ML, S"、 "ETOPOSIDE TEVA 20 mg/ml, s"、 "ETOPOSIDE TEVA 200 MG"、 "ETOPOSIDE TEVA 200 MG/10 M"、 "ETOPOSIDE TEVA 200 mg/10 m"、 "ETOPOSIDE TEVA 200 mg/10 ml, solution injectable pour perfusion"、 "ETOPOSIDE TEVA 20mg/ml"、 "ETOPOSIDE TEVA 5"、 "ETOPOSIDE TEVA ? 20 MG/ML"、 "ETOPOSIDE Teva"、 "ETOPOSIDE, 20 MG/ML"、 "ETOPOSIDE, 50 MG"、 "ETOPOSIDE, CONCENTRATE FOR SOLU"、 "ETOPOSIDE?TEVA"、 "ETOPOSIDO ACCORD"、 "ETOPOSIDO TEVA 100 MG/5 ML SOLU"、 "ETOPOSIDO TEVA 20 MG/ML"、 "ETOPOSIDO TEVA 20 MG/ML CO"、 "ETOPOSIDO TEVAGEN 20MG/ML CONCENTRADO PARA SOLU"、 "ETOPSOIDE 50MG"、 "ETP (ETOPOSIDE)"、 "EXITOP KONSENTRAT TIL INFUSJONS"、 "Eposin"、 "Etoposide 50 mg"、 "Etoposide Intravenous Infusion 100mg ^TAIYO^"、 "Etoposide VP-16"、 "Etoposide accord"、 "Etoposide."、 "FYTOSID"、 "LASTET INJ. 100MG/5ML"、 "Teva?ETOPOSIDE"、 "VEPESID J"、 "VP 16?213"、 "Vepsid"、 "[PSS GPN] ETOPOSIDE"、 "etoposide Mylan"、 "etoposide VP-16"、 "etoposido"、 |
| Dacarbazine | "DACARBAZINE."、 "DETICENE"、 "DACARBAZINE (Manufacturer unknown)"、 "DACARBAZINE MEDAC"、 "DTIC"、 "DACARBAZINE (Manufacturer unkno"、 "DACARBACIN"、 "DACIN"、 "DACARBAZIN"、 "DACARBAZINE (Manufacturer"、 "DECARBAZINE (DACARBAZINE) INJECTION"、 "DACARBAZINE MEDAC 500 mg"、 "Deticene"、 "BLINDED DACARBAZINE"、 "DACARBACINA"、 "DACARBAZIN TEVA"、 "DACARBAZINA MEDAC"、 "DACARBAZINE (DTIC)"、 "DACARBAZINE MEDAC /00372801/"、 "DACARBAZINE MEDAC /0037280"、 "DACARBAZINE ^MEDAC^"、 "DACIN /00372801/"、 "DTIC-DOME"、 "Dacarbazin"、 "FAULDACAR"、 "dacarbazin"、 |
| Temozolomide | "TEMOZOLOMIDE."、 "TEMODAR"、 "TEMODAL"、 "TEMOZOLOMIDE CAP 100MG"、 "TEMOZOLOMIDE CAP 140MG"、 "TEMOZOLOMIDE 100MG CAP"、 "TEMOZOLOMIDE 140MG CAP"、 "TEMOZOLOMIDE CAP 180MG"、 "TEMOZOLOMIDE CAP 250MG"、 "TEMOZOLOMIDE CAP"、 "TEMOZOLOMIDE 180MG CAP"、 "TEMOZOLOMIDE 20MG CAP"、 "TEMOZOLOMIDE CAP 20MG"、 "TEMOZOLOMIDE 100MG SANDOZ"、 "TEMOZOLOMIDE 100MG"、 "TEMOZOLOMIDE 140MG"、 "Temozolomide Accord"、 "TEMOZOLOMIDE CAP 5MG"、 "TEMOZOLOMIDE 20MG"、 "TEMOZOLOMIDE 250MG CAP TEV"、 "temozolamide"、 "TEMOZOLOMIDE 250MG CAP"、 "TEMOZOLOMIDE 5MG CAP"、 "TEMOZOLOMIDE 5MG"、 "TEMOZOLOMIDE ACCORD"、 "TEMOZOLOMIDE 140MG SANDOZ"、 "Temozolomide SUN"、 "TEMOZOLOMIDE 140MG CAP"、 "TEMOZOLOMIDE CAP"、 "TEMOZOLOMIDE 100 MG"、 "TEMAZOLAMIDE"、 "TEMOZOLOMIDE 100MG CAP"、 "TEMOZOLOMIDE UNKNOWN"、 "TEMOZOLAMIDE TEVA"、 "TEMOZOLMIDE 140MG CAP"、 "TEMOZOLOMIDE 140 MG"、 "TEMOZOLOMIDE 180MG"、 "TEMOZOLOMIDE 100 MG SANDOZ"、 "TEMOZOLOMIDE 100MG CAP"、 "TEMOZOLOMIDE 100mg CAP"、 "TEMOZOLOMIDE 250/5MG TEVA"、 "TEMOZOLOMIDE 250MG SANDOZ"、 "Temozolomide SUN 140 mg hard capsules"、 "temozolomid"、 "TEMOZOLMIDE 5MG CAP"、 "TEMOZOLOMIDE (362856)"、 "TEMOZOLOMIDE 140MG CAP TEVA PHA"、 "TEMOZOLOMIDE 300MG MERCK"、 "TEMOZOLOMIDE, 100 MG"、 "Temozolomide (Unknown)"、 "TEMOZOLMIDE 250MG CAP"、 "TEMOZOLOMIDE 5MG CAP"、 "TEMOZOLOMIDE 100MG CAP TEVA"、 "TEMOZOLOMIDE 100MG SANDOZ INC."、 "TEMOZOLOMIDE 20MG CAP"、 "TEMOZOLOMIDE 250MG"、 "Temozolamide"、 "TEMOZOLAMIDE"、 "TEMOZOLOMIDE 100 TEVA PHARMACEU"、 "TEMOZOLOMIDE 100mg Sandoz"、 "TEMOZOLOMIDE 10MG AMERIGEN"、 "TEMOZOLOMIDE 140 MG CAP"、 "TEMOZOLOMIDE 140mg CAP"、 "TEMOZOLOMIDE 20 MG CAPSULE TEVA"、 "TEMOZOLOMIDE TEVA"、 "Temozolomide SUN 100 mg hard ca"、 "Temozolomide Unknown"、 "TEMAZO L"、 "TEMOZOLMIDE 20MG CAP"、 "TEMOZOLOMI DE 100MG CAP"、 "TEMOZOLOMID"、 "TEMOZOLOMIDE 180MG CAP"、 "TEMOZOLOMIDE 100 Mg"、 "TEMOZOLOMIDE 100MG MERCK"、 "TEMOZOLOMIDE 100MG TEVA"、 "TEMOZOLOMIDE 140MG CAP TEVA PHARMACEUTICALS USA"、 "TEMOZOLOMIDE 140MG CAPS AM"、 "TEMOZOLOMIDE 140MG SANDOZ INC"、 "TEMOZOLOMIDE 140MG UNKNOWN"、 "TEMOZOLOMIDE 180 MG CAPSUL"、 "TEMOZOLOMIDE 180 MG SANDOZ"、 "TEMOZOLOMIDE 180MG SANDOZ INC"、 "TEMOZOLOMIDE 180mg cap"、 "TEMOZOLOMIDE 20 MG"、 "TEMOZOLOMIDE 400 MG TEVA"、 "TEMOZOLOMIDE 75MG/SQ/M"、 "TEMOZOLOMIDE CAP 180mg"、 "TEMOZOLOMIDE CAP 20 MG"、 "TEMOZOLOMIDE CAP 250 MG"、 "TEMOZOLOMIDE CAP 5MG MEDWATCH F"、 "TEMOZOLOMIDE CAP ACCO"、 "TEMOZOLOMIDE SUN"、 "Temozolomide 100mg"、 "COMPARATOR TEMOZOLOMIDE"、 "TEMINTAS"、 "TEMOZOLAMIDE 100MG SANDOZ"、 "TEMOZOLOMIDE 100MG CAP"、 "TEMOZOLOMIDE 180MG CAP"、 "TEMOZOLOMIDE (Manufacturer Unknown)"、 "TEMOZOLOMIDE 100 MG CAPSULE"、 "TEMOZOLOMIDE 100MG CAPSULE"、 "TEMOZOLOMIDE 140 MG CAPSULE TEV"、 "TEMOZOLOMIDE 140 MG SANDOZ"、 "TEMOZOLOMIDE 140 Mg"、 "TEMOZOLOMIDE 140MG CAP"、 "TEMOZOLOMIDE 140mg"、 "TEMOZOLOMIDE 180MG CAP TEVA"、 "TEMOZOLOMIDE 20MG CAPS"、 "TEMOZOLOMIDE 20MG SANDOZ"、 "TEMOZOLOMIDE 20MG SANDOZ INC. A"、 "TEMOZOLOMIDE 20MG TEVA"、 "TEMOZOLOMIDE 250MG ACCORD"、 "TEMOZOLOMIDE 250MG AND 100MG SANDOZ"、 "TEMOZOLOMIDE 250MG CAP TEVA"、 "TEMOZOLOMIDE 250MG OTHER"、 "TEMOZOLOMIDE 270 MG TEVA"、 "TEMOZOLOMIDE 5 MG"、 "TEMOZOLOMIDE CAP 100MG"、 "TEMOZOLOMIDE CAPSULES"、 "TEMOZOLOMIDE MYLAN"、 "TEMOZOLOMIDE SUN 140 MG HA"、 "TEMOZOLOMIDE cap 5mg"、 "TEMOZOLOMIDE++ # 140MG SAN"、 "TEMOZOLOMIDE++ 100MG SANDO"、 "Temozolomide 180mg"、 "Temozolomide SUN 100 mg hard capsules"、 "Temozolomide SUN 140 mg hard ca"、 "Temozolomide, 100 MG"、 "temozolomide 180mg cap"、 "DIQING"、 "MEDTRONOMIC TEMOZOLOMIDE"、 "TEM200MG/M^2 MERCK"、 "TEMDZOLOMIDE, 140 MG"、 "TEMEZOLOMIDE"、 "TEMOMEDAC"、 "TEMOXOLOMIDE"、 "TEMOZLOLAMIDE"、 "TEMOZLOMIDE 5MG CAP"、 "TEMOZO"、 "TEMOZOLAMID RATIOPHARM 100MG"、 "TEMOZOLAMIDE 100MG"、 "TEMOZOLAMIDE 140 MG SANDOZ"、 "TEMOZOLAMIDE 140 MG TEVA"、 "TEMOZOLAMIDE 180 MG SANDOZ"、 "TEMOZOLAMIDE 20 MG"、 "TEMOZOLDMIDE- 180MG"、 "TEMOZOLMIDE"、 "TEMOZOLOMI DE"、 "TEMOZOLOMI DE CAP 20MG"、 "TEMOZOLOMIDA"、 "TEMOZOLOMIDA TEVA 100MG CAPSULAS DURAS, 20 C"、 "TEMOZOLOMIDA TEVA 140MG CAPSULA"、 "TEMOZOLOMIDE 100MG SANDOZ"、 "TEMOZOLOMIDE 140MG CAP"、 "TEMOZOLOMIDE 140mg CAP"、 "TEMOZOLOMIDE 20MG CAP"、 "TEMOZOLOMIDE 250 Mg"、 "TEMOZOLOMIDE 250MG CAP"、 "TEMOZOLOMIDE CAP 140MG"、 "TEMOZOLOMIDE (MANUFACTURER UNKN"、 "TEMOZOLOMIDE (TEMOZOLOMIDE)"、 "TEMOZOLOMIDE , 250 MG"、 "TEMOZOLOMIDE 10 MG AND 5 MG CAP"、 "TEMOZOLOMIDE 100 MG 1452633258"、 "TEMOZOLOMIDE 100 MG SANDOS"、 "TEMOZOLOMIDE 100 MG TEVA PHARMA"、 "TEMOZOLOMIDE 100MG ACCORD"、 "TEMOZOLOMIDE 100MG CAP AME"、 "TEMOZOLOMIDE 100MG cap"、 "TEMOZOLOMIDE 100MG/40MG"、 "TEMOZOLOMIDE 100NG CAP"、 "TEMOZOLOMIDE 100mg CAP"、 "TEMOZOLOMIDE 10MG"、 "TEMOZOLOMIDE 125MG TOTAL DAILY PRESCRIBED DURIN"、 "TEMOZOLOMIDE 140 MG CAP ASCE"、 "TEMOZOLOMIDE 140 MG CAPSULE TEVA"、 "TEMOZOLOMIDE 140, 20MG, 5"、 "TEMOZOLOMIDE 140MG AND 100"、 "TEMOZOLOMIDE 140MG CAO"、 "TEMOZOLOMIDE 140MG CAPSULE"、 "TEMOZOLOMIDE 140MG TEVA"、 "TEMOZOLOMIDE 140mg CAP"、 "TEMOZOLOMIDE 14MG"、 "TEMOZOLOMIDE 14OMG CAP"、 "TEMOZOLOMIDE 180 MG CAP"、 "TEMOZOLOMIDE 180 MG CAP TE"、 "TEMOZOLOMIDE 180 MG CAP TEVA"、 "TEMOZOLOMIDE 180 MG PFIZER"、 "TEMOZOLOMIDE 180M CAP"、 "TEMOZOLOMIDE 180MG CAP AME"、 "TEMOZOLOMIDE 180MG CAP AMERIGEN PHARMACEUTICALS"、 "TEMOZOLOMIDE 180MG CAP TEVA PHA"、 "TEMOZOLOMIDE 180MG CAPSULE"、 "TEMOZOLOMIDE 180mg CAP"、 "TEMOZOLOMIDE 20 MG TEVA USA"、 "TEMOZOLOMIDE 20MG AMERIGEN"、 "TEMOZOLOMIDE 20MG AND 5MG CAP"、 "TEMOZOLOMIDE 20MG NA"、 "TEMOZOLOMIDE 20MG OTHER"、 "TEMOZOLOMIDE 20mg"、 "TEMOZOLOMIDE 25 MG/M2/DAY"、 "TEMOZOLOMIDE 250 MG CAP ASCEND LABORATORIES"、 "TEMOZOLOMIDE 250 MG CAPSUL"、 "TEMOZOLOMIDE 250 MG CAPSULE"、 "TEMOZOLOMIDE 250 MG TEVA PHARMA"、 "TEMOZOLOMIDE 250 mg"、 "TEMOZOLOMIDE 250MG CAP"、 "TEMOZOLOMIDE 250MG ACCORD HEALTHCARE"、 "TEMOZOLOMIDE 25MG CAP TEVA"、 "TEMOZOLOMIDE 40 MG"、 "TEMOZOLOMIDE 40 MG TEVA PHARMAC"、 "TEMOZOLOMIDE 5 MG CAP"、 "TEMOZOLOMIDE 5 MG CAP"、 "TEMOZOLOMIDE 5 MG SANDOZ"、 "TEMOZOLOMIDE 5 Mg"、 "TEMOZOLOMIDE 50 MG/M2/DAY"、 "TEMOZOLOMIDE 50MG/M2/DAY"、 "TEMOZOLOMIDE 5MG ACCORD HE"、 "TEMOZOLOMIDE 5MG SANDOZ"、 "TEMOZOLOMIDE 5MG SANDOZ INC"、 "TEMOZOLOMIDE 5MG SANDOZ INC."、 "TEMOZOLOMIDE 5MG TEVA"、 "TEMOZOLOMIDE 5MGCAPSULE"、 "TEMOZOLOMIDE ACCORD HEALTHCARE"、 "TEMOZOLOMIDE CAP 100MG SANDOZ"、 "TEMOZOLOMIDE CAP 100MG TEVA"、 "TEMOZOLOMIDE CAP 100mg"、 "TEMOZOLOMIDE CAP 140MG CAP"、 "TEMOZOLOMIDE CAP140MG"、 "TEMOZOLOMIDE CAPSULE"、 "TEMOZOLOMIDE CAPSULES (NON-SPECIFIC)"、 "TEMOZOLOMIDE SANDOZ"、 "TEMOZOLOMIDE SUN 100 MG HARD CA"、 "TEMOZOLOMIDE SUN 20 MG HARD CAP"、 "TEMOZOLOMIDE SUN 250 MG HARD CA"、 "TEMOZOLOMIDE SUN 5 MG HARD CAPS"、 "TEMOZOLOMIDE, 100 MG TEVA"、 "TEMOZOLOMIDE, 140 MG"、 "TEMOZOLOMIDE, 140 TEVA"、 "TEMOZOLOMIDE, 140MG"、 "TEMOZOLOMIDE, 20MG"、 "TEMOZOLOMIDE, 250 MG"、 "TEMOZOLOMIDE. 20 MG"、 "TEMOZOMIDE 100 MG TEVA"、 "TENOZOLOMIDE"、 "Temomedac"、 "Temozolmide"、 "Temozolomida Accord"、 "Temozolomide 100 Mg"、 "Temozolomide 100 mg"、 "Temozolomide 140mg Sandoz"、 "Temozolomide 20mg"、 "Temozolomide 250 Mg"、 "Temozolomide 50 mg/m2/day"、 "Temozolomide 5mg"、 "Temozolomide Capsules (non"、 "Temozolomide SUN 20 mg har"、 "Temozolomide SUN 20 mg hard cap"、 "Temozolomide SUN 250 mg hard capsules"、 "Temozolomide SUN 5 mg hard capsules"、 "Temozolomide Teva"、 "Temozolomide, 250 MG"、 "temozolomide 100mg cap"、 "temozolomide 140mg cap"、 "temozolomide sun"、 |
| Pemetrexed | "PEMETREXED."、 "PEMETREXED"、 "Pemetrexed"、 "PEMETREXED SODIUM HYDRATE"、 "PEMETREXED (Manufacturer U"、 "Pemetrexed (Unknown)"、 "PEMETREXED (Manufacturer Unknown)"、 "pemetrexed"、 "PEMETREXED FRESENIUS KABI"、 "PEMETREXED LILLY"、 "Pemetreksed"、 "PEMETREXED /01493902/"、 "PEMETREXED (2944A)"、 "PEMETREXED (MANUFACTURER UNKNOW"、 "PEMETREXED ACTAVIS"、 "Pemetrexed Actavis"、 "Pemetrexed Hospira"、 "PEMETREKSED"、 "PEMETREXED (Manufacturer Unknow"、 "PEMETREXED - ACTAVIS"、 "PEMETREXED ACCORD"、 "PEMETREXED INFUUS"、 "PEMETREXED IV"、 "PEMETREXED LILLY - ELI LILLY NEDERLAND BV"、 "PEMETREXED LILLY ? ELI LIL"、 "PEMETREXED MYLAN"、 "PREMETREXED"、 "Pemetrexed Fresenius Kabi"、 "Pemetrexed Lilly"、 "Premetrexed"、 |
| Gemcitabine | "GEMCITABINE"、 "GEMCITABINE/GEMCITABINE HYDROCHLORIDE"、 "GEMCITABINE/GEMCITABINE HY"、 "GEMCITABINE/GEMCITABINE HYDROCH"、 "GEMCITABINE SANDOZ"、 "GEMCITABINA ACCORD HEALTHC"、 "GEMCITABINE ACTAVIS 40 MG/ML ALCOHOLVRIJ, CONCE"、 "GEMCITABINE MYLAN 40 MG/ML, SOL"、 "GEMCITABINE/GEMCITABINE HYDROCHLORIDE="、 "GEMCITABINE INJECTION SINGLE-USE VIALS 200MG/5."、 "Gemcitabine Sandoz"、 "GEMCITABIN ACCORD"、 "GEMCITABINA ACCORD HEALTHCARE"、 "GEMCITABINE 1GM"、 "GEMCITABINE 900 MG/M2"、 "Gemcitabine 1g/26.3mL Hosp"、 "Gemcitabine 200mg/5.3mL Ho"、 "Gemcitabine Mylan 40 mg/ml"、 |
| Sorafenib | "NEXAVAR"、 "SORAFENIB"、 "SORAFENIB (RAF KINASE INHIBITOR)"、 "SORAFENIB (RAF KINASE INHIBITOR"、 "SORAFENIB (RAF KINASE INHI"、 "Sorafenib"、 "sorafenib"、 "SORAFENIB, 200 MG BAYER"、 "SORANENIB (BAY 43-9006; NEXAVAR)"、 "Nexavar film-coated tablets 200"、 "SOREFENIB (BAY 43-9006"、 "BLINDED SORAFENIB (RAF KINASE I"、 "BLINDED SORAFENIB (RAF KINASE INHIBITOR)"、 "Nexavar film-coated tablets 200mg"、 "SORAFENIB (BAY 43-9006)"、 "SORAFENIB, 200 MG"、 "SORAFENIB, 200MG BAYER"、 "SORAFIB (SORAFENIB)"、 |
| Fluorouracil | "FLUOROURACIL."、 "5-FU"、 "5-FLUOROURACIL"、 "5 FU"、 "FLUOROURACILE"、 "5-FU /00098801/"、 "5-FLUOROURACIL (5-FU)"、 "EFUDEX"、 "5?FU"、 "5?FLUOROURACIL"、 "5-fluorouracil"、 "FLUOROURACILE ACCORD"、 "FLUOROURACIL ACCORD"、 "5-Fluorouracil"、 "5 FLUOROURACIL"、 "Fluorouracil Accord"、 "5-FU /00098801/"、 "FLUOROURACIL 5%"、 "FLUOROURACIL (Manufacturer unknown)"、 "5 FLUORO URACIL"、 "FLUOROURACIL (Manufacturer unkn"、 "5-FU [FLUOROURACIL]"、 "CARAC"、 "5-FLUOROURACIL /00098801/"、 "5-FLUOROURACIL /00098801/"、 "FLOUROURACIL"、 "FLUOROURACIL TOPICAL CREAM"、 "5-FLUOROURACIL [FLUOROURACIL]"、 "FLUOROURACIL 5 FU"、 "5-FLUOROURACIL [FLUOROURAC"、 "FLUOROURACIL TOPICAL CREAM USP"、 "5-fu"、 "FLUOROURACIL (Manufacturer"、 "FLUOROURACILE TEVA"、 "FLUOROPLEX"、 "Efudix"、 "Fluorouracile Pfizer"、 "FLUOROURACILE AHCL"、 "FLUOROURACIL TEVA"、 "TOLAK"、 "ADRUCIL"、 "5-FLUOROURACILE"、 "5?FLUOROURACIL (5?FU)"、 "5FU"、 "Fluorouracile"、 "5-FLU"、 "5?Fluorouracil"、 "EFUDIX"、 "FU"、 "FLUORO URACIL"、 "FLUOROURACIL PFIZER"、 "5?FU [FLUOROURACIL]"、 "5 FLUOROURACIL /00098801/"、 "5?fluorouracil"、 "FLUOROURACIL CREAM 5%"、 "5?FLUOROURACIL [FLUOROURAC"、 "FLUOROURACIL CREAM"、 "FLUOROURACILE PFIZER"、 "5 Fluorouracil"、 "FLUOROURACIL AHCL"、 "Fluorouracile Accord"、 "5-FU HEXAL"、 "5-Fluorouracil (5-FU)"、 "FAULDFLUOR"、 "FLUOROURACIL SANDOZ"、 "FLUOROURACILO"、 "EFUDIX CREAM 5%"、 "5 FLUOROURACIL /00098801/"、 "5 fluorouracil"、 "5-FLUROURACIL"、 "5-fu /00098801/"、 "FLUOROURACILO TEVA"、 "FLUOROURACIL INJECTION, USP"、 "FLUOROURACIL, 5% MILAN"、 "Fluorouracil accord"、 "5-FLUOROURACIL (5-FU)(19893)"、 "5-FLUOROURACIL (F-FU)"、 "5-FU MEDAC"、 "FLUOROURACIL EBEWE"、 "FLUOROURACIL MYLAN"、 "FLUOROURACIL TOPICAL SOLUT"、 "FLUOROURACILE WINTHROP"、 "FLUOROURACILUM"、 "Fluorouracil Pliva 500 mg/"、 "5 fu"、 "5-FU KYOWA"、 "FLUOROURACIL Accord"、 "FLUOROURACIL TOPICAL CREAM USP 5%"、 "FLUOROURACILO (272A)"、 "Fauldfluor"、 "Flourouracil"、 "Fluorouracil AHCL"、 "5-FLUOROURACIL (5-FU) (19893)"、 "5-Fluorouracil Biosyn"、 "5?FU /00098801/"、 "FLUOROURACIL 5% CRE SPE GENERIC FOR EFUDEX 5% C"、 "Fluorouracil (Unknown)"、 "Fluorouracil Pfizer"、 "fluorouracil accord"、 "5- FLUOROURACIL"、 "5-FLUORACILO"、 "5-FLUOROURACIL (5-FU) (198"、 "5-FU ^MEDAC^"、 "EFURIX"、 "FLUOROURACIL"、 "FLUOROURACIL CREAM 5%, 5 G"、 "Fluorouracile pfizer"、 "Flurouracil"、 "fluorouracil Accord"、 "fluorouracile"、 "5-FLUOROURAQCIL (5-FU)"、 "5?FLUORACILO"、 "FLUOROURACIL 5% CREAM"、 "FLUOROURACIL ACCORD /00098801/"、 "FLUOROURACIL CREAM, USP 0."、 "FLUOROURACIL MEDAC"、 "FLUOROURACILE TEVA - 5 G/100 ML SOLUZIONE PER I"、 "FLUOROURACILE TEVA - TEVA ITALI"、 "Fluorouracil Cream, USP 5%"、 "Fluorouracil HS"、 "flourouracil"、 "5-FLUOROURACIl"、 "5-Flourouracil"、 "5-fluorouracil (5-FU)"、 "5-fluorouracil (5-fu)"、 "5?FLUOROURACIL /00098801/"、 "Efudix 50 mg/g Cream"、 "Efudix cream"、 "FLUOROURACIL INJECTION USP"、 "FLUOROURACIL INJECTION, US"、 "FLUOROURACIL UNK"、 "FLUOROURACILE ACCORD 50 mg/ml, solution pour perfusion"、 "FLUOROURACILE TEVA - TEVA ITALIA S.R.L."、 "FLUOROURACILE TEVA 1 G/20"、 "FLUOROURACILE TEVA 5 G/100 ML SOLUZIONE PER INFUSIONE"、 "FLUOROURACILO ACCORD"、 "Fluoroblastin"、 "Fluorouracil 5% cream"、 "Fluorouracile accord"、 "5 FLOURACILO"、 "5- fluorouracil"、 "5-FLUOROURACIL//FLUOROURACIL"、 "5-FLUOROURACYL"、 "5-FLUOURACIL (5-FU)"、 "5?FU MEDAC"、 "EXTERNAL-FLUOROURACIL"、 "FLUOR-URACIL"、 "FLUOROBLASTIN"、 "FLUOROURACIL (5-FU)"、 "FLUOROURACIL 5 PERCENT CRE"、 "FLUOROURACIL ACCORD /00098"、 "FLUOROURACIL CREAM 0.5%"、 "FLUOROURACIL OINTMENT, 5%"、 "FLUOROURACIL TOPICAL SOLUTION U"、 "FLUOROURACILE ACCORD 50 mg"、 "FLUOROURACILE AHCL - 50MG/ML SO"、 "FLUOROURACILE TEVA - 1 G/20 ML"、 "FLUOROURACILE TEVA 5 G/100"、 "FLUOROURACILO SANDOZ"、 "FLUORUROACILE ACCORD"、 "Fauldfluor BI"、 "Fluorouracil 5 fu"、 "RIBOFLUOR"、 "UTORAL"、 "5 FLUOROURACIL [FLUOROURACIL]"、 "5 FLUOROURACILO"、 "5-FLUOROURACIL EBEWE"、 "5-FLUOROURACILO"、 "5-Fluorouracile"、 "5-Flurouracil"、 "5-flourouracil"、 "5-fluorouracile"、 "CIV 5-FU"、 "EFUDIX 5 POUR CENT, CR?ME"、 "Efudix 5% Cream"、 "F-FLUOROURACIL (5-FU)"、 "FLUORO-URACIL"、 "FLUORO-URACILE MEDA"、 "FLUOROUCIL 4,080 MG"、 "FLUOROURACIL /00098802/"、 "FLUOROURACIL 5 TOPICAL CREAM"、 "FLUOROURACIL 5-FU 1800 MG FRESN"、 "FLUOROURACIL 50MG/ML FRESENIUS"、 "FLUOROURACIL CREAM, USP 5%"、 "FLUOROURACIL PLIVA"、 "FLUOROURACIL TOPICAL SOLUTION USP 2%"、 "FLUOROURACILE AHCL - 50MG/ML SOLUZIONE INIETTAB"、 "FLUOROURACILE AHCL - ACCORD HEA"、 "FLUOROURACILE AHCL - ACCORD HEALTHCARE LIMITED"、 "FLUOROURACILE TEVA - 1 G/20ML S"、 "FLUOROURACILE TEVA - 5 G/100 ML"、 "FLUOROURACILE TEVA 5 G/100 ML"、 "FLUOROURACILE TEVA ? TEVA"、 "FLUOROURACILO ACCORD 50 mg"、 "FLUOROURACILO FERRER FARMA"、 "FLUOROURACIS [FLUOROURACIL]"、 "FLUORURACIL"、 "FLUOURACIL"、 "FU5"、 "Fluorouracil Cream"、 "Fluorouracil Cream 5%"、 "Fluorouracil pfizer"、 "Fluorouracile AHCL"、 "Fluouracil"、 "SPEAR FLUOROURACIL CREAM USP 5%"、 "fluorouracile Accord"、 "1-(?-D-5'-DEOSSIRIBOFURANO"、 "5 - Fluorouracil"、 "5 FLOROURACIL"、 "5 FLOUROURACIO"、 "5 FLUOROURACIL (5-FU)"、 "5 FLUOROURACIL [FLUOROURAC"、 "5 fluorouracile"、 "5- FLUOROURACIL EBEWE"、 "5--FLUOROURACIL (FLUOROURACIL)"、 "5-?UOROURACIL"、 "5-?uorouracil"、 "5-FI"、 "5-FLOROURACIL"、 "5-FLOUROURACIL"、 "5-FLOUROURACIL (5-FU)"、 "5-FLUOROURACIL (5/FU)"、 "5-FLUOROURACIL (open label)"、 "5-FLUOROURACIL INJECTION ("、 "5-FLUOROURACIL) (5-FU)"、 "5-FLUOROURCIL (5-FU)"、 "5-FLUOURACIL /00098801/ (FLUORO"、 "5-FU BOLUS"、 "5-FU IV"、 "5-FU Kyowa"、 "5-FU,"、 "5-FU-FLUOROURACIL"、 "5-FU/00098801/"、 "5-Fluoruracil"、 "5-LFUOROURACIL"、 "5-fluoracilo"、 "5-fluorouracil ^biosyn^"、 "5-fluoruracil"、 "50 MG/ML 5-FLUOROURACIL SOLUTIO"、 "5? FLUOROURACIL"、 "5?FLUOROURACIL (5?FU) (198"、 "5?FLUOROURACIL (5?fu)"、 "5?FLUOROURACILE"、 "5?FLUOROURACILO"、 "5?FU HEXAL"、 "5?FU ^MEDAC^"、 "5?Fluorouracil (5?FU)"、 "5?LFUOROURACIL"、 "5?fu"、 "Accord fluorouracil"、 "BENTON"、 "Continuous 5 fu"、 "EFUDIX 5% CREAM"、 "EFUDIX 50 MG/G CREAM"、 "FLOROURACIL 50 MG/ML FRESENIUS"、 "FLOURACYL"、 "FLOUROURACIL CREAM, USP 5%"、 "FLOUROURICIL 5FU"、 "FLUOR?URACIL"、 "FLUORACIL"、 "FLUORACILO"、 "FLUORO URACILE"、 "FLUORO URACILE ICN"、 "FLUORO-URACILE MEDA - MEDA"、 "FLUORO-URACILE MEDA - MEDA PHARMA S.P.A."、 "FLUORORACIL"、 "FLUOROURACIL (272A)"、 "FLUOROURACIL (5-FLUOROURAC"、 "FLUOROURACIL (FOLFOX)"、 "FLUOROURACIL (OCEAN SIDE P"、 "FLUOROURACIL 0.5 %"、 "FLUOROURACIL 5% CREAM USP"、 "FLUOROURACIL 5% TOPICAL SO"、 "FLUOROURACIL 500MG/10ML SD"、 "FLUOROURACIL 5G AMP"、 "FLUOROURACIL ACCORD 50MG/ML"、 "FLUOROURACIL ACCORD HEALTH"、 "FLUOROURACIL AGREEMENT 50"、 "FLUOROURACIL AHCL ACCORD H"、 "FLUOROURACIL CREAM , USP 5"、 "FLUOROURACIL CREAM 5 %"、 "FLUOROURACIL Cream"、 "FLUOROURACIL HEXAL"、 "FLUOROURACIL HIKMA"、 "FLUOROURACIL HOSPIRA (FLUOROURA"、 "FLUOROURACIL INJECTION"、 "FLUOROURACIL SOLUTION FOR"、 "FLUOROURACIL TEVA - 5 G / 100 ML SOLUTION FOR I"、 "FLUOROURACIL TOPICAL CREAM USP,"、 "FLUOROURACIL TOPICAL CREAM, 5%"、 "FLUOROURACIL TOPICAL SOLUTION USP 5%"、 "FLUOROURACIL [5-FLUOROURAC"、 "FLUOROURACIL(FLUOROURACIL)"、 "FLUOROURACIL-GRY 5G/100 ML"、 "FLUOROURACIL-TEVA"、 "FLUOROURACILE ACCORD 50 MG"、 "FLUOROURACILE ACCORD 50 mg/ml"、 "FLUOROURACILE AHCL - 50 MG/ML S"、 "FLUOROURACILE AHCL - ACCOR"、 "FLUOROURACILE AHCL -50MG/ML SOLUZIONE INIETTABI"、 "FLUOROURACILE Accord"、 "FLUOROURACILE MYLAN 50 MG/ML"、 "FLUOROURACILE PFIZER 50 MG"、 "FLUOROURACILE TEVA - 1 G/20 ML SOLUZIONE PER IN"、 "FLUOROURACILE TEVA - 500 MG/10"、 "FLUOROURACILE TEVA - TEVA"、 "FLUOROURACILO ACCORD 50 mg/ml SOLUCION INYECTABLE O PARA PERFUSION EFG"、 "FLUOROURACILO TEVA 50 MG/ML"、 "FLUOROURCIL"、 "FLUOROUTACIL"、 "FLUOURARACIL"、 "FLURACEDYL"、 "FLURACIL"、 "FLUROBLASTIN"、 "FLUROBLASTINE"、 "FLUROURACIL TOPICAL 5 (FU5)"、 "Fluoracil"、 "Fluorouracil (5-FU)"、 "Fluorouracil (5FU)"、 "Fluorouracil (5Fu)"、 "Fluorouracil (Fluorouracil)"、 "Fluorouracil AHCL Accord Healthcare Limited"、 "Fluorouracil Injection, US"、 "Fluorouracil Pliva"、 "Fluorouracil Sandoz"、 "Fluorouracil Solution for"、 "Fluorouracil Teva"、 "Fluorouracil cream 5%"、 "Fluorouracil sandoz"、 "Fluorouracil teva"、 "Fluorouracilo"、 "Fluorouracilo ACCORD"、 "Fluoruracil"、 "Fluoruracile"、 "TN UNSPECIFIED (FLUOROURAC"、 "fluorouracil 5% cream"、 "fluorouracil ACCORD"、 "fluorouracil HS"、 "fluorouracil infuus"、 "fluoruracil"、 |
| Irinotecan | "IRINOTECAN"、 "Irinotecan"、 "Campto"、 "CAMPTO"、 "IRINOTECAN (Manufacturer u"、 "CPT-11"、 "irinotecan"、 "IRINOTECAN (Manufacturer unknown)"、 "IRINOTECAN HOSPIRA"、 "Irinotecan liposome"、 "IRINOTECAN (Manufacturer unknow"、 "Irinotecan (Unknown)"、 "IRINOTECAN HIKMA"、 "Irinotecan Hospira"、 "IRINOTECAN 20 mg/ml CONCEN"、 "IRINOTECAN FRESENIUS"、 "IRINOTECAN 20 mg/ml CONCENTRATE FOR SOLUTION FO"、 "IRINOTECAN MYLAN"、 "IRINOTECAN MEDAC"、 "IRINOTECAN 20 mg/ml CONCENTRATE"、 "IRINOTECAN ACCORD"、 "IRINOTECAN ACTAVIS"、 "irinotecan liposome"、 "NANOLIPOSOMAL IRINOTECAN"、 "CPT?11"、 "IRINOTECAN /01280202/"、 "Irinotecan Concentrate for"、 "Irinotecan Mylan Generics"、 "IRINOTECAN (2719A)"、 "IRINOTECAN ACTAVIS - 20 MG/ML C"、 "IRINOTECAN MYLAN GENERICS"、 "Irinotecan Hosipira"、 "IRINOTECAN INJ 40MG/2ML"、 "IRINOTECAN SDV (5ML/VL)"、 "Irinotecan Actavis"、 "Irinotecan Concentrate for solu"、 "Irinotecan Concentrate for solution for infusion"、 "irinotecan hospira"、 "IRINOTECAN 20MG/ML 5 ML VI"、 "IRINOTECAN ACTAVIS - 20 MG/ML CONCENTRATO PER S"、 "IRINOTECAN AUROBINDO"、 "IRINOTECAN FRESENIUS 20 MG"、 "IRINOTECAN FRESENIUS KABI"、 "IRINOTECAN HIKMA 20MG/ML C"、 "IRINOTECAN HOSPIRA - 20MG/ML CONCENTRATO PER SO"、 "IRINOTECAN Hikma"、 "IRINOTECAN LIPOSOME"、 "IRINOTHECAN"、 "Irinotecan Concentrate for solution for infusio"、 "Irinotecan Kabi"、 "Irinotecan MYLAN"、 "Irinotecan Medac"、 "Irinotecan Solution for in"、 "Irinotecan medac"、 "Irinotecan40"、 "NANOLIPOSONAL IRINOTECAN 5"、 "irinotecan medac"、 "FRESENIUS [IRINOTECAN]"、 "IRENOTECAN"、 "IRINITECAN"、 "IRINOTECAN Aurobindo Concentrate for solution"、 "IRINOTECAN (CPT-11)"、 "IRINOTECAN (MANUFACTURER U"、 "IRINOTECAN (open label)"、 "IRINOTECAN 100MG/5ML SDV I"、 "IRINOTECAN 195MG/500ML"、 "IRINOTECAN ACTAVIS - 20 MG / ML CONCENTRATE FOR"、 "IRINOTECAN ACTAVIS CONCENTRATE"、 "IRINOTECAN ARROW 20 mg/ml, solution for infusio"、 "IRINOTECAN AUROBINDO - AUR"、 "IRINOTECAN AUROBINDO - AUROBINDO PHARMA (ITALIA"、 "IRINOTECAN AUROBINDO -20 MG/ML CONCENTRATO PER"、 "IRINOTECAN AUROBINDO 20 MG"、 "IRINOTECAN AUROBINDO 20 MG/ML CONCENTRATE FOR S"、 "IRINOTECAN AUROBINDO CONCENTRATE FOR SOLUTION F"、 "IRINOTECAN CONCENTRATE FOR SOLUTION FOR INFUSION 20M"、 "IRINOTECAN EBEWE"、 "IRINOTECAN HIKMA - HIKMA F"、 "IRINOTECAN HIKMA - HIKMA FARMACEUTICA (PORTUGAL"、 "IRINOTECAN HOSIPIRA"、 "IRINOTECAN HOSPIRA - 20MG/ML CO"、 "IRINOTECAN INJ 100MG/5ML"、 "IRINOTECAN JIANGSU HENGRUI MEDICINE"、 "IRINOTECAN KABI"、 "IRINOTECAN KABI - 20 MG/ML CONC"、 "IRINOTECAN KABI 20 MG/ML,"、 "IRINOTECAN LIPOSOMA"、 "IRINOTECAN MEDAC 20 MG/ML,"、 "IRINOTECAN PFIZER"、 "IRINOTECAN SOLUTION FOR IN"、 "IRINOTECAN TEVA"、 "IRINOTECAN, 250 MG"、 "IRINOTECAN- AUROBINDO"、 "IRINOTECAN40"、 "IRNIOTECAN"、 "Irenotecan"、 "Irinotecan Accord"、 "Irinotecan Aurobindo 20 mg/ml c"、 "Irinotecan Aurobindo 20 mg/ml concentrate for s"、 "Irinotecan Fresenius"、 "Irinotecan Hikma"、 "Irinotecan Mylan"、 "Irinotecan accord"、 "Irinotecan fresenius"、 "Irinotecan hikma"、 "Irinotecan hospira"、 "LRINOTECAN"、 "MANOLIPOSOMAL IRINOTECAN"、 "NANOLIPOSOMAL IRINOTECAN 5"、 "Nanoliposomal irinotecan"、 "PEGYLATED LIPOSOMAL (IRINO"、 "irinotecan Hospira"、 "nanoliposomal irinotecan"、 |
| Docetaxel* | "TAXOTERE"、 "DOCETAXEL."、 "Docetaxel Accord"、 "DOCETAXEL ACCORD"、 "DOCETAXEL SANDOZ"、 "Docetaxel Hospira"、 "DOCETAXEL HOSPIRA"、 "Docetaxel (Unknown)"、 "DOCETAXEL ACTAVIS"、 "ONETAXOTERE"、 "DOCETAXEL HYDRATE"、 "DOCETAXEL INJECTION"、 "Docetaxel Actavis"、 "DOCETAXEL (Manufacturer unknown)"、 "DOCETAXEL (Manufacturer un"、 "Docetaxel Aurovitas"、 "DOCETAXEL (Manufacturer unknown"、 "DOCETAXEL ^Actavis^"、 "Doxetacel"、 "DOCETAXEL INJECTION CONCENTRATE"、 "DOCETAXEL TEVA"、 "DOCETAXEL SANOFI"、 "DOCETAXEL SANOFI-AVENTIS"、 "DOCETAXOL"、 "CAMITOTIC"、 "DOCETAXEL INTRAVENOUS INFU"、 "DOXITAXEL"、 "Docetaxel accord"、 "Camitotic"、 "DOCETAXEL Accord"、 "DOCETAXEL Injection"、 "DOCETAXEL SUN"、 "DOCETAXEL CONCENTRATE"、 "CAMITOTIC 20 MG/ML"、 "Docetaxel (Actavis)"、 "Docetaxel Teva"、 "DOCETAXEL (SANOFI-AVENTIS)"、 "DOCETAXEL ACCORD HEALTHCAR"、 "EBEDOCE"、 "DOCETAXEL WINTHROP"、 "DOCETAXEL HOSPIRA 10 mg/ml"、 "DOCETAXEL SAGENT"、 "Docetaxel Injection"、 "Docetaxel SUN"、 "docetaxel Accord"、 "COMPARATOR DOCETAXEL"、 "DOCETAXEL MCKESSON"、 "DOCETAXEL TRIHYDRATE"、 "doc?taxel"、 "docetaxel injection"、 "DOCETAXEL 10 MG/ML SANDOZ"、 "DOCETAXEL OMNICARE"、 "DOCETAXEL PFIZER"、 "Docetaxel 80mg/8mL Hospira"、 "Docetaxel Injection non-alcohol formula"、 "DOCETAXEL (Teva product cannot be excluded)"、 "DOCETAXEL ACCORD HEALTHCARE"、 "DOCETAXEL EBEWE"、 "DOCETAXEL INJECTION USP, 20 MG/0.5 ML, SINGLE-DOSE VIAL, TWO-VIAL FORM"、 "DOCETAXEL RATIOPHARM ITALI"、 "DOCETAXEL SANOFI AVENTIS"、 "DOCETAXEL SANOFI?AVENTIS"、 "DOCETAXEL ^ACTAVIS^"、 "DOPAFEI"、 "DOXEL [DOCETAXEL]"、 "Docetaxel Injection USP, 20 mg/0.5 mL, Single-dose Vial, Two-Vial Form"、 "Docetaxel hydrate"、 "TAXOTEL"、 "[PSS GPN] DOCETAXEL"、 "DOCETAXEL (7394A)"、 "DOCETAXEL 10 MG/ML 8 ML VIAL SANDOZ"、 "DOCETAXEL 160 MG-16 ML MDV"、 "DOCETAXEL 160MG/16ML"、 "DOCETAXEL 20MGX3 NOVA PLUS"、 "DOCETAXEL AUROVITAS 20 MG/ML CONCENTRADO PARA SOLUCION PARA PERFUSION"、 "DOCETAXEL CONCENTRAAT VOOR OPLO"、 "DOCETAXEL- SANOFI-AVENTIS"、 "DOXETAXEL"、 "Docetaxel Sandoz"、 "Docetaxel Sanofi?Aventis"、 "AISU [DOCETAXEL]"、 "AOMINGRUN"、 "DOCETAXEL (SANOFI)"、 "DOCETAXEL 10MG/ML 16ML VIA"、 "DOCETAXEL 120MG 250ML"、 "DOCETAXEL 160 MG/16 ML. HO"、 "DOCETAXEL ACCORD 80 MG/ 4 ML CONCENTRATE FOR SOLUTION FOR INFUSION"、 "DOCETAXEL ACCORD HEALTH CA"、 "DOCETAXEL Aurovitas"、 "DOCETAXEL MYLAN"、 "DOCETAXEL NC"、 "DOCETAXEL SUN PHARMA"、 "DOCETAXEL WINTHROP"、 "DOCETAXEL ^HOSPIRA^"、 "DOCETAXEL?ACCORD"、 "Docetaxel 120mg/12mL Hospira"、 "Docetaxel Kabi"、 "Docetaxol"、 "Duopafei"、 "EXTERNAL-DOCETAXEL"、 "TOLNEXA"、 "AO MING RUN"、 "Accord's Docetaxel"、 "BLINDED DOCETAXEL"、 "CHEMOTHERAPY DRUG W/ TAXOTERE"、 "DAXOTEL (DOCETAXEL)"、 "DAXOTEL [DOCETAXEL]"、 "DOC?TAXEL"、 "DOC?TAXEL TRIHYDRAT?"、 "DOCATAXEL"、 "DOCE NC"、 "DOCELIBBS"、 "DOCETAXEL (DOC)"、 "DOCETAXEL (DOCETAXEL)"、 "DOCETAXEL (MCKESSON CORPOR"、 "DOCETAXEL (SAGENT PHARMACE"、 "DOCETAXEL (SANDOZ INC.)"、 "DOCETAXEL (Sanofi-Aventis)"、 "DOCETAXEL 10 MG/ML 16 ML V"、 "DOCETAXEL 130MG IN 250ML DSW BA"、 "DOCETAXEL 151MG"、 "DOCETAXEL 160 MG/16 ML"、 "DOCETAXEL 170MG"、 "DOCETAXEL 50MG"、 "DOCETAXEL 80MG"、 "DOCETAXEL 80MGX1 NOVA PLUS"、 "DOCETAXEL ACCORD 160 MG/8"、 "DOCETAXEL ACCORD 160 MG/8 ML, SOLUTION ? DILUER POUR PERFUSION"、 "DOCETAXEL ACCORD 160 mg/8 ml, solution a diluer pour perfusion"、 "DOCETAXEL ACCORD 80 MG/4 M"、 "DOCETAXEL ACOORD"、 "DOCETAXEL AUROVITAS"、 "DOCETAXEL AUROVITAS 20 MG/ML"、 "DOCETAXEL CONCENTRATE FOR"、 "DOCETAXEL CONCENTRATE FOR SOLUTION FOR INFUSION"、 "DOCETAXEL EAGLE"、 "DOCETAXEL HIKMA"、 "DOCETAXEL HOSPIRA 10 MG/ML"、 "DOCETAXEL INFUUS"、 "DOCETAXEL INJ 20MG/ML"、 "DOCETAXEL INJ 80MG/4ML"、 "DOCETAXEL INJECTION CONCEN"、 "DOCETAXEL INTRAVENOUS INFUSION 20MG/2ML"、 "DOCETAXEL KABI"、 "DOCETAXEL OMNICARE 20 MG/M"、 "DOCETAXEL OPLOSSING VOOR I"、 "DOCETAXEL Sanofi-Aventis"、 "DOCETAXEL, 195MG"、 "DOCETAXEL- Dr. Reddy's"、 "DOCETAXEL- SANOFI"、 "DOCETAXEL- Sanofi-Aventis"、 "DOCETAXEL-MCKESSON"、 "DOCETAXEL-SAGENT"、 "DOCETAXEL-SANOFI"、 "DOCETAXEL-SANOFI AVENTIS"、 "DOCETERE 120 MG INJECTION"、 "DOCTAXEL"、 "Docetaxel 100mg/m2"、 "Docetaxel Concentrate for solution for infusion"、 "Docetaxel MYLAN"、 "Docetaxel Omnicare"、 "Docetaxel Sanofi-Aventis"、 "HOSPIRA [DOCETAXEL]"、 "ONETAXOTERE 20MG"、 "STRIDOTERE"、 "TADOCEL"、 "Tadocel"、 "XI CUN"、 "docetaxel Actavis"、 "docetaxel Hospira"、 "docetaxel accord"、 "docetaxel hospira"、 "docetaxel omnicare"、 |
| Doxorubicin | "DOXORUBICIN"、 "Doxorubicin"、 "doxorubicin"、 "DOXORUBICINE"、 "HYDROXYDAUNORUBICIN"、 "DOXORUBICINE TEVA"、 "DOXORUBICIN (Manufacturer Unknown)"、 "MYOCET"、 "Doxorubicin (Unknown)"、 "Myocet"、 "DOXORUBICIN (Manufacturer"、 "DOXORUBICIN (Manufacturer Unkno"、 "ADRIBLASTINE"、 "PEGYLATED LIPOSOMAL DOXORU"、 "PEGYLATED LIPOSOMAL DOXORUBICIN"、 "Adriblastine"、 "DOXORUBICIN TEVA"、 "DOXORUBICINE (CHLORHYDRATE"、 "DOXORUBICINE ACCORD"、 "DOXORUBIN"、 "DOXORUBICINE /00330901/"、 "Doxorubicine"、 "Hydroxydaunorubicin"、 "Doxorubicin Teva"、 "doxorubicine"、 "Doxorubicin Liposome"、 "DOXORUBICIN LIPOSOMAL"、 "Pegylated liposomal doxorubicin"、 "DOXORUBICINA SOLUTION FOR INFUS"、 "Pegylated Liposomal Doxoru"、 "ADM"、 "DOXORUBICIN INJ 2MG/ML"、 "DOXORUBICINE TEVA 200 mg/100 ml"、 "Pegylated Liposomal Doxorubicin"、 "DOXORUBICIN LIPOSOME"、 "DOXORUBICINE (CHLORHYDRATE DE)"、 "Doxorubicine Teva"、 "ADRIABLASTINA"、 "DOXORUBICIN /00330902/"、 "DOXORUBICIN IV"、 "DOXORUBICINE /00330901/"、 "Doxorubicine teva"、 "Doxorubin"、 "doxorubicin Teva"、 "doxorubicine Teva"、 "myocet"、 "DOXORUBICIN-Teva"、 "Doxorubicin Concentrate for solution for infusion"、 "Doxorubicin-Teva"、 "DOXORUBICIN MYLAN"、 "DOXORUBICIN-TEVA"、 "DOXORUBICINE [DOXORUBICIN]"、 "DOXURUBICIN"、 "Doxorubicin Liposomal"、 "Doxorubicin TEVA"、 "Doxorubicin liposome"、 "Doxorubicin powder for sol"、 "MYOCET - 50 MG POLVERE E ADDITI"、 "MYOCET 50 MG POLVO Y PREMEZCLAS CONCENTRADO PAR"、 "hydroxydaunorubicin"、 "ALDOXORUBICIN"、 "DOXORRUBICINE"、 "DOXORUBICI N"、 "DOXORUBICIN (202A)"、 "DOXORUBICIN INJECTION"、 "DOXORUBICIN/DOXORUBICIN HYDROCH"、 "DOXORUBICINA"、 "DOXORUBICINE TEVA 50 MG/25"、 "DOXORUBICINE TEVA 50 mg/25"、 "DOXORUBICINE TEVA 50 mg/25 ml"、 "Doxorubicin Pliva"、 "Doxorubicine Accord"、 "LIPOSOMAL ENCAPSULATED DOXORUBICIN"、 "MYOCET 50 MG POWDER, DISPERSION AND SOLVENT FOR CONCENTRATE FOR DISPER"、 "TEVA DOXORUBICIN"、 "doxorubicin liposomal"、 "hydroxyl-doxorubicine"、 "pegylated liposomal doxorubicin"、 "ADRIABLASTINE 100 MG"、 "AMYCIN (DOXORUBICIN)"、 "Adriablastine 100 mg"、 "DOCORUBICINE TEVA"、 "DOKSORUBICIN PLIVA"、 "DOXORIBICIN"、 "DOXORUBCIN"、 "DOXORUBICIN Teva 2 MG/ML"、 "DOXORUBICIN (DOXORUBICIN)"、 "DOXORUBICIN (MANUFACTURER UNKNOWN)"、 "DOXORUBICIN (OPEN LABEL)"、 "DOXORUBICIN (R-CHOP)"、 "DOXORUBICIN 0MG"、 "DOXORUBICIN 106MG"、 "DOXORUBICIN 202A"、 "DOXORUBICIN ACTAVIS"、 "DOXORUBICIN EBEWE 2 MG/ML"、 "DOXORUBICIN INJECTIE/INFUUS"、 "DOXORUBICIN PFIZER"、 "DOXORUBICIN PLIVA"、 "DOXORUBICIN SANDOZ"、 "DOXORUBICIN SUSPENSION FOR"、 "DOXORUBICIN, 20MG/10ML PFIZER"、 "DOXORUBICIN, 50MG/25ML FRESNIUS"、 "DOXORUBICIN, 79.6 MG"、 "DOXORUBICIN-LIPOS"、 "DOXORUBICIN?TEVA"、 "DOXORUBICINA /00330901/"、 "DOXORUBICINA CLOROIDRATO"、 "DOXORUBICINA TEVA"、 "DOXORUBICINA TEVA - TEVA ITALIA S.R.L."、 "DOXORUBICINE (NOS)"、 "DOXORUBICINE CHLORHYDRATE TEVA"、 "DOXORUBICINE DAKOTA PHARM"、 "DOXORUBICINE IN LIPOSOMES STUDY DRUG"、 "DOXORUBICINE INFUUS (LIPOSOMAAL)"、 "DOXORUBICINE TEVA 50 mg/25 mL"、 "DOXORUBICINE TEVA 50 mg/25 ml, solution injectable"、 "DOXORUBICINa CLOROIDRATO"、 "DOXORUBIN /2MG/ML"、 "DOXORUBINE"、 "DOXORUBUCIN"、 "Doxorubici n"、 "Doxorubicin Concentrate fo"、 "Doxorubicin Ebewe"、 "Doxorubicin IV"、 "Doxorubicin ebewe"、 "Doxorubicina Cloridrato"、 "Doxorubicine (chlorhydrate"、 "Doxorubicine Teva 50 mg/25 mL"、 "Doxorubicinum"、 "Doxurubicin"、 "HYDROXY-DOXORUBICIN"、 "HYDROXYDAUNORUBICINE"、 "HYDROXYL-DOXORUBICINE"、 "LIPOSOMAL ENCAPSULATED DOXORUBI"、 "MYOCET 50 MG POLVO Y PREME"、 "MYOCET 50 MG POWDER, DISPE"、 "NON-PEGYLATED LIPOSOME-ENCAPSUL"、 "PEGLYLATED LIPOSOMAL DOXORUBICIN"、 "PFIZER DOXORUBICIN"、 "PREVIOUS CHEMOTHERAPY WITH LIPOSOMAL DOXORUBICI"、 "Pegylated liposomal doxoru"、 "doxorrubicin"、 "doxorubicine (chlorhydrate"、 "doxorubicine chlorhydrate"、 |
| Epirubicin | "EPIRUBICIN"、 "Epirubicin"、 "Pharmorubicin"、 "epirubicin"、 "EPIRUBICINE"、 "EPIRUBICIN (Manufacturer unknown)"、 "Epirubicin (Unknown)"、 "EPIRUBICIN (Manufacturer unknow"、 "EPIRUBICIN EBEWE"、 "EPIRUBICIN ACTAVIS"、 "PHARMORUBICIN"、 "epirubicine"、 "Epirubicin MYLAN"、 "EPIRUBICINA AHCL"、 "Epirubicin Actavis"、 "EPIDOXORUBICINA"、 "EPIRUBICIN MYLAN"、 "EPIRUBICINA"、 "EPIRUBICINA TEVA 2 MG/ML S"、 "EPIRRUBICINA"、 "EPIRUBICIN TEVA"、 "EPIRUBICIN (Manufacturer u"、 "EPIRUBICIN actavis"、 "EPIRUBICINA TEVA"、 "EPIRUBICINE MYLAN"、 "Epirubicin Mylan"、 "EPIRUBICIN ^ACTAVIS^"、 "EPIRUBICINE EBEWE"、 "Epirubicine"、 "epirubicine Mylan"、 "EPIADRIAMYCIN"、 "EPIRUBICIN AHCL"、 "EPIRUBICIN ^TEVA^"、 "Epirubicin Teva"、 "Epirubicine Mylan"、 "AI DA SHENG (EPIRUBICIN)"、 "EPIRUBICINE AHCL"、 "Epiadriamycin"、 "Epidoxorubicina"、 "Epirubicin solution for in"、 "epirubicin Actavis"、 "EPIRUBICIN /00699302/"、 "EPIRUBICIN (EPIRUBICIN)"、 "EPIRUBICIN (UNKNOWN) (EPIRUBICI"、 "EPIRUBICIN -UNK MAH"、 "EPIRUBICIN ^Actavis^"、 "EPIRUBICINA TEVA 2 MG/ML SOLUZIONE INIETTABILE O PER INFUSIONE"、 "EPIRUBICINE MYLAN 2 MG/ML, SOLU"、 "EPIRUBICINE MYLAN 2 mg/ml,"、 "EPIRUBICINE WINTHROP"、 "EPIRUBISIN"、 "EPISINDAN 2 MG/ML"、 "Epirrubicina Teva"、 "Epirubicin Sandoz"、 "Epirubicin teva"、 "Epirubicine Mylan 2 mg/ml,"、 "Epirubicine Mylan 2 mg/ml, solu"、 "Epirubicine Mylan 2 mg/ml, solution pour perfus"、 "Episindan"、 "PHARMORUBICIN RD"、 "epirubicina"、 "epirubicine infuus"、 |
| Methotrexate | "METHOTREXATE."、 "METHOTREXATE"、 "METHOTREXATE (TRADE NAME UNKNOWN)"、 "METHOTREXATE (TRADE NAME U"、 "OTREXUP"、 "Methotrexate"、 "METHOTREXATE (TRADE NAME UNKNOW"、 "TECNOMET"、 "METHOTREXAT"、 "METHOTREXATE (Manufacturer Unknown)"、 "METOLATE"、 "METHOTREXATE (Manufacturer Unkn"、 "METHOTREXATE (Manufacturer"、 "LEDERTREXATE"、 "Metoject Pen"、 "Metolate"、 "methotrexate"、 "METHOTREXATE BIODIM"、 "METHOTREXATE INJECTION"、 "Ledertrexate"、 "METHOTREXATO"、 "METHOTREXATE SDV 25MG/ML"、 "Methotrexate Pfizer"、 "APO-METHOTREXATE"、 "METHOTREXATE CAPSULE"、 "METOTREXATO"、 "METHOTREXATE ORION"、 "NORDIMET"、 "EMTHEXATE"、 "METHOTREXATE UNKNOWN GENER"、 "METOTREXATO (418A)"、 "METEX (METHOTREXATE)"、 "METHOTREXATE INJECTION, USP"、 "UNITREXATE"、 "METHOTREXATE 2.5MG"、 "Methotrexat"、 "IMETH"、 "METHOTREXATE 2.5MG TAB"、 "METHOTREXATE TABLETS"、 "Emthexate"、 "METHOTREXATE 50MG/2ML GENERIC"、 "METHOTREXATE [METHOTREXATE]"、 "METHOTREXATE (METHOTREXATE"、 "METHOTREXATE 50MG/2ML"、 "METHOTREXATE 50MG/2ML GENE"、 "METHOTREXAT [METHOTREXATE]"、 "METHOTREXATE UNKNOWN GENERIC"、 "METHOTREXATUM"、 "METHROTREXATE"、 "Methotrexate Orion"、 "APO?METHOTREXATE"、 "METHOTREXATE (METHOTREXATE) INJECTION"、 "METHOTREXATE FOR INJECTION"、 "METHOTREXATE TEVA /00113801/"、 "Methotrexate 2.5mg"、 "METHOTREXATE PFIZER"、 "METOJECT PEN"、 "TECNOMET /00113801/"、 "METHOTREXATE 2.5 MG"、 "METHOTREXATE ORAL"、 "METHOTREXATE UNKNOWN"、 "Mehtotrexate"、 "Namaxir"、 "METHOBLASTIN"、 "METHOTINJ"、 "METHOTREXATE 50MG/2ML MDV,"、 "METHOTREXATE TAB 2.5MG"、 "methotrexat"、 "M.T.X"、 "METHOTREXATE 10 MG/1,33 ML"、 "METHOTREXATE 15mg"、 "METHOTREXATE MYLAN 100 MG/"、 "METHOTREXATE TABS 2.5MG GE"、 "METHOTREXATE Tablets Metho"、 "Tecnomet"、 "Tevametho"、 "XATMEP"、 "Apo-methotrexate"、 "METHOTREXATE (NGX) (METHOTREXATE) UNKNOWN"、 "METHOTREXATE 25 MG/ML 2 ML VIAL"、 "METHOTREXATE 25MG/ML HOSPI"、 "METHOTREXATE 25MG/ML HOSPIRA"、 "METHOTREXATE 25MG/ML MAYNE"、 "METHOTREXATE TABLET"、 "METHOTREXATE TEVA 10 PERCENT"、 "METHOTREXATO /00113801/"、 "METOTREXATO HEXAL"、 "Methotrexate Accord"、 "TREXAN (ESTONIA)"、 "IMETH /00113801/"、 "IMETH [METHOTREXATE]"、 "METHOTREXATE 25MG/ML ACCORD HEA"、 "METHOTREXATE EBEWE"、 "METHOTREXATE FARMOS"、 "METHOTREXATE MYLAN 2.5 MG/"、 "METHOTREXATE PFIZER//METHOTREXA"、 "METHOTREXATE TABLET 10MG"、 "IMETH /00113801/"、 "METHOTRATE"、 "METHOTREXATE 25 MG TEVA"、 "METHOTREXATE 25MG SDV"、 "METHOTREXATE 25MG/ML TEVA"、 "METHOTREXATE 50 MG/2 ML SD"、 "METHOTREXATE INJ 50MG/2ML"、 "METHOTREXATE TAB"、 "METOTREXATO CIPLA"、 "Methotrexate 50mg/2ml"、 "Methotrexate Biodim"、 "ONCOTREX"、 "TREXAMETTE"、 "TREXAN (FINLAND)"、 "HIGH DOSE METHOTREXATE"、 "IMETH (methotrexate)"、 "Imeth 10 mg, comprim? s?ca"、 "METHOBLASTIN /00113801/"、 "METHOTREXATE Injection"、 "METHOTREXATE (MANUFACTURER UNKNOWN)"、 "METHOTREXATE 15 MG/2 ML SO"、 "METHOTREXATE 2.5 MG TABLET"、 "METHOTREXATE 2.5MG (ESI)"、 "METHOTREXATE 50MG/2MG MDV"、 "METHOTREXATE 50MG/2ML MDV MAYNE PHARMACEUTICALS"、 "METHOTREXATE ACCORD"、 "METHOTREXATE FOR INJECTION, USP"、 "METHOTREXATE TAB 2.5mg"、 "METHOTREXATE UNKNOWN UNKNO"、 "METHOTREXATE [METHOTREXATE"、 "METOTREKSAT"、 "METOTREXATO /00113801/"、 "MTCN (METHOTREXATE)"、 "Metex Pen"、 "Methotrexate (Methotrexate)"、 "Methotrexate 2.5 mg"、 "Methotrexate 50mg"、 "Methotrexate Tablet"、 "Methotrexate farmos"、 "Methotrexate injection"、 "Methrotrexate"、 "OTREXUP 25 MG/0.4 ML AUTO-"、 "TECNOMET /00113801/"、 "H_METHOTREXATE"、 "IMETH 10 mg, comprim? s?ca"、 "IMUTREX"、 "METHOTEXATE"、 "METHOTREXAAT INJVLST 10MG/ML"、 "METHOTREXATE (418A)"、 "METHOTREXATE 2,5 MG COMPRE"、 "METHOTREXATE 2.5"、 "METHOTREXATE 2.5MG DAVA PHARM"、 "METHOTREXATE 2.5MG TABLETS"、 "METHOTREXATE 200MG"、 "METHOTREXATE 25 MG FRESENIUS KA"、 "METHOTREXATE 25MG HOSPIRA"、 "METHOTREXATE 25MG/ML 2 ML MDV H"、 "METHOTREXATE 25MG/ML 2ML VIALS"、 "METHOTREXATE 50 MG PER 2 ML SDV"、 "METHOTREXATE 50MG MDV"、 "METHOTREXATE 50MG/2ML HOSPIRA WORLDWIDE, INC."、 "METHOTREXATE 50MG/2ML SDV INJ"、 "METHOTREXATE 50mg/2ml GENE"、 "METHOTREXATE ? UNKNOWN"、 "METHOTREXATE INJECTION, US"、 "METHOTREXATE MDV"、 "METHOTREXATE MDV (2 ML/VIAL) 25MG/ML HOSPIRA"、 "METHOTREXATE MDV,"、 "METHOTREXATE TABLETS USP"、 "METHOTREXATE TEVA"、 "METHOTREXATE UNKNOWN UNKNOWN"、 "METHOTREXATE(MANUFACTURER UNKNOWN)"、 "METHOTRXATE TABLET"、 "METOTREXATO /00113801/"、 "MTX (METHOTREXATE)"、 "Methotrexate 2.5 milligram tabl"、 "Methotrexate Injection"、 "Methotrexate Tablets"、 "NAMAXIR"、 "NORDIMET 15 mg, solution i"、 "REUMATREX"、 "RHEUMATOID ARTHRITIS (METHOTREXATE)"、 "TEVA METHOTREXATE"、 "methotrexate 2.5 mg"、 "5-METHOTREXATE"、 "Apo-Methotrexate"、 "EBETREXAT (METHOTREXATE)"、 "EBETREXAT [METHOTREXATE]"、 "ERVEMIN"、 "Ervemin"、 "Fauldexato"、 "HD METHOTREXATE"、 "HD-MTX"、 "H_Methotrexate"、 "IMETH 10 MG, COMPRIM? S?CA"、 "IMETH 10 MG, COMPRIME SECA"、 "IMETH 10 MG, SCORED TABLET"、 "IMETH 10 MG,SCORED TABLET"、 "IMETH 10 mg, comprime seca"、 "IT METHOTREXATE"、 "Imeth (methotrexate)"、 "MEHTOTREXATE"、 "MEHTOTREXTE"、 "METHOBLASTIN /00113801/"、 "METHOTREAXATE"、 "METHOTREXAAT 7,5 MG"、 "METHOTREXAAT INJECTIEVLOEI"、 "METHOTREXAAT INJVLST 10MG/ML"、 "METHOTREXAAT INJVLST 2,5MG/ML"、 "METHOTREXAAT PCH"、 "METHOTREXAAT PCH TABLET 2."、 "METHOTREXAAT TABLET 2.5MG"、 "METHOTREXANT"、 "METHOTREXAT FARMOS"、 "METHOTREXAT SANDOZ /00113801/"、 "METHOTREXAT TABLET 10MG"、 "METHOTREXATE 50MG MDV"、 "METHOTREXATE 2.5 MG TAB"、 "METHOTREXATE 2.5 mg"、 "METHOTREXATE 25MG/ML GENE"、 "METHOTREXATE 50MG/2ML GEN"、 "METHOTREXATE (740)"、 "METHOTREXATE (MERCK)"、 "METHOTREXATE - PFIZER ITALIA S.R.L."、 "METHOTREXATE 10 MG TABLET"、 "METHOTREXATE 1000MG"、 "METHOTREXATE 12.5MG MAYNE PHARMACEUTICALS"、 "METHOTREXATE 15MG RESUVO"、 "METHOTREXATE 2 MG"、 "METHOTREXATE 2 mg"、 "METHOTREXATE 2.5 MG 6 TABS Q WE"、 "METHOTREXATE 2.5 MG COMPRI"、 "METHOTREXATE 2.5 MG TAB"、 "METHOTREXATE 2.5 MG TABLETS"、 "METHOTREXATE 2.5M GENERIC"、 "METHOTREXATE 2.5MG MEDAC PHARMA"、 "METHOTREXATE 2.5MG TAB MYLAN"、 "METHOTREXATE 2.5MG TABLET QUALI"、 "METHOTREXATE 2.5MG TABLETS, 2.5"、 "METHOTREXATE 2.5MG UNKNOWN"、 "METHOTREXATE 20MG APP"、 "METHOTREXATE 20MG/ML TEVA"、 "METHOTREXATE 225 MG"、 "METHOTREXATE 25MG ACCORD"、 "METHOTREXATE 25MG/1ML"、 "METHOTREXATE 25MG/ML GENERIC"、 "METHOTREXATE 25MG/ML TEVA PHARM"、 "METHOTREXATE 25MG/ML TEVA PHARMACEUTICALS, USA"、 "METHOTREXATE 25MGML MAYNE PHARM"、 "METHOTREXATE 265MG"、 "METHOTREXATE 30 MG"、 "METHOTREXATE 36 MG"、 "METHOTREXATE 45 mg"、 "METHOTREXATE 50 MG PER 2 ML TEV"、 "METHOTREXATE 50/2ML MDV"、 "METHOTREXATE 50MG"、 "METHOTREXATE 50MG SICOR"、 "METHOTREXATE 50MG-2ML MDV HOSPIRA"、 "METHOTREXATE 50MG/2.4ML"、 "METHOTREXATE 50MG/2ML HOSPIRA W"、 "METHOTREXATE 50MG/2ML MDV"、 "METHOTREXATE 50MG/2ML SDV"、 "METHOTREXATE 50MG/2MLMDV,"、 "METHOTREXATE 6 MG"、 "METHOTREXATE ACTAVIS SOLUTION FOR INJECTION/INF"、 "METHOTREXATE BIODIM 25 mg/"、 "METHOTREXATE DINATRIUM"、 "METHOTREXATE HS"、 "METHOTREXATE INJECTABLE, 50 2ML"、 "METHOTREXATE INJECTION METHOTREXATE"、 "METHOTREXATE INJECTION USP 50MG/2ML AND 500MG/2"、 "METHOTREXATE INJECTION, USP 50MG/2ML ISOTONIC L"、 "METHOTREXATE Injection Met"、 "METHOTREXATE LEDERLE"、 "METHOTREXATE MDV (2ML/VL) 25MG/ML HOSPIRA WORLD"、 "METHOTREXATE MDV 25MG/ML NA"、 "METHOTREXATE MDV 25MG/ML PFIZER"、 "METHOTREXATE MDV 50mg/2ml"、 "METHOTREXATE MYLAN 100 MG/ML, SOLUTION FOR INJECTION"、 "METHOTREXATE MYLAN 100 MG/ML, SOLUTION INJECTABLE"、 "METHOTREXATE MYLAN 100 mg/ml"、 "METHOTREXATE MYLAN 2.5 MG/ML, SOLUTION FOR INJE"、 "METHOTREXATE MYLAN 2.5 MG/ML, SOLUTION FOR INJECTION"、 "METHOTREXATE PIZER"、 "METHOTREXATE PO"、 "METHOTREXATE PRESERVATIVE FREE"、 "METHOTREXATE REMEDICA//MET"、 "METHOTREXATE SDV 25/ML"、 "METHOTREXATE SDV 25MG/ML TEVA"、 "METHOTREXATE SOLUTION FOR INJECTION/INFUSION"、 "METHOTREXATE TAB 2.5 GENER"、 "METHOTREXATE TABLET 25MG"、 "METHOTREXATE TABLETS methotrex"、 "METHOTREXATE TABLETS 2.5MG"、 "METHOTREXATE TABLETS METHOTREXA"、 "METHOTREXATE TABS 2.5MG GENERIC"、 "METHOTREXATE TABS 2.5mg G"、 "METHOTREXATE TEVA /00113801/"、 "METHOTREXATE TEVA 10 % (5 g/50 mL)"、 "METHOTREXATE TEVA 2,5 POUR CENT (50 MG/2 ML)"、 "METHOTREXATE TEVA [METHOTR"、 "METHOTREXATE Tablet"、 "METHOTREXATE UNK UNK"、 "METHOTREXATE UNKOWN GENERI"、 "METHOTREXATE WYETH"、 "METHOTREXATE++#"、 "METHOTREXATE, 2 ML"、 "METHOTREXATE, 2.5 MG"、 "METHOTREXATE-ORAL"、 "METHOTREXATE-TEVA"、 "METHOTREXATE/2.5MG"、 "METHOTREXTE"、 "METHREXX"、 "METHROTREXATE 2.5 MG TABLE"、 "METOJECT [METHOTREXATE]"、 "METOTRESSATO TEVA - TEVA ITALIA"、 "METOTREXATO TEVA 100 MG/ML"、 "METOTREXATO//METHOTREXATE"、 "METROTEXATE"、 "MTX 00113801/"、 "MTX 00113801/"、 "MTX(METHOTREXATE)"、 "Methatrexate"、 "Methotrexate 12 mg"、 "Methotrexate 12.5 mg"、 "Methotrexate 2.5"、 "Methotrexate 2.5 mg tablet"、 "Methotrexate 2.5mg Tablets"、 "Methotrexate 2.5mg tab"、 "Methotrexate 50mg/2mL"、 "Methotrexate BIODIM"、 "Methotrexate Farmos"、 "Methotrexate Injection, US"、 "Methotrexate Injection, USP"、 "Methotrexate Pliva"、 "Methotrexate Tab"、 "Methotrexate Tab 2.5mg"、 "Methotrexate Unknown"、 "Methotrexate biodim"、 "Methotrexate orion"、 "Methotrexate tab"、 "Methotrexate tab 2.5MG"、 "Methotrexate tablet"、 "Methotrexate tablets"、 "NORDIMET 15 MG, SOLUTION I"、 "NOVATREX /00113801/ 2,5 MG"、 "Nordimet"、 "Pfizer-Methotrexate"、 "RATIO-METHOTREXATE"、 "REUMAFLEX 50 MG/ML (METHOTREXAT"、 "TEVAMETHO"、 "TEVAMETHO 25MG"、 "[PSS GPN] METHOTREXATE"、 "methotexate"、 "methotrexate 2.5mg"、 "methotrexate 50mg/2ml"、 "methotrexate Biodim"、 "methrotrexate"、 |
| Pirarubicin | "PIRARUBICIN"、 "PINORUBIN"、 "Pirarubicin"、 "pirarubicin"、 "THEPRUBICINE"、 "PIRARUBICINE"、 "TETRAHYDROPYRANYL ADRIAMYCIN"、 "THP-ADRIAMYCIN"、 "PINORUBIN /00963101/"、 "PINORUBIN /00963101/"、 "PIRARUBICIN/PIRARUBICIN HYDROCH"、 "Pinorubin"、 "THP-adriamycin"、 |
| Ramosetron | "NASEA"、 "IRRIBOW"、 "RAMOSETRON HYDROCHLORIDE"、 "RAMOSETRON"、 "Nasea"、 "NASEA OD"、 "Ramosetron hydrochloride"、 "Ramosetron Hydrochloride"、 "Irribow"、 "Nasea OD"、 "RAMOSETRON HCL"、 "Ramosetron"、 "ramosetron"、 "ramosetron hydrochloride"、 |
| Mitomycin | "MITOMYCIN."、 "MITOMYCIN C"、 "AMETYCINE"、 "MITOMYCINE"、 "MITOMYCIN INJ 40MG"、 "MITOMICINA C"、 "MITOMYCIN, LYOPHILIZED WELLS PH"、 "MUTAMYCIN"、 "Ametycine"、 "Mitomycin (Unknown)"、 "MITOMYCINE C"、 "MMC, KYOWA"、 "RECALL- MITOMYCIN 40MG"、 "MITMYCIN INJ 40MG"、 "MITOMYCIN - C"、 "MITOMYCIN 20MG SDV"、 "MITOMYCIN 40 MG ACCORD HEALTHCARE"、 "MITOMYCIN ACCORD"、 "MITOMYCIN C KYOWA 10 MG PO"、 "MITOMYCIN INJ 40MG"、 "MITOMYCIN INJ 20 MG"、 "MITOMYCIN [MITOMYCINE C]"、 "MYTOMYCIN"、 "Mitomycin Accord"、 |
| Bleomycin | "BLEOMYCIN"、 "BLEOMYCIN SULFATE"、 "BLEOMYCINE"、 "BLEOMYCIN SULPHATE"、 "BLEOMYCINE BELLON"、 "BLEOMYCIN ^BAXTER^"、 "BLEOMYCINE BELLON 15 mg"、 "bleomycin sulfate"、 "BLEOCIN /00183901/"、 "BLEOMYCIN INJ 15U"、 "BLEOMYCINE BELLON 15 mg, p"、 "BLEOPRIM"、 "Bleomycin sulfate"、 "BLEOCIN"、 "BLEOMYCIN SULPHATE FOR INJECTION USP"、 "Bleomycine Bellon"、 "BLEOMICINA"、 "BLEOMYCINE /00183901/"、 "BLENOXANE"、 "BLEOMYCINE BELLION 15 MG,"、 "BLEOMYCINE BELLON 15 mg, poudre pour solution injectable"、 "BLEO INJ 5MG"、 "BLEOCEL"、 "BLEOMYCIN (BLEOMYCIN SULFA"、 "BLEOMYCIN BELLON"、 "BLEOMYCIN BELLON 15 MG POW"、 "BLEOMYCIN ^BAXTER^ 15 000"、 "BLEOMYCIN ^TEVA^"、 "BLEOMYCIN sulphate"、 "BLEOMYCINE /00183902/"、 "BLEOMYCINE /00183902/"、 "BLEOMYCINE BELLON 15 MG,"、 "BLEOMYCINE BELLON 15 MG, P"、 "BLEOMYCINE BELLON 15 MG,PO"、 "BLEOMYCINE BELLON 15 mg,"、 "BLEOMYCINE [BLEOMYCIN SULF"、 "BLEOMYCINE [BLEOMYCIN SULFATE]"、 "BLEOPRIM - 15 MG POLVERE PER SO"、 "BLEOPRIM - 15 MG POLVERE PER SOLUZIONE INIETTAB"、 "Bleomicina solfato"、 "Bleomycin Sulfate"、 "Bonar"、 "VERRUBLEN"、 "bleomicina"、 "bleomycin sulphate"、 "bleomycine"、 |
| Mitoxantrone | "MITOXANTRONE"、 "MITOXANTRONE HCL"、 "NOVANTRONE"、 "MITOXANTRONE HYDROCHLORIDE."、 "MITOXANTRONE HYDROCHLORIDE"、 "MITOXANTRONE TEVA"、 "ONKOTRONE"、 "MMITOXANTRONE"、 "ELSEP"、 "MITOXANTRONE /00661302/"、 "MITOXANTRONE/MITOXANTRONE"、 "EVOMIXAN"、 "MITOXANTRON"、 "MITOXANTRON /00661301/"、 "MITOXANTRONE /00661302/"、 "MITOXANTRONE SANDOZ 2 MG/D"、 "MITOXANTRONE TEVA 25 MG/12,5 ML, SOLUTION ? DIL"、 "MITOXANTRONE TEVA 25 mg/12"、 "ONKOTRONE INJECTION 2 MG/ML CONCENTRATE FOR SOLUTION FOR INFUSION"、 "ELSEP 2 MG/ML, SOLUTION ? DILUER POUR PERFUSION"、 "MITOXAN"、 "MITOXANTRONE 34 MG"、 "MITOXANTRONE BASE"、 "MITOXANTRONE TEVA 20 MG/10ML"、 "MITOXANTRONE TEVA 25 MG/12,5 ML"、 "MITOXANTRONE TEVA 25 mg/12,5 ml, solution ? dil"、 "MITOXANTRONE/MITOXANTRONE HYDROCHLORIDE"、 "MITOZANTRONE HYDROCHLORIDE"、 "MYTOXANTRONE"、 "Mitoxanthrone"、 "Mitoxantone"、 "ONKOTRON"、 "ONKOTRONE - 10 MG CONCENTRATO PER SOLUZIONE PER"、 "ONKOTRONE ? 10 MG CONCENTR"、 "ONKOTRONE INJECTION 2 MG/M"、 |
| Vindesine | "VINDESINE"、 "Vindesine"、 "VINDESINE/VINDESINE SULFAT"、 "VINDESINE (SULFATE DE)"、 "vindesine"、 "VINDESINA"、 "XIAIKE"、 "VINDESINE/VINDESINE SULFATE"、 "Xiaike"、 "VDS"、 "VINDESIN"、 |
| Vincristine | "VINCRISTINE"、 "Vincristine"、 "vincristine"、 "VINCRISTIN"、 "VINCRISTINE HOSPIRA"、 "VINCRISTINE TEVA"、 "VINCRISTINE (manufacturer unkno"、 "Vincristine (Unknown)"、 "Vincristine Hospira"、 "VINCRISTINA"、 "Vincristin"、 "VinCRIStine"、 "VINCRISTINA TEVA"、 "vincristin"、 "Vincristina Pfizer Italia"、 "LIPOSOMAL VINCRISTINE"、 "VINCRISTINE/VINCRISTINE SU"、 "Vincristine Teva"、 "VINCRISTINE /00078802/"、 "VCR"、 "Vincristina Pfizer"、 "VINCRISTINE (manufacturer"、 "VincrisTINE"、 "Vincristina"、 "VINCRISTINE/VINCRISTINE SULFATE"、 "VINCRISTIN TEVA"、 "VINCRISTINE PFIZER"、 "vincristina"、 "VINCRISTINE (manufacturer unknown)"、 "VINCRISTINE HOSPIRA 2 mg/2 ml"、 "VINCRISTINE LIPOSOMAL"、 "VINCRISTINE TEVA 0.1 POUR CENT (1 mg/1 ml)"、 "vincristine Hospira"、 "VINCRISTINA TEVA - TEVA PHARMA"、 "VINCRISTINA TEVA - TEVA PHARMA B.V."、 "VINCRISTINA TEVA ITALIA - 1 MG/"、 "VINCRISTINE HOSPIRA 2 mg/2"、 "VINCRISTINE SANDOZ"、 "VINCRISTINE TEVA 0,1 POUR"、 "VINCRISTINE TEVA 0.1 % (1 MG/ 1"、 "Vincristina Teva"、 "Vincristine Pfizer"、 "Vincristine teva"、 "Vincristine-Teva"、 "LIPOSOMAL VINCRISTINE 2.04"、 "VINCRISTINA PFIZER"、 "VINCRISTINA TEVA ITALIA"、 "VINCRISTINE /00078802/"、 "VINCRISTINE 2MG/2ML HOSPIRA"、 "VINCRISTINE HOSPIRA 2 MG/ 2 ML"、 "VINCRISTINE PHARMACHEMIE"、 "VINCRISTINE TEVA 0.1 POUR CENT"、 "Vincristina TEVA"、 "liposomal vincristine"、 "vincristine Teva"、 "BLINDED Vincristine"、 "Liposomal Vincristine"、 "Liposomal vincristine"、 "VICRISTINE"、 "VINCRISTIN (R-CHOP)"、 "VINCRISTIN-TEVA"、 "VINCRISTINA TEVA ? TEVA PH"、 "VINCRISTINA TEVA ITALIA 1"、 "VINCRISTINA TEVA ITALIA ^1 MG/M"、 "VINCRISTINE (809A)"、 "VINCRISTINE (809SU)"、 "VINCRISTINE (LIPOSOMAL)"、 "VINCRISTINE (VINCRISTINE)"、 "VINCRISTINE - TEVA"、 "VINCRISTINE -TEVA"、 "VINCRISTINE 2 MG/2ML HOSPIRA"、 "VINCRISTINE 2MG/2ML SOLN"、 "VINCRISTINE HOSPIRA 2 mg/2ml"、 "VINCRISTINE INJ 1MG/ML"、 "VINCRISTINE INJECTIE/INFUU"、 "VINCRISTINE MAYNE"、 "VINCRISTINE PDR V INJVLST 1MG"、 "VINCRISTINE SANDOZ 1 MG/ML"、 "VINCRISTINE SANDOZ 1 mg/ml, solution injectable"、 "VINCRISTINE TEVA 0.1 % (1 mg/1 ml)"、 "VINCRISTINE TEVA 0.1 percent (1mg/1 mL)"、 "VINCRISTINE TEVA 0.1% (1 mg/1ml"、 "VINCRISTINE ^HOSPIRA^"、 "VINCRISTINE, 1 MG/1ML"、 "VINCRISTINE-TEVA"、 "Vincrisin 2 mg"、 "Vincristine Pharmachemie"、 "Vincristine TEVA"、 "Vincristine hospira"、 "Vincristine liposomal"、 "vincristine HOSPIRA 2 mg/2 ml"、 "vincristine teva"、 |
| Idarubicin | "IDARUBICIN"、 "Idarubicin"、 "Zavedos"、 "idarubicin"、 "ZAVEDOS"、 "IDARUBICINE"、 "EXTERNAL-IDARUBICIN"、 "idarubicine"、 "IDARUBICIN COMP_IDA+"、 "COMPARATOR IDARUBICIN"、 "IDARUBICINA"、 "IDARUBICINA (2550A)"、 "Idarubicine"、 "IDARUBICIN (IDA)"、 "IDARUBICINA /00830301/"、 "IDARUBICINE MYLAN"、 "CHLORHYDRATE D'IDARUBICINE"、 "IDARABUCIN"、 "IDARRUBICINA"、 "IDARUBICIN MYLAN"、 "IDARUBICIN PFIZER"、 "ZAVEDOS 10 MG/10 ML SOLUZIONE INIETTABILE PER USO ENDOVENOSO"、 |
| Cyclophosphamide | "CYCLOPHOSPHAMIDE."、 "NEOSAR"、 "CYTOXAN"、 "ENDOXAN"、 "PROCYTOX"、 "ENDOXAN BAXTER"、 "CYCLOPHOSPHAMID"、 "CYCLOPHOSPHAMIDE/CYCLOPHOS"、 "GENOXAL"、 "CYCLOPHOSPHAMIDE/CYCLOPHOSPHAMIDE MONOHYDRATE"、 "CYCLOPHOSPHAMIDE INJECTION 1G"、 "CYCLOPHOSPHAMIDE FOR INJECTION,"、 "ENDOXAN 1g"、 "PROCYTOX (CYCLOPHOSPHAMIDE) 2000MG/VIAL"、 "Endoxan"、 "SENDOXAN"、 "CYCLOPHOSPHAMIDE MONOHYDRATE"、 "ENDOXAN /00021101/"、 "Endoxan 1000 mg, poudre po"、 "CICLOFOSFAMIDA"、 "CYCLOPHOSPHAMIDE/CYCLOPHOSPHAMI"、 "ENDOXAN /00021101/"、 "CYCLOPHOSPHAMIDE FOR INJECTION, USP"、 "PROCYTOX (CYCLOPHOSPHAMIDE) 200"、 "ENDOXAN 1000 MG, POUDRE POUR SO"、 "CYCLOPHOSPHAMIDE (Manufacturer"、 "ENDOXAN 1000 MG, POUDRE POUR SOLUTION INJECTABL"、 "PROCYTOX (CYCLOPHOSPHAMIDE"、 "CYCLOPHOSPHAMIDE MONOHYDRA"、 "GENUXAL"、 "Endoxan 1000 mg, poudre pour solutioninjectable"、 "ENDOXAN-BAXTER"、 "Endoxan Lyophilisat"、 "ENDOXANA"、 "CYCLOPHOSPHAMIDE INJECTION"、 "ENDOXAN /00021102/"、 "Cyclophosphamid"、 "ENDOXAN-1G"、 "CYCLOPHOSPHAMIDE FOR INJEC"、 "Procytox"、 "ENDOXAN 50 MG, COMPRIM? ENROB?"、 "ENDOXAN 1G POR OLDATOS INJ"、 "CICLOFOSFAMIDA (120A)"、 "CYCLOPHOSPHAMIDE CAP 50MG"、 "CYCLOPHOSPHAMIDE HYDRATE"、 "ENDOXAN 1 G"、 "Cyclophosphamide (Unknown)"、 "ENDOXAN 1G"、 "ENDOXAN [CYCLOPHOSPHAMIDE]"、 "CYCLOPHOSPHAMIDE SANDOZ"、 "Ciclofosfamida"、 "ENDOXAN 1000 MG - POWDER F"、 "Endoxan 500mg inj. (cyclophosph"、 "endoxan"、 "ENDOXAN FOR INJ IG"、 "cyclophosphamid"、 "CYCLOPHOSPHAMIDE 50MG ROXANE"、 "Endoxan 1 g. Trockensubstanz zur intraven?sen Infusion"、 "Endoxan 50 mg, comprim? en"、 "Cyclophosphamide for Injection USP, 500 mg, 1 g and 2 g per Single-dos"、 "CYCLOPHOSPHAMIDE CAP 50 MG"、 "ENDOXAN /00021102/"、 "ENDOXAN I.V., POEDER VOOR"、 "PROCYTOX 2000MG/VIAL"、 "ENDOXAN [CYCLOPHOSPHAMIDE"、 "EXTERNAL-CYCLOPHOSPHAMIDE"、 "Endoxan 1000 mg, poudre pour so"、 "CYCLOPHOSPHOMIDE"、 "ENDOXAN?BAXTER"、 "Sendoxan"、 "ENDOXAN 1000 MG - POWDER FOR SO"、 "ENDOXAN BAXTER - BAXTER S.P.A."、 "ENDOXAN [CYCLOPHOSPHAMIDE MONOHYDRATE]"、 "PROCYTOX CYCLOPHOSPHAMIDE TAB50MG"、 "CYTOXAN LYOPHILIZED"、 "ENDOKSAN POROSHOK DLJA PRIGOTOV"、 "ENDOXAN 1000 MG - POUDRE P"、 "SENDOXAN 1000 MG PULVER TI"、 "CYCLOPHOSPHAMIDE (Manufact"、 "CYTOXIN"、 "ENDOXAN 1G. IV"、 "ENDOXAN ^BAXTER^ 1G TROCKE"、 "ENDOXAN, DRAG?E"、 "ENDOXANA INJECTION 1 G"、 "Endoxan 1000 mg prasok na"、 "CICLOFOSFAMIDE"、 "CYCLOPHOSPHAMIDE (Manufacturer unknown)"、 "CYCLOPHOSPHAMIDE CAP 25MG"、 "CYCLOPHOSPHAN"、 "Cyclofosfamide"、 "ENDOXAN 1000 MG - POUDRE POUR SOLUTION INJECTABLE"、 "ENDOXAN 1000 MG - POWDER FOR SOLUTION FOR INJECTION"、 "ENDOXAN BAXTER ? BAXTER S."、 "ENDOXAN CYCLOPHOSPHAMIDE 2G (AS"、 "ENDOXAN VIAL 1G"、 "Endoxan 500mg inj. (cyclop"、 "MODIFIED PONTICELLI (CYCLOPHOSPHAMIDE)"、 "SENDOXAN 1000 MG PULVER TIL INJ"、 "SENDOXAN 1000 MG PULVER TIL INJEKSJONSVAESKE, O"、 "CYCLOPHOSHAMIDE"、 "CYCLOPHOSPHAMIDE 50 MG CAP ROXA"、 "CYCLOPHOSPHAMIDE INJ 500MG"、 "CYCLOPHOSPHAMIDUM"、 "ENDOXAN 1 G POWDER FOR SOL"、 "ENDOXAN 1 G. TROCKENSUBSTA"、 "ENDOXAN 1000 mg, poudre pour solutioninjectable"、 "ENDOXAN 1G PRASEK ZA RAZTO"、 "ENDOXAN CYCLOPHOSPHAMIDE 2"、 "Endoxan 50 mg, comprim? enrob?"、 "Endoxan 500mg inj. (cyclophosphamide)"、 "SANDOZ LTD CYCLOPHOSPHAMIDE"、 "SENDOXAN 1000 MG PULVER TILL IN"、 "CYCLOPHOSPHAMIDE (26271)"、 "CYCLOPHOSPHAMIDE TABLETS 50 MG"、 "CYCLOSPHOSPHAMIDE"、 "CYKLOFOSFAMID"、 "Cyclophosphamide Injection 1g"、 "Cyclophosphamide Unknown"、 "Cytoxin"、 "ENDOXAN 1000 mg"、 "ENDOXAN 1G POR OLDATOS INJEKCI?HOZ"、 "ENDOXAN 50 MG APVALKOTAS TABLET"、 "ENDOXAN BAXTER 1 G POLVERE"、 "ENDOXAN I.V., POEDER VOOR OPLOS"、 "ENDOXAN VIALS 1 G"、 "Endoxan 1 g. Trockensubsta"、 "Endoxan 200 mg pulbere pen"、 "Endoxan prasak za otopinu"、 "SANDOZ LTD CYCLOPHOSPHAMID"、 "CYCLO"、 "CYCLOFOSFAMIDE SANDOZ"、 "CYCLOPHASPHAMIDE"、 "CYCLOPHOSHAMIDE (26271)"、 "CYCLOPHOSPHAMIDE 50 MG CAP"、 "CYCLOPHOSPHAMIDE 500MG/M2"、 "CYCLOPHOSPHAMIDE 50MG"、 "CYCLOPHOSPHAMIDE CAP 50mg"、 "CYCLOPHOSPHAMIDE TABLETS 5"、 "CYCLOSPHAMIDE"、 "Cyclophosphamide 50mg"、 "Cyclophosphamide Sandoz"、 "ENDOXAN 1000 MG ? POUDRE P"、 "ENDOXAN 1000 MG ? POWDER F"、 "ENDOXAN 50MG"、 "ENDOXAN BAXTER 1 G POLVERE PER SOLUZIONE INIETTABILE"、 "ENDOXAN I.V., POEDER VOOR OPLOSSING VOOR INJECTIE 200, 500, 750, 1000,"、 "ENDOXAN-P"、 "Endoxan Baxter"、 "Endoxan prasak za otopinu za injekciju 1 g"、 "PROCYTOX 2000 MG/VIAL"、 "PROCYTOX CYCLOPHOSPHAMIDE"、 "CICLOPHOSPHAMIDE"、 "CYCLOPHOSHAMIDE CAP 25MG"、 "CYCLOPHOSPAMIDE"、 "CYCLOPHOSPHAMIDE 25MG"、 "CYCLOPHOSPHAMIDE 50MG ROXANE LA"、 "CYCLOPHOSPHAMIDE SANDOZ LTD"、 "Cyclophophamide"、 "Cyclophoshamide"、 "Cyclophosphamide Dci"、 "Cyclophosphamide Hydrate"、 "ENDOKSAN POROSHOK DLJA PRI"、 "ENDOXAN 1 G."、 "ENDOXAN 1000 MG"、 "ENDOXAN BAXTER - 1 G POLVERE PER SOLUZIONE INIE"、 "ENDOXAN BAXTER - BAXTER S."、 "Endoxan 1000 mg prasok na injekcny roztok"、 "Endoxan Injection"、 "Endoxan baxter"、 "PL 00116/0388 BAXTER HEALTHCARE CYCLOPHOSPHAMID"、 "SENDOXAN 1000 MG PULVER TIL INJEKSJONSV?SKE, OP"、 "ciclopshophamide"、 "BLINDED CYCLOPHOSPHAMIDE"、 "CYCLOFOSFAMIDE"、 "CYCLOPHOSFAMIDE"、 "CYCLOPHOSHAMIDE CAP"、 "CYCLOPHOSHAMIDE CAP 50MG"、 "CYCLOPHOSPHAMI"、 "CYCLOPHOSPHAMID (120A)"、 "CYCLOPHOSPHAMIDE (CYCLOPHOSPHAM"、 "CYCLOPHOSPHAMIDE 50 MG"、 "CYCLOPHOSPHAMIDE 50MG CAP WEST-"、 "CYCLOPHOSPHAMIDE 50MG CAPS"、 "CYCLOPHOSPHAMIDE 50MG WEST-WARD"、 "CYCLOPHOSPHAMIDE DCI"、 "CYCLOPHOSPHAMIDE, 50 MG"、 "CYCLOPOSPHAMIDE (CYCLOPHOSPHAMIDE)"、 "CYCLOXAN"、 "Cyclophasphamide"、 "Cyclophoshphamide"、 "Cyclophosphamide Monohydra"、 "Cyclophosphemide"、 "Cyclophosphomide"、 "ENDOXAN (CYCLOPHOSPHAMIDE"、 "ENDOXAN 1000 MG, LYOPHILISAT PO"、 "ENDOXAN 1000 MG, POUDRE PO"、 "ENDOXAN 1g IV Infuz. Icin"、 "ENDOXAN 50 MG - COATED TAB"、 "ENDOXAN 50 mg, comprim? en"、 "ENDOXAN 50 mg, comprim? enrob?"、 "ENDOXAN 500MG VIAL"、 "ENDOXAN ASTA"、 "ENDOXAN BAXTER - 1 G POLVERE PE"、 "ENDOXAN BAXTER - 500 MG POLVERE"、 "ENDOXAN I.V., POEDER VOOR OPLOSSING VOOR INJECT"、 "ENDOXAN VIALS 1G VIAL"、 "ENDOXAN ^BAXTER^ 1G - TROCKENST"、 "ENDOXAN ^BAXTER^ 1G - TROCKENSTECHAMPULLE"、 "Endoxan 500 mg, poudre pou"、 "GENOXAL GRAGEAS"、 "HYPERFRACTIONATED CYCLOPHOSPHAM"、 "PROCYTOX CYCLOPHOSPHAMIDE TAB50"、 "SENDOXAN 1000 MG INJEKTIOK"、 "SENDOXAN 1000 MG INJEKTIOKUIVA-AINE, LIUOSTA VA"、 "SENDOXAN 50 MG DRAGERAD TABLETT"、 "cyclophosfamide"、 "cyclophosphamide (anhydrou"、 "sendoxan"、 "*CYCLOPHOSPHAMIDE"、 "*Cyclophosphamide"、 "Alkyloxan"、 "BAXTER CYCLOPHOSPHAMIDE"、 "BAXTER HEALTHCARE CYCLOPHOSPHAMIDE INJECTION 1G"、 "CHCLOPHOSPHAMIDE"、 "CICLOFOSFAMIDE BAXTER"、 "CYCHLOPHOSPHAMIDE"、 "CYCLOFOSFAMIDE INJECTIE/INFUUS"、 "CYCLOFOSPHAMIDE"、 "CYCLOHOSPHAMIDE"、 "CYCLOPHOPSHAMIDE"、 "CYCLOPHOSAMIDE"、 "CYCLOPHOSHPHAMIDE"、 "CYCLOPHOSPH 50 MG"、 "CYCLOPHOSPH INJ 500MG"、 "CYCLOPHOSPHAMI 0MG"、 "CYCLOPHOSPHAMI1 DE/CYCLOPHOSPHAMIDE MONOHYDRATE"、 "CYCLOPHOSPHAMID FARMOS"、 "CYCLOPHOSPHAMID SANDOZ"、 "CYCLOPHOSPHAMIDE (26271)"、 "CYCLOPHOSPHAMIDE (120A)"、 "CYCLOPHOSPHAMIDE (NON-ROCHE/NON-COMP.)"、 "CYCLOPHOSPHAMIDE (OPEN LABEL)"、 "CYCLOPHOSPHAMIDE 0 MG"、 "CYCLOPHOSPHAMIDE 1GM SDV"、 "CYCLOPHOSPHAMIDE 50 MG ROXANNE"、 "CYCLOPHOSPHAMIDE 50 MG UNKNOWN"、 "CYCLOPHOSPHAMIDE 50 Mg"、 "CYCLOPHOSPHAMIDE 50MG CAP"、 "CYCLOPHOSPHAMIDE 50MG CAP ROXAN"、 "CYCLOPHOSPHAMIDE 50MG CAPSULES"、 "CYCLOPHOSPHAMIDE 50MG EPOCRATES"、 "CYCLOPHOSPHAMIDE 50MG WEST-WARD PHARMACEUTICALS"、 "CYCLOPHOSPHAMIDE 50MG, CAP ROXA"、 "CYCLOPHOSPHAMIDE 50mg"、 "CYCLOPHOSPHAMIDE 59 MG"、 "CYCLOPHOSPHAMIDE BAXTER HEALTHC"、 "CYCLOPHOSPHAMIDE CAP"、 "CYCLOPHOSPHAMIDE CAP SOMG"、 "CYCLOPHOSPHAMIDE CAPSULE"、 "CYCLOPHOSPHAMIDE CYCLOPHOSPHAMI"、 "CYCLOPHOSPHAMIDE INJ"、 "CYCLOPHOSPHAMIDE INJECTIE/INFUU"、 "CYCLOPHOSPHAMIDE MODIFIED 100MG"、 "CYCLOPHOSPHAMIDE ORION"、 "CYCLOPHOSPHAMIDE UNKNOWN"、 "CYCLOPHOSPHAMIDE( 26271)"、 "CYCLOPHOSPHAMIDE(CYCLOPHOSPHAMIDE)"、 "CYCLOPHOSPHAMIDE/CYCLOPHOSPHAMIDE MONOHYDRATE"、 "CYCLOPHSAPHAMIDE"、 "CYCLOPHSPHAMIDE"、 "CYCLOPSHAMIDE (26271)"、 "CYCLPHOSPHAMIDE FOR INJECT"、 "CYCLPOPHOSPHAMIDE"、 "CYCYLOPHOSHAMIDE"、 "CYCYLOPHOSPHAMIDE"、 "CYKLOFOSFAMID ORION"、 "CYTOPHOSPHAN"、 "CYTOTAXAN (CYCLOPHOSPHAMIDE)"、 "Ciclofosfamide"、 "Cyclophosamide"、 "Cyclophospamide"、 "Cyclophosphami"、 "Cyclophosphamide (R-CHOP)"、 "Cyclophosphamide (anhydrous)"、 "Cyclophosphamide Baxter"、 "Cyclophosphamide hydrate"、 "Cyclophosphamide sandoz"、 "Cyclophsphamide"、 "Cyclosphamide"、 "EMA-CO (CYCLOPHOSPHAMIDE COMPON"、 "ENDOKSAN TABLETKI POKRYTYE SAHA"、 "ENDOXAN /00021101/"、 "ENDOXAN 1000 MG, POUDRE P"、 "ENDOXAN BAXTER"、 "ENDOXAN 1 G MILTELIAI INJE"、 "ENDOXAN 1 G MILTELIAI INJEKCINIAM TIRPALUI"、 "ENDOXAN 100 MG, POUDRE POUR SOL"、 "ENDOXAN 100 mg, poudre pou"、 "ENDOXAN 1000 MG - POUDRE POUR S"、 "ENDOXAN 1000 MG - POUDRE POUR SOLUTION INJECTAB"、 "ENDOXAN 1000 MG - POWDER FOR SOLUTION FOR INJEC"、 "ENDOXAN 1000 MG PRASOK NA"、 "ENDOXAN 1000 MG, LYOPHILIS"、 "ENDOXAN 1000 MG, LYOPHILISAT POUR SOLUTION INJECTABLE"、 "ENDOXAN 1000 MG, POWDER FO"、 "ENDOXAN 1000 mg, lyophilis"、 "ENDOXAN 1000 mg, lyophilisat pour solution injectable"、 "ENDOXAN 1000 mg, powder fo"、 "ENDOXAN 1000MG"、 "ENDOXAN 1000mg"、 "ENDOXAN 1G IV INFUZ. ICIN TOZ ICEREN FLAKON"、 "ENDOXAN 1G POR OLDATOS INJEKCI?"、 "ENDOXAN 1G PRASEK ZA RAZTOPINO"、 "ENDOXAN 1g IV Infuz. Icin Toz Iceren Flakon"、 "ENDOXAN 200 MG PULBERE PENTRU SOLUTIE PERFUZABI"、 "ENDOXAN 2000 MG, LYOPHILISAT PO"、 "ENDOXAN 50 MG - COMPRIM?S ENROB"、 "ENDOXAN 50 MG - COMPRIM?S ENROB?S"、 "ENDOXAN 50 MG DRAJEURI"、 "ENDOXAN 50 MG, COMPRIM? EN"、 "ENDOXAN 50 MG, COMPRIME EN"、 "ENDOXAN 50 MG. DRAJE"、 "ENDOXAN 500 MG - POUDRE POUR SOLUTION INJECTABL"、 "ENDOXAN 500 MG, POUDRE POUR SOL"、 "ENDOXAN 500 MG, POUDRE POUR SOLUTION INJECTABLE"、 "ENDOXAN 500 mg, poudre pou"、 "ENDOXAN 500 mg, poudre pour solution injectable"、 "ENDOXAN 500mg"、 "ENDOXAN BAXTER 50 MG COMPR"、 "ENDOXAN BAXTER S.P.A."、 "ENDOXAN CYCLOPHOSPHAMIDE 1G (AS"、 "ENDOXAN CYCLOPHOSPHAMIDE 2G (AS MONOHYDRATE) PO"、 "ENDOXAN CYCLOPHOSPHAMIDE 500MG"、 "ENDOXAN LYOPHILISAT /00021"、 "ENDOXAN OBLOZENE TABLETE 50 MG"、 "ENDOXAN OMHULDE TABLET, OMHULDE TABLETTEN 50 MG"、 "ENDOXAN PRASAK ZA OTOPINU ZA INJEKCIJU 1 G"、 "ENDOXAN VIAL 200 MG"、 "ENDOXAN VIALS 1G"、 "ENDOXAN ^BAXTER^ 200 MG - TROCKENSTECHAMPULLE"、 "ENDOXAN, 2000MG, POWDER FOR INJ"、 "ENDOXAN, 2000MG, POWDER FOR INJECTION"、 "ENDOXAN//CYCLOPHOSPHAMIDE MONOH"、 "ENDOXANA INJECTION 500 MG"、 "ENDOXANE"、 "Endoxan (Cyclophosphamide"、 "Endoxan 1500 mg"、 "Endoxan 200 mg pulbere pentru solutie perfuzabi"、 "Hyperfractionated Cyclophosphamide"、 "Hyperfractionated cyclopho"、 "PROCYTOX TABLET 50 MG"、 "SANDOZ CYCLOPHOSPHAMIDE"、 "SENDOXAN 100 MG PULVER TIL INJEKSJONSVAESKE, OP"、 "SENDOXAN 1000 MG"、 "SENDOXAN 1000 MG INJEKTIOKUIVA-"、 "SENDOXAN 1000 MG POWDER FOR INJ"、 "SENDOXAN 1000 MG POWDER FOR INJECTION FLUID, SO"、 "SENDOXAN 1000 MG PULVER TILL INJEKTIONSV?TSKA, L?SNING"、 "Sandoz Ltd Cyclophosphamid"、 "Sandoz Ltd Cyclophosphamide"、 "cyclofosfamide"、 "cyclophosphamide (anhydrous)"、 "cyclophosphamide hydrate"、 "cyclophosphamide monohydra"、 "cyclosphosphamide"、 "cyklofosfamid"、 "endoxana"、 "procytox"、 |
| Ifosfamide | "IFOSFAMIDE."、 "HOLOXAN"、 "IFEX"、 "IFOSFAMIDE EG"、 "IFOSFAMIDE (Manufacturer unknown)"、 "IFOSFAMIDE FOR INJECTION"、 "IFOSFAMIDE (Manufacturer unknow"、 "IFOSFAMIDE INJECTION 2G"、 "HOLOXAN 2000 mg, poudre po"、 "IFOMIDE"、 "HOLOXAN 2000 MG, POUDRE POUR US"、 "HOLOXAN 2 G"、 "Holoxan"、 "IFOSFAMIDE (Manufacturer u"、 "Ifosfamide EG"、 "Ifosphamide"、 "HOLOXAN 1000MG INJ (IFOSFAMIDE)"、 "TRONOXAL 1G INYECTABLE"、 "HOLOXAN 2000 MG, POUDRE POUR USAGE PARENT?RAL"、 "EXTERNAL-IFOSFAMIDE"、 "IFOSFAMIDA"、 "IFOSFAMIDE EG 40 mg/ml"、 "HOLOXAN 2000 mg, poudre pour usage parent?ral"、 "IFOSFAMIDA (337A)"、 "Iphosphamide"、 "HOLOXAN 1000 mg, poudre pour solution injectable"、 "HOLOXANE"、 "Holoxani"、 "IPHOSPHAMIDE"、 "HOLOXAN 1000 MG, POUDRE POUR SO"、 "IFOSFAMIDE FOR INJECTION, USP"、 "HOLOXAN (2GM)"、 "HOLOXAN 2000 MG POR OLDATOS INJEKCI?HOZ"、 "HOLOXAN INJ 1GM (30ML VIAL)"、 "HOLOXAN, POEDER VOOR OPLOSSING VOOR INFUSIE"、 "IFOSFAMIDE EG LABO"、 "IFOSFAMIDE, 50 MG/ML TEVA"、 "Ifosamide"、 "HOLOXAN 1000MG INJ (IFOSFA"、 "HOLOXAN 1G"、 "HOLOXAN 2 G IV"、 "HOLOXAN 2000 mg, poudre pour us"、 "HOLOXAN 500MG"、 "HOLOXAN INJEKTIO/INFUUSIOK"、 "HOLOXAN INJEKTIO/INFUUSIOKUIVA-AINE, LIUOSTA VA"、 "HOLOXAN, POEDER VOOR OPLOS"、 "Holoxane"、 "IFOSFAMID"、 "IFOSFAMIDE EG 40 mg/ml, solution pour perfusion"、 "Ifofsamide"、 "Ifosfamid"、 "MITOXANA"、 "PI FU PING"、 "ifosfamide EG"、 "HALOFAXAN"、 "HALOXAN"、 "HOLOXAN (IFOSFAMIDE)"、 "HOLOXAN - 1 G POLVERE PER SOLUZIONE INIETTABILE"、 "HOLOXAN 1000"、 "HOLOXAN 1G INYECTABLE"、 "HOLOXAN 2 g"、 "HOLOXAN 2000 MG POR OLDATO"、 "HOLOXAN 2000 MG PRASOK NA"、 "HOLOXAN 2G"、 "HOLOXAN 2G IV INFUZYON ICIN TOZ ICEREN FLAKON"、 "HOLOXAN 2G PRAEK ZA RAZTOPINO Z"、 "HOLOXAN 2g"、 "HOLOXAN 2g IV Infuzyon Icin Toz Iceren Flakon"、 "HOLOXAN 3 g, lyophilisat p"、 "HOLOXAN BAXTER"、 "HOLOXAN BAXTER S.P.A"、 "HOLOXAN INJ IV (IFOSFAMIDE 1G/1 VIAL)"、 "HOLOXAN INJEKTIO/INFUUSIOKUIVA-"、 "Holoxan 1g"、 "Holoxanum"、 "IFOSAMIDE"、 "IFOSFAMID A-PHARMA"、 "IFOSFAMIDE (G)"、 "IFOSFAMIDE (G)"、 "IFOSFAMIDE 0 MG"、 "IFOSFAMIDE BAXTER"、 "IFOSFAMIDE EG 40 MG/ML, SO"、 "IFOSFAMIDE FOR INJECTION U"、 "IFOSFAMIDE FORIFOSFAMIDE INJECTION, USP"、 "IFOSFAMIDE MYLAN"、 "IFOSFAMIDE, 1 GRAM/ 20 ML LIQUI"、 "IFOSFAMIDE, 2000 MG/M2 LIQUID F"、 "IFOSFAMIDE, 50 MG/ML"、 "IFOSFAMIZE"、 "IFOXFAMIDE"、 "IFSOFAMIDE EG"、 "Ifosfamide Eg"、 "Ifosfamide injection"、 "MITOXANA 2G POWDER FOR STE"、 "ifosamide"、 "ifosfamid"、 "ifosfamide eg"、 |
| Cytarabine | "CYTARABINE."、 "ARACYTINE"、 "ARA-C"、 "Aracytine"、 "CYTARABINE/CYTARABINE HYDR"、 "CYTOSINE ARABINOSIDE"、 "CYTARABINE (Manufacturer Unknown)"、 "CYTARABINE (Manufacturer U"、 "CYLOCIDE"、 "CYTARABINE/CYTARABINE HYDROCHLORIDE/CYTARABINE"、 "Cytosar"、 "CYTARABINE (Manufacturer Unknow"、 "CYTARABINE SANDOZ"、 "CYTARABIN"、 "CYTOSAR"、 "CYTARABINE/CYTARABINE HYDROCHLORIDE/CYTARABINE OCFOSFATE"、 "CYTARABINE INJECTION"、 "CYTARABINE/CYTARABINE HYDROCHLO"、 "DEPOCYT"、 "Ara-C"、 "Aracytin"、 "ARACYTIN"、 "CYTARABINE EBEWE"、 "Citarabina"、 "CYTARABINE KABI"、 "CITARABINA"、 "LIPOSOMAL CYTARABINE"、 "CITARABINA (124A)"、 "CYTARABINE ACCORD"、 "ARA?C"、 "Cytosine arabinoside"、 "cytarabin"、 "DEPOCYTE"、 "Cytarabine (Unknown)"、 "Ara-c"、 "Cylocide"、 "Cytarabin"、 "COMPARATOR CYTARABINE"、 "CYTARABINE EG"、 "EXTERNAL-CYTARABINE"、 "CITARABIN"、 "CYTARABIN FRESENIUS KABI"、 "CITARABINA ACCORD"、 "ARABINOSYLCYTOSINE"、 "Ara?C"、 "CYTARABINE MYLAN"、 "Cytarabine Kabi"、 "Depocyte"、 "CYTARABINE (63878)"、 "CYTOSAR-U"、 "Liposomal cytarabine"、 "Cytarabine Sandoz"、 "ara-c"、 "AraC"、 "CYTARABINE SANDOZ 100 mg/m"、 "cytosine arabinoside"、 "ARA CELL"、 "CITARABINA (THIRD PRODUCTS)"、 "CYTARABINE HOSPIRA"、 "Cytarabine Injection"、 "ALEXAN (CYTARABINE)"、 "ALEXAN /00146201/"、 "ARA-CELL"、 "ARABINOSIDE CYTOSINE"、 "ARAC"、 "Arabinosylcytosine"、 "CYTARABIN ACCORD"、 "CYTARABINE 100 mg/ml"、 "CYTARABINE PFIZER"、 "CYTARABINE SANDOZ 100 mg/ml"、 "[PSS GPN] CYTARABINE"、 "ara-C"、 "ARACYTINE 40 mg, lyophilis"、 "Aracytin CS"、 "CITALOXAN"、 "CITARABINA (CYTARABINE)"、 "CYLOCIDE N"、 "CYTARABINE (SANDOZ)"、 "CYTARABINE ARABINOSIDE"、 "CYTARABINE SANDOZ 100 mg/mL"、 "CYTARABINUM"、 "CYTOSTAR"、 "Cytarabine Accord"、 "Cytarabine EG"、 "Cytarabine Intravenous Inf"、 "Cytarabine sandoz"、 "Cytosine Arabinoside"、 "Cytosine-arabinoside"、 "DEPOCYTE (LIPOSOMAL CYTARABINE)"、 "DepoCyte"、 "FAULDCITA"、 "HIGH DOSE ARA-C"、 "cytarabine Kabi"、 "*CYTARABINE"、 "ALEXAN 100"、 "ARACYTIN - 500 MG/10 ML POLVERE"、 "ARACYTIN CS"、 "ARACYTINE (CYTARABINE)"、 "ARACYTINE 1 G, LYOPHILISAT"、 "ARACYTINE 100 MG"、 "ARACYTINE 100MG"、 "Ara C"、 "Ara-cell"、 "Ara?c"、 "Arabine"、 "BEHENOYL CYTARABINE"、 "CITARABINA HIKMA 2 G/20ML"、 "CITARABINA HIKMA 2 G/20ML SOLUZIONE INIETTABILE"、 "CITARABINE"、 "CYLOCIDE 100MG"、 "CYTABINE"、 "CYTARABAINE INJECTION(CYTA"、 "CYTARABIN KABI"、 "CYTARABINA ACCORD"、 "CYTARABINE (ACCORD)"、 "CYTARABINE (ARA?C)"、 "CYTARABINE (CYTARABINE)"、 "CYTARABINE (EG)"、 "CYTARABINE (HIDAC)"、 "CYTARABINE 0 MG"、 "CYTARABINE 100 MG/ML"、 "CYTARABINE 60 MG"、 "CYTARABINE 70 MG"、 "CYTARABINE 70MG"、 "CYTARABINE 730MG"、 "CYTARABINE ACCORD 100 MG/ML"、 "CYTARABINE CYTARABINE"、 "CYTARABINE FRESENIUS KABI"、 "CYTARABINE INTRAVENOUS INFUSION 1G ^TEVA^"、 "CYTARABINE KABI (ARACYTINE)"、 "CYTARABINE OCPHOSPHATE"、 "CYTARABINE SANDOZ 100 MG/M"、 "CYTARABINE SANDOZ 100 mg/ml, solution injectabl"、 "CYTARBABINE"、 "CYTOSIANARABINOSID"、 "CYTOSINARABINOSIDE"、 "CYTOSINE ARABINOSE"、 "CYTOSINE-ARABINOSIDE"、 "Citarabin"、 "Citarabine"、 "Cytarabin accord"、 "Cytarabine (Ara-C)"、 "Cytarabine 167 mg"、 "Cytarabine Ebewe"、 "Cytarabine Intravenous Infusion 1g TEVA"、 "Cytarabine Intravenous Infusion 1g ^TEVA^"、 "DepoCyte (Liposomal cytarabine)"、 "ESHAP (CYTARABINE)"、 "INTRAVENTRICULAR LIPOSOMAL CYTA"、 "UDICIL"、 "ara?C"、 "ara?c"、 "aracytine"、 "citarabina"、 "cytarabine Pfizer"、 "cytarabine Sandoz"、 "cytarabine kabi"、 "liposomal cytarabine"、 |
| Bortezomib | "VELCADE"、 "BORTEZOMIB"、 "BORTEZOMIB."、 "Bortezomib"、 "bortezomib"、 "BORTEZOMIBE"、 "Bortezomib (Manufacturer U"、 "Bortezomib (Unknown)"、 "COMPARATOR BORTEZOMIB"、 "EXTERNAL-BORTEZOMIB"、 "BORTEZOMIB 3.5MG PWD SDV I"、 "Bortezomibe"、 "BORTEZOMIB FOR INJECTION"、 "BTZLBH589"、 "Bortezomib (Manufacturer Unknown)"、 "Bortezomib Hospira"、 "BORTEZOMIB GLENMARK, 1 MG,"、 "BORTEZOMIB HOSPIRA"、 "ACT BORTEZOMIB"、 "BORLEZOMIB (BORTEZOMIB)"、 "BORTEZOMIB (MANUFACTURER UNKNOWN) BORTEZOMIB) ("、 "BORTEZOMIB 3.5MG PWD SDV INJ"、 "BORTEZOMIB 3.5MG PWD SSV I"、 "BORTEZOMIB LAST DOSE 9/28/16 4."、 "BORTEZOMIB LAST DOSE 9/5/13 2.7 MG MILLENIUM PH"、 "BORTEZOMIB, 1.3 MG/M2"、 |
| Adriamycin | "ADRIAMYCIN"、 "DOXORUBICIN HYDROCHLORIDE."、 "DOXIL"、 "DOXORUBICIN HCL"、 "CAELYX"、 "ADRIACIN"、 "LIPOSOMAL DOXORUBICIN"、 "Doxorubicin Hydrochloride"、 "Adriblastina"、 "DOXORUBICIN HYDROCHLORIDE INJECTION"、 "Caelyx"、 "LIPOSOMAL DOXORUBICIN HCL"、 "PLD"、 "LIPOSOMAL DOXORUBICIN HYDROCHLORIDE"、 "PEGYLATED LIPOSOMAL DOXORUBICIN HYDROCHLORIDE"、 "Adriblastin"、 "DOXORUBICIN HYDROCHLORIDE"、 "ADRIBLASTIN"、 "DOXORUBICINA"、 "DOXORUBICINA (202A)"、 "Doxorubicin Hydrochloride Injec"、 "DOXORUBICIN HYDROCHLORIDE INJEC"、 "DOXORUBICINE HYDROCHLORIDE"、 "Doxorubicin Hydrochloride (Acta"、 "Doxorubicin Hydrochloride Liposome Injection 20 mg/10 mL (2 mg/mL) and"、 "ADRIBLASTINA"、 "LIPOSOMAL DOXORUBICIN HYDR"、 "DOXORUBICIN HYDROCHLORIDE (123127)"、 "CAYELIX"、 "Fauldoxo"、 "FAULDOXO"、 "Doxorubicin hcl"、 "MYOCET"、 "PEGYLATED LIPOSOMAL DOXORUBICIN"、 "DOXORUBICIN HCL PEG-LIPOSOMAL 2 MG/ML"、 "Doxorubicin Accord"、 "Doxorubicin HCl"、 "LIPODOX"、 "Doxorubicin HCL"、 "EXTERNAL-DOXORUBICIN HYDROCHLOR"、 "LIPOSOMAL DOXORUBICIN HYDROCHLO"、 "Liposomal doxorubicin"、 "liposomal doxorubicin"、 "CAELYX (DOXORUBICIN HYDROCHLORIDE)"、 "DOXORUBICIN ACCORD"、 "Doxorubicin Hydrochloride Liposome Injection 20"、 "PEGYLATED LIPOSOMAL DOXORU"、 "doxorubicin HCL"、 "Doxorubicin Hydrochloride Injection, USP"、 "Liposomal Doxorubicin"、 "PEGYLATED DOXORUBICIN HYDROCHLORIDE"、 "ADRIBLASTINA 50 MG/25 ML S"、 "CAELIX"、 "DOXORUBICIN HYDROCHLORIDE LIPOSOME"、 "DOXORUBICINA ACCORD HEALTH"、 "Doxorubicin hydrocholoride"、 "Farmiblastina"、 "doxorubicine hydrochloride"、 "liposomal Doxorubicin"、 "DOXORUBICIN HYDROCHLORIDE SANDOZ"、 "DOXORUBICIN HYDROCLORIDE"、 "DOXORUBICIN/DOXORUBICIN HYDROCH"、 "Doxorubicina"、 "PEGYLATED LIPOSOMAL DOXORUBICIN HCL"、 "caelyx"、 "doxorubicin HCl"、 "ADRIBLASTINA 50 MG/25 ML SOLUZIONE INIETTABILE PER USO ENDOVENOSO"、 "CAELYX (DOXORUBICIN HYDROCHLORI"、 "CALEYX"、 "CHLORHYDRATE DE DOXORUBICI"、 "DOXORUBICIN/DOXORUBICIN HY"、 "DOXORUBICINA PFIZER"、 "DOXORUBICINE"、 "MYOCET /00330902/"、 "NUDOXA"、 "liposomal doxorubicin HCL"、 "liposomal doxorubicin hcl"、 "Adriblastin RD"、 "Adriblastina RD"、 "CAELYX - JANSSEN-CILAG INTERNATIONAL N.V."、 "DOXORUBICIN HYDROCHLORID"、 "DOXORUBICIN HYDROCHLORIDE FOR INJECTION"、 "DOXORUBICIN HYDROCHLORIDE LIPOSOME INJECTION US"、 "DOXORUBICIN/DOXORUBICIN HYDROCHLORIDE"、 "DOXORUBICINE HYDROCHLORID"、 "DOXORUBIN HYDROCHLORIDE"、 "DOXOUBICIN HYDROCHLORIDE"、 "DUOMEISU"、 "LYPOSOMIAL DOXORUBICIN"、 "Liposomal doxorubicin HCL"、 "MYOCET - TEVA B.V. (LIPOSOMAL DOXORUBICIN HYDRO"、 "NON-PEGYLATED LIPOSOMAL DOXORUBICIN HYDROCHL."、 "doxorubicin Accord"、 "hydroxydoxorubicin"、 "ADRIABLASTIN"、 "ADRIABLASTINE"、 "ADRIBLASTIN RD"、 "ADRIBLASTINA - 10 MG/5 ML POLVE"、 "ADRIBLASTINA - 200MG/100ML SOLU"、 "ADRIBLASTINA - 50 MG/25 ML SOLU"、 "ADRIBLASTINA - PFIZER ITALIA S."、 "ADRIBLASTINA 200 MG/100 ML"、 "ADRIBLASTINA 200 MG/100 ML SOLUZIONE INIETTABILE PER USO ENDOVENOSO"、 "ADRICIN (DOXORUBICIN HYDROCHLORIDE)"、 "ADRIMEDAC"、 "Adriablastin"、 "CAELYX /00330904/"、 "CAELYX 2 MG/ML"、 "CAELYX 2 MG/ML, SOLUTION ?"、 "CAELYX 2 mg/ml, solution ?"、 "CAELYX AU"、 "CAELYX INJECTION"、 "CAEYLX"、 "DOKSORUBICIN ACCORD"、 "DOKSORUBICIN TEVA"、 "DOKSORUBICIN TEVA 2 MG/ML"、 "DOXORRUBICINA"、 "DOXORUBICIAN HYDROCHLORIDE"、 "DOXORUBICIN (HYDROXYDAUNOR"、 "DOXORUBICIN ACCORD 2 MG/ML"、 "DOXORUBICIN CHLORHYDRATE"、 "DOXORUBICIN HCI LIPOSOME"、 "DOXORUBICIN HCL LIPOSOME"、 "DOXORUBICIN HCL LIPOSOME, 40MG/"、 "DOXORUBICIN HYDRICHLORIDE"、 "DOXORUBICIN HYDRLCHLORIDE"、 "DOXORUBICIN HYDROCHLORIDE (CAEL"、 "DOXORUBICIN HYDROCHLORIDE 50MG [NK]"、 "DOXORUBICIN HYDROCHLORIDE LIPOSOMAL"、 "DOXORUBICIN HYDROCHLORIDE?"、 "DOXORUBICIN HYDRPCHLORIDE INJECTION"、 "DOXORUBICIN HYROCHLORIDE"、 "DOXORUBICIN LIPO 2MG/ML"、 "DOXORUBICIN ^TEVA^"、 "DOXORUBICINA ACCORD"、 "DOXORUBICINA ACCORD HEALTHCARE"、 "DOXORUBICINA ACCORD HEALTHCARE ITALIA"、 "DOXORUBICINA ACCORD HEALTHCARE ITALIA - 2MG/ML"、 "DOXORUBICINA SANDOZ"、 "DOXORUBICINE (CHLORHYDRATE"、 "DOXORUBICINE ACCORD 2 MG/M"、 "DOXORUBICINE TEVA 200 MG/1"、 "DOXORUBICINE TEVA 200 mg/1"、 "DOXORUBICINHYDROCHLORID-TEVA"、 "Doxorubician Hydrochloride"、 "Doxorubicin Hcl"、 "Doxorubicin Hydrochloride (Actavis Inc)"、 "Doxorubicin hydrochoride"、 "EXTERNAL-DOXORUBICIN HYDROCHLORIDE"、 "FARMIBLASTINA"、 "HYDROXYDAUNOMYCIN HYDROCHLORIDE"、 "HYDROXYDOXORUBICIN"、 "LIPOSOMAL DOXORUBICINE"、 "Liposomal doxorubicin hydr"、 "Liposomal doxorubicin hydrochlo"、 "MYOCET /00330903/"、 "MYOCET ? TEVA B.V."、 "RUBICIN"、 "RUBICIN /00330902/"、 "RUBIDOX (DOXORUBICIN HYDRO"、 "TETRAHYDROPYRANYL DOXORUBICIN"、 "YOCET 50 MG POWDER, DISPER"、 "adriblastin"、 "doxorubicin hcl"、 "pegylated liposomal doxoru"、 "pegylated liposomal doxorubicin hydrochloride"、 |
| Vinblastine | "VINBLASTINE"、 "Vinblastine"、 "VINBLASTIN"、 "vinblastine"、 "VINBLASTINE (manufacturer unkno"、 "Vinblastin"、 "VINBLASTINE (manufacturer unknown)"、 "VINBLASTINE (manufacturer"、 "vinblastin"、 "VINBLASTINE ^ROCHE^"、 "VINBLASTINE(VINBLASTINE)(V"、 "vinblastina"、 |
| Etoposide | "ETOPOSIDE."、 "ETOPOSIDE (VP-16)"、 "VP-16"、 "VEPESID"、 "ETOPOSIDE (Manufacturer Unknown)"、 "Etoposide (VP-16)"、 "ETOPOSIDE (Manufacturer Unknown"、 "ETOPOSIDE (Manufacturer Un"、 "ETOPOSIDE TEVA"、 "ETOPOSIDE MYLAN"、 "Etoposide (Unknown)"、 "LASTET"、 "VEPESIDE"、 "CELLTOP"、 "ETOPOSID"、 "Etoposide (VP?16)"、 "Etoposide Mylan"、 "ETOPOSIDE INJECTION, USP"、 "CELLTOP 100 MG/5 ML, SOLUT"、 "ETOPOSIDE CAP 50MG"、 "VP?16"、 "CELLTOP 100 MG/5 ML, SOLUTION I"、 "CELLTOP 100 MG/5 ML, SOLUTION INJECTABLE POUR P"、 "ETOPOSIDO (518A)"、 "Etoposide (Watson Laboratories)"、 "ETOPOSIDE INTRAVENOUS INFUSION"、 "Etoposid"、 "ETOPOSIDE (VP?16)"、 "Etoposide Teva"、 "CELLTOP 50 MG, CAPSULE"、 "ETOPOSIDE TEVA - TEVA PHARMA B.V."、 "ETOPOSIDO"、 "TOPOSAR"、 "Vepeside"、 "CELLTOP 25 MG, CAPSULE"、 "Celltop"、 "ETOPOSIDE INJECTION"、 "ETOPOSIDE INTRAVENOUS INFU"、 "Etoposide Capsules, USP"、 "LASTET S"、 "ETOPOSIDE ACCORD"、 "Etoposide Mylan 20 mg/ml, solution ? diluer pou"、 "VP16"、 "etoposide (vp-16)"、 "Etoposide Mylan 20 mg/ml,"、 "Tevaetopo"、 "ETOPOSIDE 50MG MYLAN"、 "ETOPOSIDE-TEVA"、 "VP 16-213"、 "CELLTOP 100 MG/5 ML, SOLUTION INJECTABLE POUR PERFUSION"、 "ETOPOSID EBEWE"、 "ETOPOSIDE"、 "ETOPOSIDE (TEVA)"、 "ETOPOSIDE 100MG/5ML ACCORD, INT"、 "ETOPOSIDE TEVA 20 mg/ml"、 "Etoposide-Teva"、 "VEPSID"、 "Vp-16"、 "ETOPOSIDE /00511902/"、 "ETOPOSIDE SANDOZ"、 "ETOPOSIDE- TEVA"、 "Etoposid Ebewe"、 "ETOPOSIDE 50 MG"、 "ETOPOSIDE INJECTION,USP"、 "ETOPOSIDE-TEVA 20 MG/ML CONCENT"、 "TOPOSIN, CONCENTRAAT VOOR OPLOS"、 "VP 16"、 "etoposid"、 "etoposide Teva"、 "ETOPOSIDE CAPSULES, USP"、 "ETOPOSIDE MERCK"、 "ETOPOSIDE SANDOZ - 20 MG/ML CONCENTRATO PER SOL"、 "ETOPOSIDE SANDOZ ? 20 MG/M"、 "ETOPOSIDE TEVA - 20 MG/ML CONCENTRATO PER SOLUZ"、 "ETOPOSIDO TEVA 20 MG/ML CONCENT"、 "Etoposide Mylan 20 mg/ml, solut"、 "Etoposide mylan"、 "Teva-Etoposide"、 "etoposide (VP-16)"、 "ACCORD ETOPOSIDE"、 "Celltop - capsules molles"、 "ETOPOSIDE (G)"、 "ETOPOSIDE (MYLAN)"、 "ETOPOSIDE 50 MG CAPSULE"、 "ETOPOSIDE INJECTION USP"、 "ETOPOSIDE LIQ IV 20MG/ML"、 "ETOPOSIDE MYLAN 20 MG/ML,"、 "ETOPOSIDE MYLAN 20 mg/ml"、 "ETOPOSIDE MYLAN 20 mg/ml,"、 "ETOPOSIDE MYLAN 20 mg/ml, solution ? diluer pour perfusion"、 "ETOPOSIDE TEVA - TEVA PHARMA B."、 "ETOPOSIDE TEVA 20 MG/ML"、 "ETOPOSIDE TEVA 20 mg/mL"、 "ETOPOSIDE-Teva"、 "ETOPOSIDO TEVAGEN 20 MG/ML CONCENTRATE FOR SOLU"、 "ETOPSIDE MYLAN"、 "EXITOP"、 "Eposin 20 MG/ML"、 "Etoposide Accord"、 "Etoposide Mylan 20 mg/ml, solution ? diluer pour perfusion"、 "Etoposide Sandoz"、 "Lastet"、 "TEVA-ETOPOSIDE"、 "VEPEZIDE"、 "Vepezide"、 "vepeside"、 "BLINDED ETOPOSIDE"、 "CELLTOP 50 MG - CAPSULES MOLLES"、 "CELLTOP 50 mg, capsule"、 "EPOSIDO"、 "EPOSIN 20MG/ML"、 "ESHAP (ETOPOSIDE)"、 "ETHOPOSI [ETOPOSIDE]"、 "ETHOPOSIDE"、 "ETO-GRY (ETOPOSIDE)"、 "ETOMEDAC"、 "ETOPOSIDE (VP -16)"、 "ETOPOSIDE (VP 16)"、 "ETOPOSIDE (VP-16) 2700 MG"、 "ETOPOSIDE (VP-6)"、 "ETOPOSIDE (VP16)"、 "ETOPOSIDE / 50 MG"、 "ETOPOSIDE / 50 MG MYLAN"、 "ETOPOSIDE 20 MG/ML"、 "ETOPOSIDE 20 MG/ML MDV"、 "ETOPOSIDE 223 mg"、 "ETOPOSIDE 50MG"、 "ETOPOSIDE 50MG CAPSULES"、 "ETOPOSIDE BASE"、 "ETOPOSIDE BMS"、 "ETOPOSIDE CAP"、 "ETOPOSIDE CAP 50 MG"、 "ETOPOSIDE CAPSULES 50 MG"、 "ETOPOSIDE EBEWE"、 "ETOPOSIDE ETOPOSIDE"、 "ETOPOSIDE HENGRUI PHARMACE"、 "ETOPOSIDE INJ 20MG/ML"、 "ETOPOSIDE INTRAVENOUS INFUSION 100MG TAIYO"、 "ETOPOSIDE MYLAN 20 mg/ml, solution ? diluer pou"、 "ETOPOSIDE TEVA - 10 ML 20MG/ML"、 "ETOPOSIDE TEVA - 20 MG/ML CONCE"、 "ETOPOSIDE TEVA - 20 MG/ML CONCENTRATO PER SOLU"、 "ETOPOSIDE TEVA - 50 ML 20 MG/ML"、 "ETOPOSIDE TEVA - FLACONE 5 ML 2"、 "ETOPOSIDE TEVA 20 MG/ML, C"、 "ETOPOSIDE TEVA 20 MG/ML, S"、 "ETOPOSIDE TEVA 20 mg/ml, s"、 "ETOPOSIDE TEVA 200 MG"、 "ETOPOSIDE TEVA 200 MG/10 M"、 "ETOPOSIDE TEVA 200 mg/10 m"、 "ETOPOSIDE TEVA 200 mg/10 ml, solution injectable pour perfusion"、 "ETOPOSIDE TEVA 20mg/ml"、 "ETOPOSIDE TEVA 5"、 "ETOPOSIDE TEVA ? 20 MG/ML"、 "ETOPOSIDE Teva"、 "ETOPOSIDE, 20 MG/ML"、 "ETOPOSIDE, 50 MG"、 "ETOPOSIDE, CONCENTRATE FOR SOLU"、 "ETOPOSIDE?TEVA"、 "ETOPOSIDO ACCORD"、 "ETOPOSIDO TEVA 100 MG/5 ML SOLU"、 "ETOPOSIDO TEVA 20 MG/ML"、 "ETOPOSIDO TEVA 20 MG/ML CO"、 "ETOPOSIDO TEVAGEN 20MG/ML CONCENTRADO PARA SOLU"、 "ETOPSOIDE 50MG"、 "ETP (ETOPOSIDE)"、 "EXITOP KONSENTRAT TIL INFUSJONS"、 "Eposin"、 "Etoposide 50 mg"、 "Etoposide Intravenous Infusion 100mg ^TAIYO^"、 "Etoposide VP-16"、 "Etoposide accord"、 "Etoposide."、 "FYTOSID"、 "LASTET INJ. 100MG/5ML"、 "Teva?ETOPOSIDE"、 "VEPESID J"、 "VP 16?213"、 "Vepsid"、 "[PSS GPN] ETOPOSIDE"、 "etoposide Mylan"、 "etoposide VP-16"、 "etoposido"、 |
| Teniposide | "TENIPOSIDE."、 "VUMON"、 "VM 26"、 "TENIPOSIDE (VM-26)"、 |

# Table S4 ROR025 of the association between class-specific ICIs monotherapy/polytherapy (without chemotherapy) and renal AEs in FAERS database

| PT | Nivo | Pemb | Cemi | Atez | Avel | Durv | Ipil | Poly1 | Poly2 | Poly3 |
| --- | --- | --- | --- | --- | --- | --- | --- | --- | --- | --- |
| Abdominal abscess |  |  |  |  |  |  |  |  | 1.54 |  |
| Abdominal discomfort | 0.23 | 0.39 |  |  |  | 0.63 |  |  | 0.18 |  |
| Abdominal distension | 0.75 | 0.50 |  |  |  |  | 0.44 |  | 0.53 |  |
| Abdominal infection |  |  |  |  |  |  |  |  | 3.28 |  |
| Abdominal lymphadenopathy | 1.01 |  |  |  |  |  |  |  |  |  |
| Abdominal pain | 1.54 | 1.40 |  | 1.69 |  | 0.50 | 2.05 |  | 1.92 |  |
| Abdominal pain lower | 0.27 |  |  |  |  |  |  |  |  |  |
| Abdominal pain upper | 0.87 | 0.54 |  | 0.49 |  | 0.99 | 0.70 |  | 0.83 |  |
| Abdominal symptom | 1.62 |  |  |  |  |  |  |  |  |  |
| Acute kidney injury | 1.66 | 1.28 | 0.97 | 3.47 | 0.66 | 2.19 | 1.04 |  | 3.19 | 1.70 |
| Ageusia | 0.78 |  |  |  |  |  |  |  |  |  |
| Anal incontinence |  |  |  |  |  |  |  |  | 0.34 |  |
| Anal ulcer | 0.99 |  |  |  |  |  |  |  |  |  |
| Anuria | 0.74 |  |  |  |  |  |  |  |  |  |
| Aphthous ulcer | 0.70 |  |  |  |  |  |  |  |  |  |
| Appendicitis |  | 1.00 |  |  |  |  |  |  | 1.98 |  |
| Ascites | 5.33 | 3.05 |  | 4.01 |  |  |  |  | 2.41 |  |
| Autoimmune colitis | 21.43 | 29.31 |  | 13.57 |  |  | 47.86 | 3.30 | 107.49 | 22.38 |
| Autoimmune nephritis | 7.79 | 6.83 |  |  |  |  |  |  | 14.33 |  |
| Autoimmune pancreatitis | 9.81 | 6.04 |  |  |  |  |  | 8.30 | 18.81 |  |
| Azotaemia | 0.75 |  |  |  |  |  |  |  |  |  |
| Bladder cancer | 0.50 | 0.39 |  | 0.55 |  |  |  |  |  |  |
| Cheilitis |  | 1.90 |  |  |  |  |  |  |  |  |
| Chromaturia | 0.67 | 0.65 |  |  |  |  | 1.24 |  | 2.44 |  |
| Chronic gastritis |  | 1.80 |  |  |  |  |  |  |  |  |
| Chronic kidney disease | 0.27 | 0.15 |  | 0.40 |  | 0.33 | 0.26 |  | 0.10 |  |
| Clostridium difficile colitis | 0.31 | 1.55 |  | 2.16 |  |  | 0.65 |  | 1.62 |  |
| Coating in mouth | 2.72 |  |  |  |  |  |  |  |  |  |
| Colitis | 11.23 | 10.92 |  | 8.80 | 3.59 | 4.58 | 61.34 |  | 44.53 | 13.75 |
| Colitis ischaemic | 0.75 |  |  |  |  |  |  |  |  |  |
| Colitis microscopic | 1.71 | 1.95 |  |  |  |  | 2.32 |  |  |  |
| Colitis ulcerative | 0.77 | 1.97 |  |  |  |  | 1.33 |  | 0.28 |  |
| Colon cancer | 0.90 |  |  |  |  |  |  |  |  |  |
| Constipation | 1.23 | 0.99 |  | 0.67 |  |  | 0.84 |  | 0.94 |  |
| Crohn disease | 0.07 |  |  |  |  |  |  |  | 0.32 |  |
| Cystic fibrosis | 0.28 |  |  |  |  |  |  |  |  |  |
| Cystitis | 0.70 |  |  |  |  |  | 0.18 |  | 1.32 |  |
| Cystitis haemorrhagic | 1.64 |  |  |  |  |  |  |  |  |  |
| Cystitis noninfective | 2.21 |  |  |  |  |  |  |  | 1.36 |  |
| Cytomegalovirus colitis | 0.54 |  |  |  |  |  |  |  |  |  |
| Cytomegalovirus enterocolitis | 4.30 |  |  |  |  |  |  |  | 3.00 |  |
| Diarrhoea | 1.47 | 1.20 | 0.50 | 0.97 |  | 1.06 | 3.68 |  | 2.62 | 1.15 |
| Diarrhoea haemorrhagic |  |  |  |  |  |  |  |  | 0.65 |  |
| Diverticulitis | 0.38 |  |  | 1.09 |  |  |  |  | 0.43 |  |
| Dry mouth | 0.67 | 0.86 |  | 0.43 |  |  |  |  | 0.48 |  |
| Duodenal perforation | 2.55 | 3.44 |  |  | 4.37 |  |  |  |  |  |
| Duodenal ulcer | 1.15 | 2.30 |  |  |  |  |  |  |  |  |
| Duodenitis |  | 0.96 |  |  |  |  |  |  | 5.78 | 7.98 |
| Dysgeusia | 0.67 | 0.45 |  |  |  |  |  |  |  |  |
| Dyspepsia | 0.20 |  |  |  |  |  |  |  | 0.24 |  |
| Dysphagia | 1.28 | 0.89 |  | 0.63 |  | 0.69 |  |  | 0.80 |  |
| Dysuria | 0.38 | 0.33 |  |  |  |  |  |  | 0.42 |  |
| End stage renal disease | 0.29 | 0.14 |  | 0.29 |  |  |  |  | 0.11 |  |
| Enteritis | 3.01 | 3.63 |  |  |  |  | 3.20 |  | 17.72 |  |
| Enteritis infectious | 2.51 |  |  |  |  |  |  |  |  |  |
| Enterocolitis | 10.99 | 3.23 |  | 2.43 |  |  | 21.91 |  | 30.31 |  |
| Enterocolitis haemorrhagic | 2.41 |  |  |  |  |  |  |  |  |  |
| Escherichia urinary tract infection | 0.54 |  |  |  |  |  |  |  |  |  |
| Faeces discoloured | 0.46 |  |  |  |  |  |  |  |  |  |
| Fistula of small intestine |  |  |  | 3.00 |  |  |  |  |  |  |
| Flank pain | 0.92 | 1.19 |  |  |  |  |  |  |  |  |
| Flatulence | 0.13 | 0.18 |  |  |  |  |  |  |  |  |
| Fluid retention | 0.21 | 0.16 |  | 0.19 |  |  |  |  |  |  |
| Focal segmental glomerulosclerosis | 0.89 |  |  |  |  |  |  |  |  |  |
| Food poisoning | 0.37 |  |  |  |  |  |  |  |  |  |
| Frequent bowel movements | 0.64 |  |  |  |  |  |  |  |  |  |
| Fungal oesophagitis |  |  |  |  |  |  |  |  | 2.11 |  |
| Gastric cancer |  | 0.70 |  |  |  |  |  |  |  |  |
| Gastric disorder |  | 0.12 |  |  |  |  |  |  |  |  |
| Gastric haemorrhage |  |  |  | 1.38 |  |  |  |  |  |  |
| Gastric mucosal lesion |  | 2.83 |  |  |  |  |  |  |  |  |
| Gastric perforation | 0.96 | 0.94 |  |  |  |  |  |  |  |  |
| Gastric ulcer | 0.70 | 0.43 |  |  |  |  |  |  | 0.29 |  |
| Gastric ulcer haemorrhage | 0.68 | 0.25 |  |  | 4.74 |  |  |  |  |  |
| Gastritis | 0.87 | 1.02 |  |  |  |  | 1.60 |  | 6.83 | 5.45 |
| Gastritis erosive |  | 0.58 |  |  |  |  |  |  |  |  |
| Gastritis haemorrhagic | 2.06 |  |  |  |  |  |  |  |  |  |
| Gastroenteritis | 0.95 | 0.65 |  |  |  |  |  |  | 2.87 |  |
| Gastrointestinal disorder | 0.63 | 0.22 |  |  |  |  | 1.52 |  | 0.71 |  |
| Gastrointestinal haemorrhage | 0.37 | 0.29 |  | 0.36 |  |  |  |  | 0.18 |  |
| Gastrointestinal inflammation |  |  |  |  |  |  |  |  | 0.97 |  |
| Gastrointestinal necrosis | 0.76 |  |  |  |  |  |  |  |  |  |
| Gastrointestinal pain |  | 0.23 |  |  |  |  |  |  |  |  |
| Gastrointestinal perforation | 4.85 | 2.83 |  |  |  |  |  |  | 4.11 |  |
| Gastrointestinal toxicity | 2.13 |  |  |  |  |  |  |  | 5.69 |  |
| Gastrointestinal ulcer | 0.44 |  |  |  |  |  |  |  |  |  |
| Gingival bleeding | 0.38 |  |  |  |  |  |  |  |  |  |
| Gingival swelling | 0.30 |  |  |  |  |  |  |  |  |  |
| Glomerulonephritis | 2.12 | 1.17 |  |  |  |  |  |  |  |  |
| Glossitis |  | 1.97 |  |  |  |  |  |  |  |  |
| Granulomatosis with polyangiitis |  |  |  |  |  |  |  |  | 1.54 |  |
| Haematemesis | 0.70 | 0.46 |  |  |  |  |  |  | 0.17 |  |
| Haematochezia | 0.66 | 0.72 |  |  |  |  |  |  |  |  |
| Haematuria | 0.81 | 0.70 |  | 3.09 |  |  | 0.43 |  | 0.56 |  |
| Haemolytic uraemic syndrome | 0.34 |  |  |  |  |  |  |  | 0.69 |  |
| Hydronephrosis | 2.31 | 2.00 |  |  |  |  |  |  |  |  |
| Hyperparathyroidism secondary |  |  |  |  |  |  |  |  | 0.89 |  |
| Hypophagia | 0.46 | 0.74 |  |  |  |  |  |  | 0.43 |  |
| Iga nephropathy |  | 1.03 |  |  |  |  |  |  |  |  |
| Ileal perforation |  |  |  | 8.72 |  |  |  |  |  |  |
| Ileus | 3.07 | 1.97 |  | 2.52 |  |  | 1.58 |  | 2.34 |  |
| Ileus paralytic | 1.81 |  |  |  |  |  |  |  | 0.97 | 7.61 |
| Incontinence | 0.63 |  |  |  |  |  |  |  | 0.59 |  |
| Inflammatory bowel disease | 1.12 |  |  |  |  |  |  |  |  |  |
| Inguinal hernia | 0.79 |  |  |  |  |  |  |  |  |  |
| Intestinal obstruction | 0.72 | 0.67 |  |  |  | 0.83 | 0.47 |  | 0.75 |  |
| Intestinal perforation | 1.18 | 1.52 |  | 1.64 |  |  | 7.63 |  | 3.87 |  |
| Intestinal pseudo-obstruction |  |  |  |  |  |  |  |  | 2.18 |  |
| Intra-abdominal haemorrhage |  | 0.59 |  |  |  |  |  |  |  |  |
| Intussusception |  | 2.33 |  |  |  |  |  |  |  |  |
| Jejunal perforation | 1.83 | 2.36 |  |  |  |  |  |  |  |  |
| Kidney fibrosis | 0.74 |  |  |  |  |  | 2.42 |  | 1.85 |  |
| Kidney infection | 0.19 | 0.17 |  |  |  |  |  |  |  |  |
| Kidney transplant rejection | 2.84 | 1.78 |  |  |  |  |  |  | 1.57 | 4.12 |
| Large intestinal obstruction | 0.62 |  |  |  |  |  |  |  | 1.65 |  |
| Large intestine perforation | 1.21 | 1.02 |  |  |  |  |  |  | 1.09 |  |
| Lip oedema | 0.70 |  |  |  |  |  |  |  |  |  |
| Lip swelling |  | 0.36 |  |  |  |  |  |  |  |  |
| Malignant ascites |  | 1.87 |  |  |  |  |  |  |  |  |
| Malignant peritoneal neoplasm | 4.22 |  |  |  |  |  |  |  |  |  |
| Melaena | 1.32 | 0.63 |  |  |  |  |  |  | 0.25 |  |
| Metastases to kidney | 4.45 |  |  |  |  |  |  |  |  |  |
| Metastases to pancreas | 2.13 |  |  |  |  |  |  |  |  |  |
| Metastases to peritoneum | 1.12 |  |  |  |  |  |  |  |  |  |
| Metastatic renal cell carcinoma | 0.48 |  |  |  |  |  |  |  |  |  |
| Micturition urgency | 0.17 | 0.21 |  |  |  |  |  |  |  |  |
| Mouth haemorrhage | 0.70 |  |  |  |  |  |  |  |  |  |
| Mouth swelling |  | 0.63 |  |  |  |  |  |  |  |  |
| Mouth ulceration | 0.90 | 0.34 |  |  |  |  |  |  |  |  |
| Nausea | 0.46 | 0.59 |  | 0.47 |  | 0.37 | 0.64 |  | 0.73 |  |
| Nephritis | 8.94 | 15.15 |  | 12.68 |  |  | 1.47 |  | 11.20 |  |
| Nephrogenic anaemia | 0.34 |  |  |  |  |  |  |  |  |  |
| Nephrolithiasis | 0.08 | 0.10 |  |  |  |  |  |  |  |  |
| Nephropathy | 0.25 | 0.48 |  |  |  |  |  |  |  |  |
| Nephropathy toxic | 0.72 | 0.32 |  |  |  |  |  |  |  |  |
| Nephrotic syndrome | 2.86 | 1.36 |  |  |  |  |  |  | 0.57 |  |
| Neurogenic bladder | 0.73 |  |  |  |  |  |  |  |  |  |
| Oesophageal candidiasis | 0.66 |  |  |  |  |  |  |  |  |  |
| Oesophageal carcinoma |  | 0.85 |  |  |  |  |  |  |  |  |
| Oesophageal fistula | 6.23 |  |  |  |  |  |  |  |  |  |
| Oesophageal perforation |  | 2.48 |  |  |  |  |  |  |  |  |
| Oesophagitis | 1.57 | 0.84 |  |  |  |  |  |  | 1.51 |  |
| Oral candidiasis | 1.25 |  |  |  |  |  |  |  | 1.12 |  |
| Oral herpes | 0.54 |  |  |  |  |  |  |  |  |  |
| Oral lichen planus | 0.86 | 1.84 |  |  |  |  |  |  |  |  |
| Oral mucosa erosion | 0.81 |  |  |  |  |  |  |  |  |  |
| Oral mucosal blistering | 0.74 | 0.56 |  |  |  |  |  |  |  |  |
| Oral pain | 0.29 |  |  |  |  |  |  |  |  |  |
| Oropharyngeal pain | 0.22 |  |  |  |  |  |  |  |  |  |
| Pancreatic atrophy | 5.92 | 2.50 |  |  |  |  | 2.96 |  | 1.77 |  |
| Pancreatic disorder | 1.04 |  |  |  |  |  |  |  |  |  |
| Pancreatic failure |  |  |  |  |  |  |  |  | 2.65 |  |
| Pancreatitis | 1.26 | 1.08 |  | 2.24 | 2.16 | 2.75 |  |  | 1.97 |  |
| Pancreatitis acute | 0.56 | 0.28 |  |  |  |  |  |  |  |  |
| Parotitis |  | 0.97 |  |  |  |  |  |  |  |  |
| Pelvic pain | 0.08 |  |  |  |  |  |  |  |  |  |
| Peritonitis | 0.66 |  |  | 0.55 |  |  |  |  |  |  |
| Pharyngitis | 0.26 |  |  |  |  |  |  |  |  |  |
| Pneumatosis intestinalis | 2.10 |  |  |  |  |  |  |  |  |  |
| Pneumoperitoneum |  | 0.70 |  |  |  |  |  |  |  |  |
| Pollakiuria | 0.46 | 0.18 |  |  |  |  |  |  |  |  |
| Polyuria | 0.35 |  |  |  |  |  |  |  |  |  |
| Prerenal failure | 1.91 |  |  |  |  |  |  |  |  |  |
| Proctitis | 1.62 |  |  | 4.63 |  |  |  |  | 3.72 |  |
| Proteinuria | 0.76 | 0.42 |  |  |  |  |  |  |  |  |
| Pyelonephritis |  |  |  |  |  |  | 0.67 |  |  |  |
| Rectal haemorrhage | 0.24 |  |  |  |  | 0.33 |  |  | 0.29 |  |
| Renal cancer | 0.12 |  |  |  |  |  |  |  |  |  |
| Renal cyst | 0.21 |  |  |  |  |  |  |  |  |  |
| Renal disorder | 1.45 | 0.65 |  |  |  |  |  |  | 0.43 |  |
| Renal failure | 0.68 | 0.48 |  | 0.76 | 0.80 |  | 0.11 |  | 0.57 |  |
| Renal haemorrhage | 0.40 |  |  |  |  |  |  |  |  |  |
| Renal impairment | 1.06 | 1.18 |  | 0.69 |  |  | 0.49 |  | 0.33 |  |
| Renal infarct | 0.62 |  |  |  |  |  |  |  |  |  |
| Renal injury | 0.27 | 0.11 |  | 0.18 |  |  | 0.14 |  | 0.09 |  |
| Renal pain | 0.16 |  |  |  |  |  |  |  |  |  |
| Renal tubular disorder |  | 0.38 |  |  |  |  |  |  | 1.20 |  |
| Renal tubular necrosis | 0.22 | 0.51 |  |  |  |  |  |  |  |  |
| Retroperitoneal fibrosis |  | 1.53 |  |  |  |  |  |  |  |  |
| Sialoadenitis | 2.79 |  |  |  |  |  |  |  | 1.88 |  |
| Sjogren syndrome | 2.36 | 4.55 |  |  |  |  |  |  | 6.65 |  |
| Small intestinal obstruction | 0.55 | 0.62 |  | 2.78 |  |  |  |  | 1.23 |  |
| Small intestinal perforation | 1.29 | 3.70 |  |  |  |  |  |  |  |  |
| Stomatitis | 1.28 | 0.77 |  |  |  |  |  |  | 0.26 |  |
| Swollen tongue | 0.27 |  |  |  |  |  |  |  |  |  |
| Systemic scleroderma |  | 3.40 |  |  |  |  |  |  |  |  |
| Thrombotic microangiopathy |  | 0.16 |  |  |  |  |  |  |  |  |
| Tooth disorder | 0.16 |  |  |  |  |  |  |  | 0.09 |  |
| Tubulointerstitial nephritis | 0.80 | 1.31 |  | 0.92 |  |  | 0.59 |  | 1.38 |  |
| Urinary incontinence | 0.29 | 0.28 |  |  |  |  |  |  |  |  |
| Urinary retention | 0.65 | 0.51 |  | 0.90 |  |  | 0.18 |  | 0.19 |  |
| Urinary tract disorder | 0.21 |  |  |  |  |  |  |  |  |  |
| Urinary tract infection | 0.34 | 0.21 |  | 0.63 |  |  | 0.10 |  | 0.48 |  |
| Urinary tract obstruction | 0.35 | 0.84 |  |  |  |  |  |  | 1.59 |  |
| Urosepsis | 0.26 | 0.17 |  | 2.55 |  |  |  |  | 0.62 |  |
| Varices oesophageal | 0.55 |  |  |  |  |  |  |  |  |  |
| Vomiting | 0.45 | 0.46 |  | 0.34 |  | 0.84 | 0.55 |  | 0.67 |  |

*In Table S4, nivo, pemb, cemi, atez, avel, durv, ipil, poly1, poly2 and poly3 were respectively represent nivolumab, pembrolizumab, cemiplimab, atezolizumab, avelumab, durvalumab, ipilimumab, nivolumab+ ipilimumab+ pembrolizumab, nivolumab+ ipilimumab and ipilimumab+ pembrolizumab.

# Table S5 ROR025 of the association between class-specific ICIs monotherapy/polytherapy (with chemotherapy) and renal AEs in FAERS database

| **PT** | **Nivo** | **Pemb** | **Cemi** | **Atez** | **Avel** | **Durv** | **Ipil** | **Poly1** | **Poly2** | **Poly3** |
| --- | --- | --- | --- | --- | --- | --- | --- | --- | --- | --- |
| Abdominal pain | 1.95 |  |  | 1.61 |  |  |  |  | 1.91 |  |
| Abdominal pain upper | 0.61 |  |  |  |  |  |  |  |  |  |
| Acute kidney injury | 3.68 | 1.31 |  | 2.69 | 0.94 | 0.95 | 1.08 |  | 6.62 |  |
| Ascites | 1.94 |  |  | 2.22 |  |  |  |  |  |  |
| Autoimmune colitis |  |  |  |  |  |  |  |  | 9.31 |  |
| Bladder mass |  |  |  |  |  |  |  |  |  | 17.94 |
| Chronic kidney disease | 0.10 |  |  |  |  |  |  |  |  |  |
| Colitis | 5.09 | 6.15 |  | 9.17 |  |  |  |  | 9.32 | 2.05 |
| Constipation |  | 0.99 |  |  |  |  |  |  |  |  |
| Diarrhoea | 1.27 | 1.06 |  | 0.98 |  | 0.53 |  |  | 1.98 |  |
| Diverticulitis | 0.59 |  |  |  |  |  |  |  |  |  |
| Duodenal ulcer | 1.44 |  |  |  |  |  |  |  |  |  |
| Dysgeusia |  | 1.53 |  |  |  |  |  |  |  |  |
| Dysphagia | 0.82 |  |  |  |  |  |  |  |  |  |
| End stage renal disease |  | 0.55 |  |  |  |  |  |  |  |  |
| Enteritis |  |  |  | 3.10 |  |  |  |  | 5.50 |  |
| Enterocolitis | 7.12 |  |  |  |  |  |  |  | 12.08 |  |
| Gastroenteritis |  |  |  |  |  |  |  |  | 3.40 |  |
| Intestinal obstruction |  |  |  | 2.30 |  |  |  |  |  |  |
| Nausea | 0.58 | 0.88 |  |  | 1.04 |  |  |  | 0.81 |  |
| Nephritis |  |  |  | 2.71 |  |  |  |  |  |  |
| Oesophageal candidiasis |  |  |  | 1.66 |  |  |  |  |  |  |
| Oral candidiasis | 3.55 |  |  |  |  |  |  |  |  |  |
| Renal failure | 0.14 | 0.41 |  | 0.49 | 0.50 |  |  |  | 1.44 |  |
| Renal impairment | 0.20 | 0.91 |  | 0.78 |  |  |  |  |  |  |
| Renal injury | 0.27 |  |  |  |  |  |  |  |  |  |
| Small intestinal obstruction |  | 1.55 |  | 3.56 |  |  |  |  |  |  |
| Stomatitis |  | 1.29 |  |  |  |  |  |  | 0.77 |  |
| Tubulointerstitial nephritis |  |  |  | 2.38 |  |  |  |  |  |  |
| Urinary tract infection |  |  |  | 0.50 | 1.63 |  |  |  | 0.36 |  |
| Vomiting | 0.60 | 0.77 |  | 0.22 |  |  |  |  | 0.65 |  |

*In Table S5, nivo, pemb, cemi, atez, avel, durv, ipil, poly1, poly2 and poly3 were respectively represent nivolumab, pembrolizumab, cemiplimab, atezolizumab, avelumab, durvalumab, ipilimumab, nivolumab+ ipilimumab+ pembrolizumab, nivolumab+ ipilimumab and ipilimumab+ pembrolizumab.

# Table S6 IC025 of the association between class-specific ICIs monotherapy/polytherapy (without chemotherapy) and renal AEs in FAERS database

| **PT** | **Nivo** | **Pemb** | **Cemi** | **Atez** | **Avel** | **Durv** | **Ipil** | **Poly1** | **Poly2** | **Poly3** |
| --- | --- | --- | --- | --- | --- | --- | --- | --- | --- | --- |
| Abdominal abscess |  |  |  |  |  |  |  |  | 0.29 |  |
| Abdominal discomfort | -2.17 | -1.44 |  |  |  | -0.95 |  |  | -2.60 |  |
| Abdominal distension | -0.47 | -1.10 |  |  |  |  | -1.37 |  | -1.03 |  |
| Abdominal infection |  |  |  |  |  |  |  |  | 1.45 |  |
| Abdominal lymphadenopathy | -0.64 |  |  |  |  |  |  |  |  |  |
| Abdominal pain | 0.59 | 0.44 |  | 0.67 |  | -1.27 | 0.97 |  | 0.90 |  |
| Abdominal pain lower | -2.13 |  |  |  |  |  |  |  |  |  |
| Abdominal pain upper | -0.24 | -0.97 |  | -1.20 |  | -0.22 | -0.63 |  | -0.33 |  |
| Abdominal symptom | 0.21 |  |  |  |  |  |  |  |  |  |
| Acute kidney injury | 0.70 | 0.31 | -0.70 | 1.73 | -0.98 | 1.02 | -0.02 |  | 1.63 | 0.52 |
| Ageusia | -0.49 |  |  |  |  |  |  |  |  |  |
| Anal incontinence |  |  |  |  |  |  |  |  | -2.21 |  |
| Anal ulcer | -0.66 |  |  |  |  |  |  |  |  |  |
| Anuria | -0.66 |  |  |  |  |  |  |  |  |  |
| Aphthous ulcer | -0.77 |  |  |  |  |  |  |  |  |  |
| Appendicitis |  | -0.23 |  |  |  |  |  |  | 0.81 |  |
| Ascites | 2.36 | 1.53 |  | 1.86 |  |  |  |  | 1.17 |  |
| Autoimmune colitis | 4.29 | 4.76 |  | 3.60 |  |  | 5.49 | 1.25 | 6.59 | 4.36 |
| Autoimmune nephritis | 2.75 | 2.54 |  |  |  |  |  |  | 3.70 |  |
| Autoimmune pancreatitis | 3.13 | 2.36 |  |  |  |  |  | 2.82 | 4.10 |  |
| Azotaemia | -0.80 |  |  |  |  |  |  |  |  |  |
| Bladder cancer | -1.12 | -1.61 |  | -1.35 |  |  |  |  |  |  |
| Cheilitis |  | 0.67 |  |  |  |  |  |  |  |  |
| Chromaturia | -0.71 | -0.81 |  |  |  |  | 0.08 |  | 1.18 |  |
| Chronic gastritis |  | 0.51 |  |  |  |  |  |  |  |  |
| Chronic kidney disease | -1.92 | -2.80 |  | -1.45 |  | -1.87 | -2.08 |  | -3.45 |  |
| Clostridium difficile colitis | -1.95 | 0.47 |  | 0.82 |  |  | -1.10 |  | 0.52 |  |
| Coating in mouth | 1.11 |  |  |  |  |  |  |  |  |  |
| Colitis | 3.43 | 3.39 |  | 3.05 | 1.56 | 2.03 | 5.83 |  | 5.37 | 3.64 |
| Colitis ischaemic | -0.62 |  |  |  |  |  |  |  |  |  |
| Colitis microscopic | 0.62 | 0.76 |  |  |  |  | 0.93 |  |  |  |
| Colitis ulcerative | -0.49 | 0.89 |  |  |  |  | 0.23 |  | -2.19 |  |
| Colon cancer | -0.28 |  |  |  |  |  |  |  |  |  |
| Constipation | 0.26 | -0.07 |  | -0.72 |  |  | -0.33 |  | -0.14 |  |
| Crohn disease | -4.24 |  |  |  |  |  |  |  | -1.87 |  |
| Cystic fibrosis | -2.18 |  |  |  |  |  |  |  |  |  |
| Cystitis | -0.61 |  |  |  |  |  | -3.13 |  | 0.28 |  |
| Cystitis haemorrhagic | 0.52 |  |  |  |  |  |  |  |  |  |
| Cystitis noninfective | 0.81 |  |  |  |  |  |  |  | -0.21 |  |
| Cytomegalovirus colitis | -1.54 |  |  |  |  |  |  |  |  |  |
| Cytomegalovirus enterocolitis | 1.86 |  |  |  |  |  |  |  | 1.20 |  |
| Diarrhoea | 0.53 | 0.23 | -1.64 | -0.11 |  | -0.02 | 1.82 |  | 1.35 | 0.03 |
| Diarrhoea haemorrhagic |  |  |  |  |  |  |  |  | -1.00 |  |
| Diverticulitis | -1.51 |  |  | -0.13 |  |  |  |  | -1.44 |  |
| Dry mouth | -0.65 | -0.31 |  | -1.55 |  |  |  |  | -1.19 |  |
| Duodenal perforation | 1.11 | 1.52 |  |  | 1.74 |  |  |  |  |  |
| Duodenal ulcer | 0.02 | 1.03 |  |  |  |  |  |  |  |  |
| Duodenitis |  | -0.55 |  |  |  |  |  |  | 2.36 | 2.75 |
| Dysgeusia | -0.65 | -1.29 |  |  |  |  |  |  |  |  |
| Dyspepsia | -2.42 |  |  |  |  |  |  |  | -2.24 |  |
| Dysphagia | 0.31 | -0.25 |  | -0.89 |  | -0.92 |  |  | -0.43 |  |
| Dysuria | -1.53 | -1.81 |  |  |  |  |  |  | -1.48 |  |
| End stage renal disease | -1.90 | -3.16 |  | -2.28 |  |  |  |  | -3.56 |  |
| Enteritis | 1.48 | 1.73 |  |  |  |  | 1.46 |  | 4.06 |  |
| Enteritis infectious | 1.12 |  |  |  |  |  |  |  |  |  |
| Enterocolitis | 3.38 | 1.55 |  | 0.95 |  |  | 4.37 |  | 4.84 |  |
| Enterocolitis haemorrhagic | 1.05 |  |  |  |  |  |  |  |  |  |
| Escherichia urinary tract infection | -1.39 |  |  |  |  |  |  |  |  |  |
| Faeces discoloured | -1.27 |  |  |  |  |  |  |  |  |  |
| Fistula of small intestine |  |  |  | 1.11 |  |  |  |  |  |  |
| Flank pain | -0.30 | 0.01 |  |  |  |  |  |  |  |  |
| Flatulence | -3.21 | -2.71 |  |  |  |  |  |  |  |  |
| Fluid retention | -2.38 | -2.89 |  | -3.05 |  |  |  |  |  |  |
| Focal segmental glomerulosclerosis | -0.82 |  |  |  |  |  |  |  |  |  |
| Food poisoning | -1.93 |  |  |  |  |  |  |  |  |  |
| Frequent bowel movements | -0.78 |  |  |  |  |  |  |  |  |  |
| Fungal oesophagitis |  |  |  |  |  |  |  |  | 0.60 |  |
| Gastric cancer |  | -0.85 |  |  |  |  |  |  |  |  |
| Gastric disorder |  | -3.46 |  |  |  |  |  |  |  |  |
| Gastric haemorrhage |  |  |  | 0.08 |  |  |  |  |  |  |
| Gastric mucosal lesion |  | 1.11 |  |  |  |  |  |  |  |  |
| Gastric perforation | -0.38 | -0.57 |  |  |  |  |  |  |  |  |
| Gastric ulcer | -0.63 | -1.47 |  |  |  |  |  |  | -2.16 |  |
| Gastric ulcer haemorrhage | -0.76 | -2.64 |  |  | 1.92 |  |  |  |  |  |
| Gastritis | -0.29 | -0.11 |  |  |  |  | 0.50 |  | 2.71 | 2.19 |
| Gastritis erosive |  | -1.27 |  |  |  |  |  |  |  |  |
| Gastritis haemorrhagic | 0.83 |  |  |  |  |  |  |  |  |  |
| Gastroenteritis | -0.19 | -0.83 |  |  |  |  |  |  | 1.41 |  |
| Gastrointestinal disorder | -0.75 | -2.36 |  |  |  |  | 0.50 |  | -0.59 |  |
| Gastrointestinal haemorrhage | -1.49 | -1.85 |  | -1.65 |  |  |  |  | -2.58 |  |
| Gastrointestinal inflammation |  |  |  |  |  |  |  |  | -0.38 |  |
| Gastrointestinal necrosis | -0.79 |  |  |  |  |  |  |  |  |  |
| Gastrointestinal pain |  | -2.77 |  |  |  |  |  |  |  |  |
| Gastrointestinal perforation | 2.17 | 1.33 |  |  |  |  |  |  | 1.88 |  |
| Gastrointestinal toxicity | 0.92 |  |  |  |  |  |  |  | 2.36 |  |
| Gastrointestinal ulcer | -1.85 |  |  |  |  |  |  |  |  |  |
| Gingival bleeding | -1.65 |  |  |  |  |  |  |  |  |  |
| Gingival swelling | -2.40 |  |  |  |  |  |  |  |  |  |
| Glomerulonephritis | 0.85 | -0.26 |  |  |  |  |  |  |  |  |
| Glossitis |  | 0.69 |  |  |  |  |  |  |  |  |
| Granulomatosis with polyangiitis |  |  |  |  |  |  |  |  | -0.03 |  |
| Haematemesis | -0.63 | -1.33 |  |  |  |  |  |  | -3.01 |  |
| Haematochezia | -0.69 | -0.58 |  |  |  |  |  |  |  |  |
| Haematuria | -0.38 | -0.63 |  | 1.50 |  |  | -1.49 |  | -0.99 |  |
| Haemolytic uraemic syndrome | -2.22 |  |  |  |  |  |  |  | -1.19 |  |
| Hydronephrosis | 1.07 | 0.80 |  |  |  |  |  |  |  |  |
| Hyperparathyroidism secondary |  |  |  |  |  |  |  |  | -0.66 |  |
| Hypophagia | -1.26 | -0.60 |  |  |  |  |  |  | -1.51 |  |
| Iga nephropathy |  | -0.61 |  |  |  |  |  |  |  |  |
| Ileal perforation |  |  |  | 2.90 |  |  |  |  |  |  |
| Ileus | 1.54 | 0.86 |  | 1.10 |  |  | 0.42 |  | 1.10 |  |
| Ileus paralytic | 0.70 |  |  |  |  |  |  |  | -0.44 | 2.68 |
| Incontinence | -0.88 |  |  |  |  |  |  |  | -1.16 |  |
| Inflammatory bowel disease | -0.04 |  |  |  |  |  |  |  |  |  |
| Inguinal hernia | -0.55 |  |  |  |  |  |  |  |  |  |
| Intestinal obstruction | -0.57 | -0.70 |  |  |  | -0.76 | -1.39 |  | -0.54 |  |
| Intestinal perforation | 0.12 | 0.46 |  | 0.38 |  |  | 2.83 |  | 1.85 |  |
| Intestinal pseudo-obstruction |  |  |  |  |  |  |  |  | 0.64 |  |
| Intra-abdominal haemorrhage |  | -1.42 |  |  |  |  |  |  |  |  |
| Intussusception |  | 0.83 |  |  |  |  |  |  |  |  |
| Jejunal perforation | 0.39 | 0.76 |  |  |  |  |  |  |  |  |
| Kidney fibrosis | -1.09 |  |  |  |  |  | 0.79 |  | 0.40 |  |
| Kidney infection | -2.68 | -3.03 |  |  |  |  |  |  |  |  |
| Kidney transplant rejection | 1.38 | 0.60 |  |  |  |  |  |  | 0.36 | 1.66 |
| Large intestinal obstruction | -1.07 |  |  |  |  |  |  |  | 0.39 |  |
| Large intestine perforation | 0.13 | -0.20 |  |  |  |  |  |  | -0.13 |  |
| Lip oedema | -0.85 |  |  |  |  |  |  |  |  |  |
| Lip swelling |  | -1.72 |  |  |  |  |  |  |  |  |
| Malignant ascites |  | 0.42 |  |  |  |  |  |  |  |  |
| Malignant peritoneal neoplasm | 1.82 |  |  |  |  |  |  |  |  |  |
| Melaena | 0.32 | -0.82 |  |  |  |  |  |  | -2.33 |  |
| Metastases to kidney | 1.89 |  |  |  |  |  |  |  |  |  |
| Metastases to pancreas | 0.61 |  |  |  |  |  |  |  |  |  |
| Metastases to peritoneum | -0.17 |  |  |  |  |  |  |  |  |  |
| Metastatic renal cell carcinoma | -1.72 |  |  |  |  |  |  |  |  |  |
| Micturition urgency | -3.05 | -2.90 |  |  |  |  |  |  |  |  |
| Mouth haemorrhage | -0.75 |  |  |  |  |  |  |  |  |  |
| Mouth swelling |  | -1.05 |  |  |  |  |  |  |  |  |
| Mouth ulceration | -0.28 | -1.90 |  |  |  |  |  |  |  |  |
| Nausea | -1.14 | -0.78 |  | -1.17 |  | -1.59 | -0.68 |  | -0.48 |  |
| Nephritis | 3.06 | 3.82 |  | 3.52 |  |  | 0.07 |  | 3.37 |  |
| Nephrogenic anaemia | -1.83 |  |  |  |  |  |  |  |  |  |
| Nephrolithiasis | -3.94 | -3.73 |  |  |  |  |  |  |  |  |
| Nephropathy | -2.31 | -1.40 |  |  |  |  |  |  |  |  |
| Nephropathy toxic | -0.66 | -2.13 |  |  |  |  |  |  |  |  |
| Nephrotic syndrome | 1.40 | 0.21 |  |  |  |  |  |  | -1.29 |  |
| Neurogenic bladder | -0.95 |  |  |  |  |  |  |  |  |  |
| Oesophageal candidiasis | -0.89 |  |  |  |  |  |  |  |  |  |
| Oesophageal carcinoma |  | -0.63 |  |  |  |  |  |  |  |  |
| Oesophageal fistula | 2.43 |  |  |  |  |  |  |  |  |  |
| Oesophageal perforation |  | 0.92 |  |  |  |  |  |  |  |  |
| Oesophagitis | 0.54 | -0.48 |  |  |  |  |  |  | 0.41 |  |
| Oral candidiasis | 0.20 |  |  |  |  |  |  |  | -0.05 |  |
| Oral herpes | -1.06 |  |  |  |  |  |  |  |  |  |
| Oral lichen planus | -0.88 | 0.40 |  |  |  |  |  |  |  |  |
| Oral mucosa erosion | -0.97 |  |  |  |  |  |  |  |  |  |
| Oral mucosal blistering | -0.64 | -1.24 |  |  |  |  |  |  |  |  |
| Oral pain | -1.97 |  |  |  |  |  |  |  |  |  |
| Oropharyngeal pain | -2.26 |  |  |  |  |  |  |  |  |  |
| Pancreatic atrophy | 2.33 | 0.84 |  |  |  |  | 1.09 |  | 0.17 |  |
| Pancreatic disorder | -0.18 |  |  |  |  |  |  |  |  |  |
| Pancreatic failure |  |  |  |  |  |  |  |  | 1.02 |  |
| Pancreatitis | 0.27 | 0.03 |  | 1.04 | 0.78 | 1.28 |  |  | 0.91 |  |
| Pancreatitis acute | -0.95 | -2.06 |  |  |  |  |  |  |  |  |
| Parotitis |  | -0.70 |  |  |  |  |  |  |  |  |
| Pelvic pain | -4.37 |  |  |  |  |  |  |  |  |  |
| Peritonitis | -0.70 |  |  | -1.35 |  |  |  |  |  |  |
| Pharyngitis | -2.22 |  |  |  |  |  |  |  |  |  |
| Pneumatosis intestinalis | 0.89 |  |  |  |  |  |  |  |  |  |
| Pneumoperitoneum |  | -1.18 |  |  |  |  |  |  |  |  |
| Pollakiuria | -1.23 | -2.79 |  |  |  |  |  |  |  |  |
| Polyuria | -1.83 |  |  |  |  |  |  |  |  |  |
| Prerenal failure | 0.72 |  |  |  |  |  |  |  |  |  |
| Proctitis | 0.46 |  |  | 1.92 |  |  |  |  | 1.68 |  |
| Proteinuria | -0.53 | -1.55 |  |  |  |  |  |  |  |  |
| Pyelonephritis |  |  |  |  |  |  | -1.06 |  |  |  |
| Rectal haemorrhage | -2.19 |  |  |  |  | -2.28 |  |  | -1.94 |  |
| Renal cancer | -3.71 |  |  |  |  |  |  |  |  |  |
| Renal cyst | -2.76 |  |  |  |  |  |  |  |  |  |
| Renal disorder | 0.49 | -0.73 |  |  |  |  |  |  | -1.38 |  |
| Renal failure | -0.58 | -1.10 |  | -0.49 | -0.61 |  | -3.41 |  | -0.87 |  |
| Renal haemorrhage | -1.81 |  |  |  |  |  |  |  |  |  |
| Renal impairment | 0.05 | 0.18 |  | -0.71 |  |  | -1.16 |  | -1.74 |  |
| Renal infarct | -1.35 |  |  |  |  |  |  |  |  |  |
| Renal injury | -1.98 | -3.37 |  | -2.98 |  |  | -3.26 |  | -3.88 |  |
| Renal pain | -3.14 |  |  |  |  |  |  |  |  |  |
| Renal tubular disorder |  | -2.05 |  |  |  |  |  |  | -0.06 |  |
| Renal tubular necrosis | -2.50 | -1.25 |  |  |  |  |  |  |  |  |
| Retroperitoneal fibrosis |  | -0.04 |  |  |  |  |  |  |  |  |
| Sialoadenitis | 1.28 |  |  |  |  |  |  |  | 0.52 |  |
| Sjogren syndrome | 1.13 | 2.07 |  |  |  |  |  |  | 2.63 |  |
| Small intestinal obstruction | -1.01 | -0.90 |  | 1.29 |  |  |  |  | 0.15 |  |
| Small intestinal perforation | 0.11 | 1.70 |  |  |  |  |  |  |  |  |
| Stomatitis | 0.31 | -0.46 |  |  |  |  |  |  | -2.13 |  |
| Swollen tongue | -2.02 |  |  |  |  |  |  |  |  |  |
| Systemic scleroderma |  | 1.44 |  |  |  |  |  |  |  |  |
| Thrombotic microangiopathy |  | -3.31 |  |  |  |  |  |  |  |  |
| Tooth disorder | -2.83 |  |  |  |  |  |  |  | -4.10 |  |
| Tubulointerstitial nephritis | -0.38 | 0.32 |  | -0.32 |  |  | -0.95 |  | 0.39 |  |
| Urinary incontinence | -1.92 | -2.08 |  |  |  |  |  |  |  |  |
| Urinary retention | -0.71 | -1.10 |  | -0.38 |  |  | -2.97 |  | -2.71 |  |
| Urinary tract disorder | -2.88 |  |  |  |  |  |  |  |  |  |
| Urinary tract infection | -1.58 | -2.30 |  | -0.76 |  |  | -3.59 |  | -1.12 |  |
| Urinary tract obstruction | -2.01 | -0.64 |  |  |  |  |  |  | 0.39 |  |
| Urosepsis | -2.19 | -3.06 |  | 1.15 |  |  |  |  | -0.92 |  |
| Varices oesophageal | -1.24 |  |  |  |  |  |  |  |  |  |
| Vomiting | -1.19 | -1.14 |  | -1.66 |  | -0.37 | -0.91 |  | -0.62 |  |

*In Table S6, nivo, pemb, cemi, atez, avel, durv, ipil, poly1, poly2 and poly3 were respectively represent nivolumab, pembrolizumab, cemiplimab, atezolizumab, avelumab, durvalumab, ipilimumab, nivolumab+ ipilimumab+ pembrolizumab, nivolumab+ ipilimumab and ipilimumab+ pembrolizumab.

# Table S7 IC025 of the association between class-specific ICIs monotherapy/polytherapy (with chemotherapy) and renal AEs in FAERS database

| **PT** | **Nivo** | **Pemb** | **Cemi** | **Atez** | **Avel** | **Durv** | **Ipil** | **Poly1** | **Poly2** | **Poly3** |
| --- | --- | --- | --- | --- | --- | --- | --- | --- | --- | --- |
| Abdominal pain | 0.84 |  |  | 0.53 |  |  |  |  | 0.76 |  |
| Abdominal pain upper | -0.96 |  |  |  |  |  |  |  |  |  |
| Acute kidney injury | 1.79 | 0.22 |  | 1.32 | -0.58 | -0.46 | -0.38 |  | 2.62 |  |
| Ascites | 0.63 |  |  | 0.82 |  |  |  |  |  |  |
| Autoimmune colitis |  |  |  |  |  |  |  |  | 3.00 |  |
| Bladder mass |  |  |  |  |  |  |  |  |  | 4.06 |
| Chronic kidney disease | -4.01 |  |  |  |  |  |  |  |  |  |
| Colitis | 2.19 | 2.46 |  | 3.07 |  |  |  |  | 3.08 | 0.41 |
| Constipation |  | -0.22 |  |  |  |  |  |  |  |  |
| Diarrhoea | 0.25 | -0.03 |  | -0.13 |  | -1.24 |  |  | 0.87 |  |
| Diverticulitis | -1.42 |  |  |  |  |  |  |  |  |  |
| Duodenal ulcer | -0.13 |  |  |  |  |  |  |  |  |  |
| Dysgeusia |  | 0.32 |  |  |  |  |  |  |  |  |
| Dysphagia | -0.61 |  |  |  |  |  |  |  |  |  |
| End stage renal disease |  | -1.51 |  |  |  |  |  |  |  |  |
| Enteritis |  |  |  | 1.24 |  |  |  |  | 2.17 |  |
| Enterocolitis | 2.62 |  |  |  |  |  |  |  | 3.42 |  |
| Gastroenteritis |  |  |  |  |  |  |  |  | 1.43 |  |
| Intestinal obstruction |  |  |  | 0.94 |  |  |  |  |  |  |
| Nausea | -0.90 | -0.30 |  |  | -0.19 |  |  |  | -0.43 |  |
| Nephritis |  |  |  | 0.96 |  |  |  |  |  |  |
| Oesophageal candidiasis |  |  |  | 0.08 |  |  |  |  |  |  |
| Oral candidiasis | 1.54 |  |  |  |  |  |  |  |  |  |
| Renal failure | -3.36 | -1.56 |  | -1.27 | -1.65 |  |  |  | 0.36 |  |
| Renal impairment | -2.94 | -0.42 |  | -0.64 |  |  |  |  |  |  |
| Renal injury | -2.57 |  |  |  |  |  |  |  |  |  |
| Small intestinal obstruction |  | 0.15 |  | 1.57 |  |  |  |  |  |  |
| Stomatitis |  | 0.08 |  |  |  |  |  |  | -0.86 |  |
| Tubulointerstitial nephritis |  |  |  | 1.05 |  |  |  |  |  |  |
| Urinary tract infection |  |  |  | -1.24 | 0.41 |  |  |  | -1.87 |  |
| Vomiting | -0.86 | -0.51 |  | -2.42 |  |  |  |  | -0.80 |  |

*In Table S7, nivo, pemb, cemi, atez, avel, durv, ipil, poly1, poly2 and poly3 were respectively represent nivolumab, pembrolizumab, cemiplimab, atezolizumab, avelumab, durvalumab, ipilimumab, nivolumab+ ipilimumab+ pembrolizumab, nivolumab+ ipilimumab and ipilimumab+ pembrolizumab.


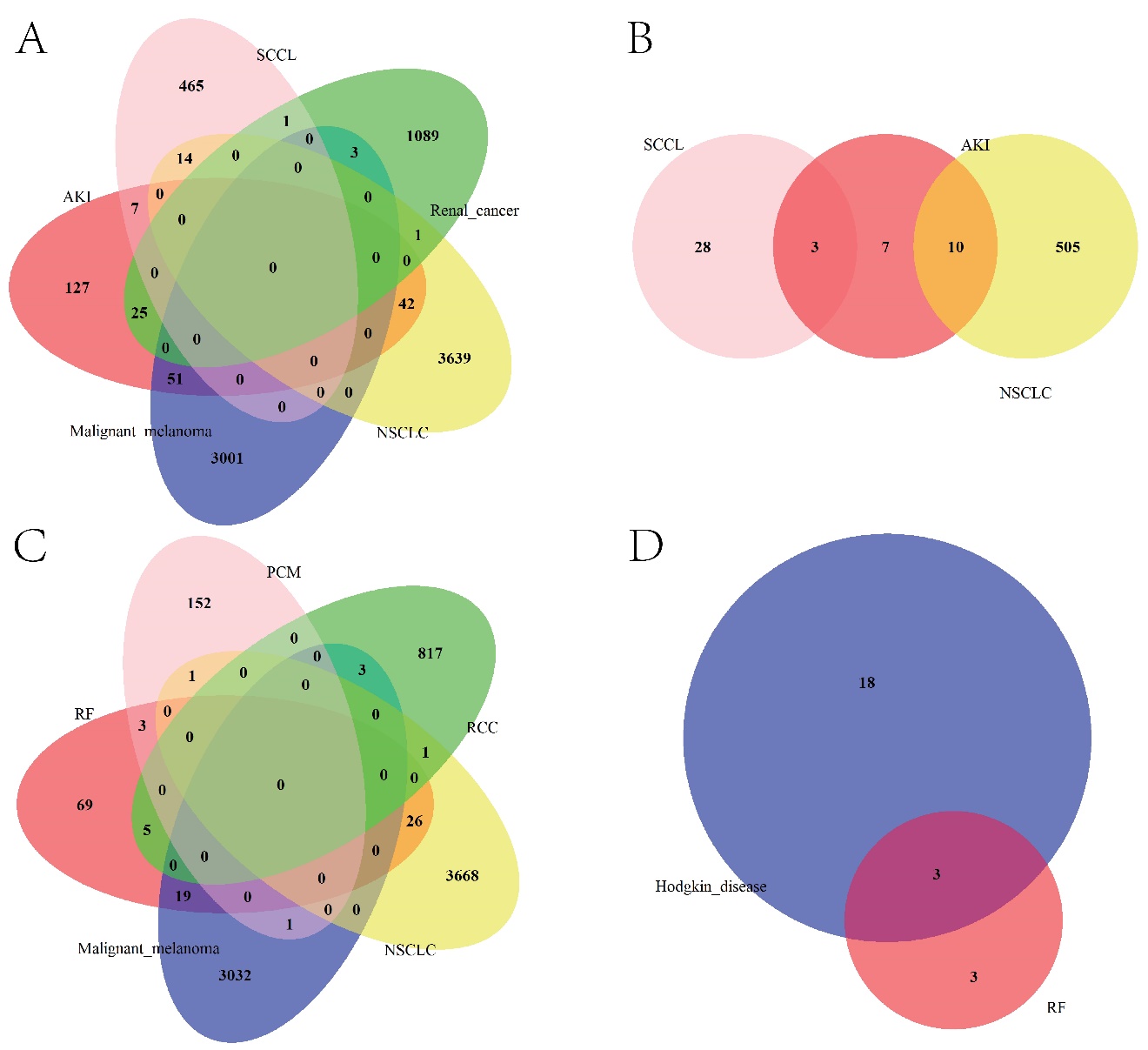


# Figure S1 A. Venn diagram between acute kidney injury and the death outcome cases in the top 4 most frequently related cancers (without chemotherapy). B. Venn diagram between acute kidney injury and the death outcome cases in the top 2 most frequently related cancers (with chemotherapy). C. Venn diagram between renal failure and the death outcome cases in the top 4 most frequently related cancers (without chemotherapy). D. Venn diagram between renal failure and the death outcome cases in the top 1 most frequently related cancers (with chemotherapy). In Figure S1, AKI, RF, SCCL, NSCLC, PCM and RCC were respectively represent acute kidney injury, renal failure, squamous cell carcinoma of lung, non-small cell lung cancer, plasma cell myeloma and renal cell carcinoma.


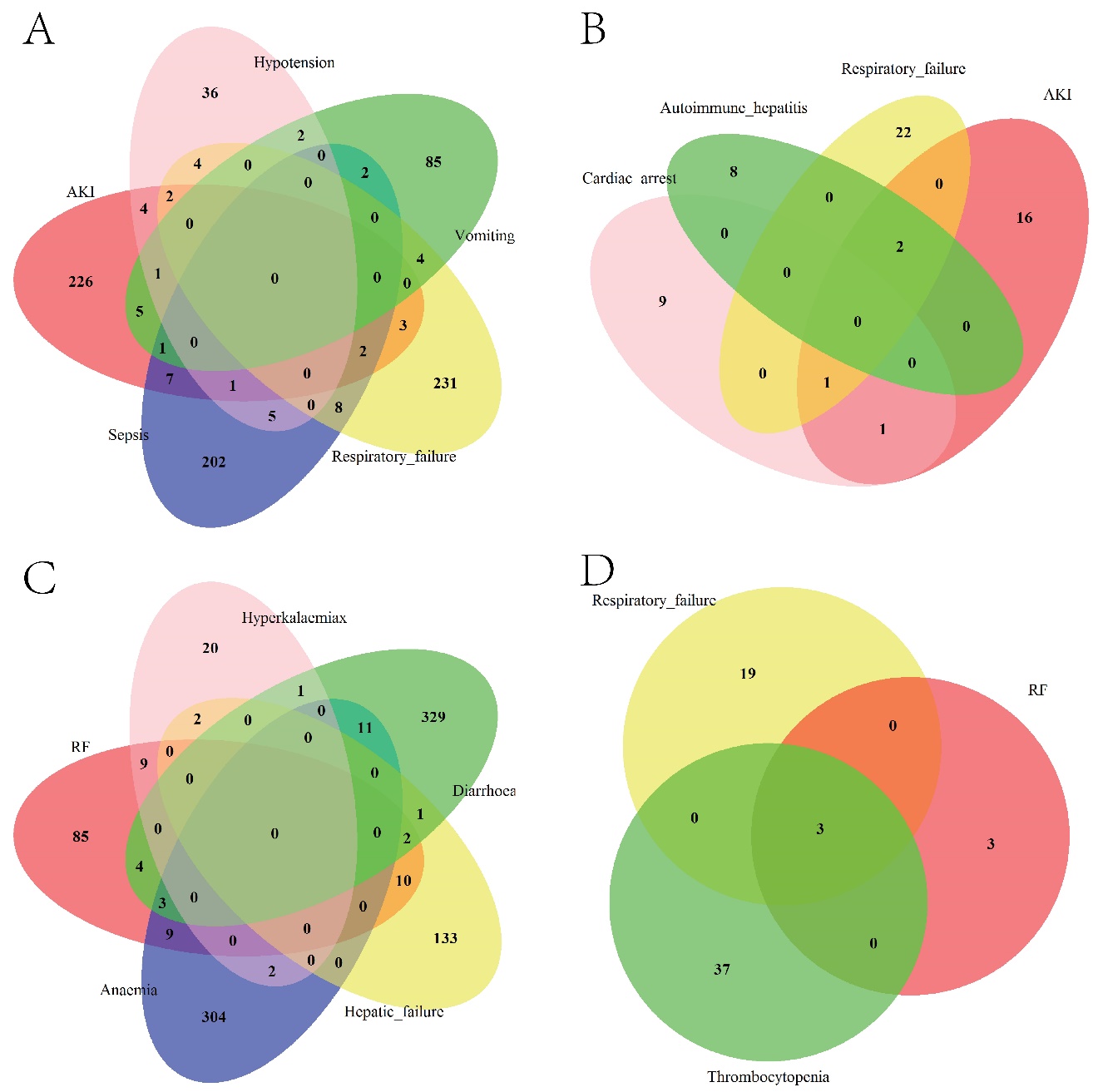


# Figure S2 A. Venn diagram between acute kidney injury and the death outcome cases in the top 4 most frequently related AEs (without chemotherapy). B. Venn diagram between acute kidney injury and the death outcome cases in the top 3 most frequently related AEs (with chemotherapy). C. Venn diagram between renal failure and the death outcome cases in the top 4 most frequently related AEs (without chemotherapy). D. Venn diagram between renal failure and the death outcome cases in the top 2 most frequently related AEs (with chemotherapy). In Figure S2, AKI and RF were respectively represent acute kidney injury and renal failure.
